# Supplementary material for: Validation of asthma recording in the Clinical Practice Research Datalink (CPRD)
Source: BMJ Open. 2017 Aug 11;7(8):e017474. doi: 10.1136/bmjopen-2017-017474 (PMC5724126; doi:10.1136/bmjopen-2017-017474)
Supplement: Supplementary appendix 1 [file bmjopen-2017-017474supp001.pdf]

Appendix 1: CPRD medcodes indicating asthma

A) Specific asthma codes

medcode	readterm
78	asthma
81	asthma monitoring
185	acute exacerbation of asthma
232	asthma attack
233	severe asthma attack
1555	bronchial asthma
2290	allergic asthma
3018	mild asthma
3366	severe asthma
3458	occasional asthma
3665	late onset asthma
4442	asthma unspecified
4606	exercise induced asthma
4892	status asthmaticus nos
5267	intrinsic asthma
5627	hay fever with asthma
5798	chronic asthmatic bronchitis
5867	exercise induced asthma
6707	extrinsic asthma with asthma attack
7058	emergency admission, asthma
7146	extrinsic (atopic) asthma
7191	asthma limiting activities
7378	asthma management plan given
7416	asthma disturbing sleep
7731	pollen asthma
8335	asthma attack nos
8355	asthma monitored
9018	number of asthma exacerbations in past year
9552	change in asthma management plan
9663	step up change in asthma management plan
10043	asthma annual review
10274	asthma medication review
10487	asthma - currently active
11370	asthma confirmed
12987	late-onset asthma
13064	asthma severity
13065	moderate asthma
13175	asthma disturbs sleep frequently
13176	asthma follow-up

14777	extrinsic asthma without status asthmaticus
15248	hay fever with asthma
16070	asthma nos
16667	asthma control step 2
16785	asthma control step 1
18223	step down change in asthma management plan
18224	asthma control step 3
18323	intrinsic asthma with asthma attack
19167	asthma monitoring by nurse
19519	asthma treatment compliance unsatisfactory
19520	asthma treatment compliance satisfactory
20860	asthma control step 5
20886	asthma control step 4
21232	allergic asthma nec
22752	occupational asthma
24479	emergency asthma admission since last appointment
24506	further asthma - drug prevent.
24884	asthma causes daytime symptoms 1 to 2 times per week
25181	asthma restricts exercise
25791	asthma clinical management plan
26501	asthma never causes daytime symptoms
26503	asthma causes daytime symptoms most days
26504	asthma never restricts exercise
26506	asthma severely restricts exercise
26861	asthma sometimes restricts exercise
27926	extrinsic asthma with status asthmaticus
29325	intrinsic asthma without status asthmaticus
30458	asthma monitoring by doctor
30815	asthma causing night waking
31167	asthma night-time symptoms
31225	asthma causes daytime symptoms 1 to 2 times per month
38143	asthma never disturbs sleep
38144	asthma limits walking up hills or stairs
38145	asthma limits walking on the flat
38146	asthma disturbs sleep weekly
39478	wood asthma
39570	asthma causes night symptoms 1 to 2 times per month
40823	brittle asthma
41017	aspirin induced asthma
41020	absent from work or school due to asthma
42824	asthma daytime symptoms
45073	intrinsic asthma nos

45782	extrinsic asthma nos
46529	attends asthma monitoring
47337	asthma accident and emergency attendance since last visit
47684	detergent asthma
58196	intrinsic asthma with status asthmaticus
73522	work aggravated asthma
93353	sequoiosis (red-cedar asthma)
93736	royal college of physicians asthma assessment
98185	asthma control test
99793	patient has a written asthma personal action plan
100107	health education - asthma self management
100397	asthma control questionnaire
100509	under care of asthma specialist nurse
100740	health education - structured asthma discussion
102170	asthma review using roy colleg of physicians three questions
102209	mini asthma quality of life questionnaire
102301	asthma trigger - seasonal
102341	asthma trigger - pollen
102395	asthma causes symptoms most nights
102400	asthma causes night time symptoms 1 to 2 times per week
102449	asthma trigger - respiratory infection
102713	asthma limits activities 1 to 2 times per month
102871	asthma trigger - exercise
102888	asthma limits activities 1 to 2 times per week
102952	asthma trigger - warm air
103318	health education - structured patient focused asthma discuss
103321	asthma trigger - animals
103612	asthma never causes night symptoms
103631	royal college physician asthma assessment 3 question score
103813	asthma trigger - cold air
103944	asthma trigger - airborne dust
103945	asthma trigger - damp
103952	asthma trigger - emotion
103955	asthma trigger - tobacco smoke
103998	asthma limits activities most days
105420	asthma self-management plan review
105674	asthma self-management plan agreed
106805	chronic asthma with fixed airflow obstruction
107167	number days absent from school due to asthma in past 6 month

B) Non-specific asthma codes

medcode	readterm
719	h/o: asthma
1208	childhood asthma
5138	patient in asthma study
5515	seen in asthma clinic
7229	asthma prophylactic medication used
11022	asthma trigger
11387	refuses asthma monitoring
11673	excepted from asthma quality indicators: patient unsuitable
11695	excepted from asthma quality indicators: informed dissent
13066	asthma - currently dormant
13173	asthma not disturbing sleep
13174	asthma not limiting activities
16655	asthma monitoring admin.
18141	asthma monitoring due
18692	exception reporting: asthma quality indicators
18763	referral to asthma clinic
19539	asthma monitoring check done
20422	asthma clinic administration
25705	asthma monitor 3rd letter
25706	asthma monitor 2nd letter
25707	asthma monitor 1st letter
25796	mixed asthma
26496	health education - asthma
29645	asthma control step 0
30308	dna - did not attend asthma clinic
30382	asthma monitoring admin.nos
31135	asthma monitor phone invite
35927	asthma leaflet given
37943	asthma monitor verbal invite
41554	asthma monitor offer default
43770	asthma society member
92109	asthma outreach clinic
