# Supplementary material for: Validation of asthma recording in the Clinical Practice Research Datalink (CPRD)
Source: BMJ Open. 2017 Aug 11;7(8):e017474. doi: 10.1136/bmjopen-2017-017474 (PMC5724126; doi:10.1136/bmjopen-2017-017474)
Supplement: Supplementary appendix 2 [file bmjopen-2017-017474supp002.pdf]

Study into asthma: questionnaire for £55, further information for £55

The London School of Hygiene and Tropical Medicine is conducting a study to investigate the best way to identify asthma within the Clinical Practice Research Datalink (CPRD). We have developed several methods for identifying asthma in the database, and we would like to obtain some information on the current asthma status of the patient from GPs so that we can decide which method is the most suitable.

We would be very grateful if you could supply us with the following information.

A. Do you agree this patient has a current diagnosis of asthma?

- ☐ Yes: Proceed to question B
- ☐ No: Proceed to question C
- ☐ Uncertain: Proceed to question B

If you answered yes or uncertain to question A:

B1. Has the diagnosis been made or confirmed by a respiratory physician?

- ☐ Yes
- ☐ No

B2. Does this patient have evidence of reversible airway obstruction?

- ☐ Yes
- ☐ No

If yes: **Was this based on;**

- ☐ Spirometry reversibility with a bronchodilator
- ☐ Trial of treatment with oral or inhaled corticosteroids and diurnal variation on a peak flow diary

B3. In what year was the asthma first diagnosed?

B4. Were any other factors taken into consideration in making the diagnosis?

		Yes	No
a.	History of atopic disorder	☐	☐
b.	Family history of asthma and/or atopic disorder	☐	☐
c.	Widespread wheeze heard on auscultation of the chest	☐	☐
d.	Otherwise unexplained low FEV (Forced Expiratory Volume) or PEF (Peak Expiratory Flow) on spirometry	☐	☐
e.	*Otherwise unexplained variability in PEFR (Peak Expiratory Flow Rate) on spirometry*	☐	☐

f.	Otherwise unexplained peripheral blood eosinophilia	☐	☐
g.	FeNO (Fractional exhaled Nitric Oxide) measurement	☐	☐
h.	Other (please name)		

B5. Based on the QOF (Quality and Outcomes Framework) indicators:

		**Yes**	**No**
a.	Does the patient have any difficulty sleeping because of asthma symptoms, including cough	☐	☐
b.	Does the patient have the usual asthma symptoms during the day (cough, wheeze, chest tightness of breathlessness)?	☐	☐
c.	Does the asthma interfere with the patient's usual activities (housework, work, school, etc.)?	☐	☐

B6. What is the patient's smoking status?

☐ Current smoker

☐ Ex-smoker

☐ Never-smoker

B7. Does the patient have any other respiratory diseases? (Multiple responses possible)

☐ Chronic Obstructive Pulmonary Disease (COPD)

☐ Bronchiectasis

☐ Interstitial Lung Disease

☐ Other, please list:

☐ No

If you answered no to question A:

C. Do you think this patient has a history of asthma?

☐ Yes

☐ No

☐ Uncertain

Please provide anonymised copies of any additional relevant information allowing corroborating asthma diagnosis e.g. medical notes, discharge letters, test values. Payment for further information is £55 per patient.

Please return responses to CPRD in the freepost envelope provided or to our freepost address:

Freepost RSKH-TTAU-UKKX, CPRD, MHRA,

151 Buckingham Palace Rd, London, SW1W 9SZ
