# Supplementary material for: Validation of asthma recording in the Clinical Practice Research Datalink (CPRD)
Source: BMJ Open. 2017 Aug 11;7(8):e017474. doi: 10.1136/bmjopen-2017-017474 (PMC5724126; doi:10.1136/bmjopen-2017-017474)
Supplement: Supplementary appendix 3 [file bmjopen-2017-017474supp003.pdf]

ISAC APPLICATION FORM
PROTOCOLS FOR RESEARCH USING THE CLINICAL PRACTICE RESEARCH DATALINK (CPRD)

ISAC use only:<br>Protocol Number<br>Date submitted	 ....<br>.............................	**IMPORTANT**<br>**If you have any queries, please contact ISAC Secretariat:**<br>ISAC@cprd.com

Section A: The study

1. Study Title
Validation of the recording of asthma diagnosis in adult patients in the Clinical Practice Research Datalink

2. Has any part of this research proposal or a related proposal been previously submitted to ISAC?
Yes ☐ No ☒
If Yes, please provide previous protocol numbers:

3. Has this protocol been peer reviewed by another Committee? (e.g. grant award or ethics committee)
Yes ☒ No ☐
If Yes, please state the name of the reviewing Committee(s) and provide an outline of the review process and outcome: Internal review by GSK, PRF committee

4. Type of Study (please tick all the relevant boxes which apply)

Adverse Drug Reaction/Drug Safety ☐
Drug Effectiveness ☐
Health/Public Health Services Research ☒
Drug Utilisation ☐
Pharmacoeconomics ☐
Disease Epidemiology ☒
Methodological ☒
Post-authorisation Safety ☐
Other* ☐
*Please specify the type of study in the lay summary

5. This study is intended for (please tick all the relevant boxes which apply)**:**

Publication in peer reviewed journals ☒
Presentation at company/institutional meetings ☒
Other
Presentation at scientific conference ☒
Regulatory purposes ☐

Section B: The Investigators

6. Chief Investigator (full name, job title, organisation name & e-mail address for correspondence- see guidance notes for eligibility)
Jennifer Quint, Clinical Senior lecturer in Respiratory epidemiology, Imperial College London, j.quint@imperial.ac.uk
CV has been previously submitted to ISAC ☒ **CV number:** 042_15CEPSL
A new CV is being submitted with this protocol ☐
An updated CV is being submitted with this protocol ☐

7. Affiliation (full address)
Department of NCDE, LSHTM, Keppel Street, London WC1E 7HT

8. Corresponding Applicant
Francis Nissen, PhD researcher, LSHTM, francis.nissen@lshtm.ac.uk
Same as chief investigator ☐
CV has been previously submitted to ISAC ☒ **CV number:** 449_15S
A new CV is being submitted with this protocol ☐
An updated CV is being submitted with this protocol ☐

9. List of all investigators/collaborators (*please list the full names, affiliations and e-mail addresses* of all collaborators, other than the Chief Investigator*)

Other investigator: Ian Douglas, Senior lecturer, LSHTM, ian.douglas@lshtm.ac.uk
CV has been previously submitted to ISAC ☒ **CV number:** 157_15CESL
A new CV is being submitted with this protocol ☐
An updated CV is being submitted with this protocol ☐

Other investigator: Liam Smeeth, LSHTM, Liam.Smeeth@lshtm.ac.uk
CV has been previously submitted to ISAC ☒ **CV number:** 045_15CEPSL
A new CV is being submitted with this protocol ☐
An updated CV is being submitted with this protocol ☐

Other investigator: Hana Müllerova, GSK, hana.x.muellerova@gsk.com
CV has been previously submitted to ISAC ☒ **CV number:** 365_15E
A new CV is being submitted with this protocol ☐
An updated CV is being submitted with this protocol ☐

Other investigator: Daniel Morales, University of Dundee, d.r.z.morales@dundee.ac.uk
CV has been previously submitted to ISAC ☒ **CV number:** 450_15P
A new CV is being submitted with this protocol ☐
An updated CV is being submitted with this protocol ☐

Other investigator: Neil Pearce, LSHTM, Neil.Pearce@lshtm.ac.uk
CV has been previously submitted to ISAC ☒ **CV number:** 367_15CS
A new CV is being submitted with this protocol ☐
An updated CV is being submitted with this protocol ☐

[Please add more investigators as necessary] *Please note that your ISAC application form and protocol* ***must*** *be copied to all e-mail addresses listed above at the time of submission of your application to the ISAC mailbox. Failure to do so will result in delays in the processing of your application.*

10. Conflict of interest statement* (please provide a draft of the conflict (or competing) of interest (COI) statement that you intend to include in any publication which might result from this work)

All authors have completed the ICMJE uniform disclosure at www.icmje.org/coi_disclosure.pdf and declare:
FN has received a PhD scholarship from GSK
Dr Quint reports grants from MRC, GSK, BLF, Wellcome. Personal fees from AZ, GSK.
ID has consulted for and holds stock in GSK
**Please refer to the International Committee of Medical Journal Editors (ICMJE) for guidance on what constitutes a COI*

11. Experience/expertise available (please complete the following questions to indicate the experience/expertise available within the team of investigators/collaborators actively involved in the proposed research, including the analysis of data and interpretation of results

	**Previous GPRD/CPRD Studies**	**Publications using GPRD/CPRD data**
None	☐	☐
1-3	☐	☐
> 3	☒	☒

	**Yes**	**No**
**Is statistical expertise available within the research team?**<br>*If yes, please indicate the name(s) of the relevant investigator(s)*<br>Ian Douglas	☒	☐
**Is experience of handling large data sets (>1 million records) available within the research team?**<br>*If yes, please indicate the name(s) of the relevant investigator(s)*<br>Ian Douglas<br>Jennifer Quint<br>Liam Smeeth<br>Daniel Morales	☒	☐
**Is experience of practising in UK primary care available within the research team?**<br>*If yes, please indicate the name(s) of the relevant investigator(s)*<br>Liam Smeeth<br>Daniel Morales	☒	☐

12. References relating to your study

Please list up to 3 references (most relevant) relating to your proposed study:

Quint JK, Müllerova H, DiSantostefano RL, Forbes H, Eaton S, Hurst JR, Davis K, Smeeth L.: Validation of chronic obstructive pulmonary disease recording in the Clinical Practice Research Datalink (CPRD-GOLD). BMJ Open. 2014 Jul 23;4(7)

Cornish RP, Henderson J, Boyd AW, Granell R, Van Staa T, Macleod: Validating childhood asthma in an epidemiological study using linked electronic patient records. J. BMJ Open. 2014 Apr 23;4(4)

British Thoracic Society Scottish Intercollegiate Guidelines Network. British guideline on the management of asthma. Thorax 2008;63(Suppl 4):iv1–121.

Section C: Access to the data

13. Financial Sponsor of study

Pharmaceutical Industry	☒ *Please specify:* GSK	Academia	☐ *Please specify:*
Government / NHS	☐ *Please specify:*	Charity	☐ *Please specify:*
Other	☐ *Please specify:*	None	☐

14. Type of Institution carrying out the analyses

Pharmaceutical Industry	☐ *Please specify:*	Academia	☒ *Please specify:* LSHTM
Government Department	☐ *Please specify:*	Research Service Provider	☐ *Please specify:*
NHS	☐ *Please specify:*	Other	☐ *Please specify:*

15. Data source

The sponsor has direct access to CPRD GOLD and will extract the relevant data* ☒

A data set will be supplied by CPRD** ☐

CPRD has been commissioned to extract the relevant data and to perform the analyses ☐

Other ☐ *Please specify:*

*If data sources other than CPRD GOLD are required, these will be supplied by CPRD
** Please note that datasets provided by CPRD are limited in size. Applicants should contact CPRD (KC@CPRD.com) if a dataset of >300,000 patients is required.

16. Primary care data (please specify which primary care data set(s) are required)

Vision only (Default for CPRD studies) ☒
EMIS® only* ☐
Both Vision and EMIS®* ☐

Note: Vision and EMIS are different clinical systems, Vision data has traditionally been used for CPRD, EMIS is currently undergoing beta-testing.
**Investigators requiring the use of EMIS data must discuss the study with a member of CPRD staff before submitting an ISAC application*
Please list below the name of the person/s at the CPRD with whom you have discussed your request for EMIS data:

Section D: Data linkage

17. Does this protocol also seek access to data held under the CPRD Data Linkage Scheme?

Yes* ☒ No ☐

If No, please move to section E.

**Investigators requiring linked data must discuss the study with a member of CPRD staff. It is important to be aware that linked data are not available for all patients in CPRD GOLD, the coverage periods for each data source may differ and charges may be applied. Please contact the CPRD Research Team on +44 (20) 3080 6383 or email kc@cprd.com to discuss your requirements before submitting your application.*
Please list below the name of the person/s at the CPRD with whom you have discussed your request:

Please note that as part of the ISAC review of linkages, the protocol may be shared - in confidence - with a representative of the requested linked data set(s) and summary details may be shared - in confidence - with the Confidentiality Advisory Group of the Health Research Authority.

18. Please select the source(s) of linked data being requested:

☐ ONS Mortality Data
☐ Inpatient Hospital Episode Statistics
☐ Outpatient Hospital Episode Statistics
☐ NCDR Cancer Registry Data*
☐ MINAP
☐ Mother Baby Link

☒ Index of Multiple Deprivation
☐ Townsend Score
☐ Other** *Please specify:*

We have discussed the data linkages with Rachael Williams, Research Statistician at CPRD.

Please note that applicants seeking access to cancer registry data must provide consent for publication of their study title and study institution on the UK Cancer Registry website. They must also complete a **Cancer Dataset Agreement Form (available from CPRD) and provide a **System level Security Policy** for each organisation involved in the study.*

*** If "Other" is specified, please name an individual in CPRD that this linage has been discussed with.*

19. Total number of linked datasets requested including CPRD GOLD: 1

20. Is linkage to a local dataset with <1 million patients being requested?

Yes* ☐ No ☒

** If yes, please provide further details:*

21. If you have requested linked data sets, please indicate whether the Chief Investigator or any of the collaborators listed in response to question 5 above, have access to any of the linked datasets in a patient identifiable form, or associated with a patient index.

Yes* ☐ No ☒

** If yes, please provide further details:*

22. Does this study involve linking to patient *identifiable* data from other sources?

Yes ☐ No ☒

Section E: Validation/verification

23. Does this protocol describe a purely observational study using CPRD data (this may include the review of anonymised free text)?

Yes* ☒ No** ☐

** Yes: If you will be using data obtained from the CPRD Group, this study does not require separate ethics approval from an NHS Research Ethics Committee.*
*** No: You may need to seek separate ethics approval from an NHS Research Ethics Committee for this study. The ISAC will provide advice on whether this may be needed.*

24. Does this study require anonymised free text?

Yes* ☐ No ☒

**Please note that work involving free text can only be performed on the July 2013 CPRD GOLD database build or earlier versions. CPRD can provide further advice on the use of anonymised free text.*

25. Does this protocol involve requesting any additional information from GPs?

Yes* ☒ No ☐

* *Please indicate what will be required:*

Completion of questionnaires by the GP$^{\psi}$ Yes ☒ No ☐
Provision of anonymised records (e.g. hospital discharge summaries) Yes ☒ No ☐
Other (please describe)

$^{\psi}$ Any questionnaire for completion by GPs or other health care professional must be approved by ISAC before circulation for completion.

26. Does this study require contact with patients in order for them to complete a questionnaire?

Yes* ☐ No ☒

**Please note that any questionnaire for completion by patients must be approved by ISAC before circulation for completion.*

27. Does this study require contact with patients in order to collect a sample?

Yes* ☐ No ☒

** Please state what will be collected:*

Section F: Signatures

28. Signature from the Chief Investigator

I confirm that the above information is to the best of my knowledge accurate, and I have read and understood the guidance to applicants.

Name: Jennifer Quint Date: 08/12/2015 E. signature (type name): Jennifer Quint

Protocol Section

<u>The following headings **must** be used to form the basis of the protocol. Pages should be numbered. All abbreviations must be defined on first use.</u>

A. Lay Summary (Max. 200 words)

This study will investigate the recording of the diagnosis of asthma in the primary care medical records database called Clinical Practice Research Datalink (CPRD GOLD). This will be done by the collection of information provided by general practitioners through a questionnaire. This information will then be examined by two independent expert physicians, giving a reliable diagnosis to be compared with the recording of asthma within the CPRD database. The diagnosis of asthma is mostly based on a characteristic pattern of symptoms and the absence of another diagnosis. Because of this, asthma is not as well defined as some other diseases, and the clinical diagnosis might be less accurate. The study to be undertaken could help establish the best strategy to identify individuals with asthma within the CPRD. This would inform the definitions and patient selection for further observational and potentially pragmatic intervention studies in CPRD and other primary care data sources.

B. Technical Summary (Max. 200 words)

The overall aim of this study is to determine the positive predictive value (PPV) of different algorithms using asthma diagnostic Read codes within the CPRD GOLD, i.e., a proportion of true positives among those assumed to have been diagnosed with asthma. In order to achieve this we will construct a retrospective cohort of asthma patients and compare database information (CPRD GOLD and the Multiple Deprivation Index) with information gathered by a questionnaire filled in by general practitioners and review of any supporting information sent. A review of these questionnaires by two independent expert physicians will be considered as the gold standard to assess the PPV of an asthma recording using specific algorithms in CPRD GOLD.

C. Objectives, Specific Aims and Rationale

(i) *Aim:*
To assess strategies to identify asthma patients of adults in United Kingdom electronic primary care records.

(ii) *Objectives:*
To determine the PPV of the recording of asthma diagnosis of adults within the CPRD GOLD database.

(iii) *Rationale:*
We will measure the level of accuracy, using the PPV, of an asthma diagnosis recording in the CPRD database employing a gold standard comprised of the review of general practitioners questionnaires by two independent experts. By doing so, we will be able to assess how reliable an asthma diagnosis is in electronic primary care records.

D. Background

Asthma is difficult to assess in health-care database epidemiological studies as the diagnostic criteria are based on non-specific respiratory symptoms and variable expiratory airflow limitation which are often not recorded in electronic medical records (1). According to the current estimates of the Global Burden of Disease Study 2013, 334 million people worldwide have asthma. 8.6% of young adults (aged 18-45) experience asthma symptoms and 4.5% of young adults worldwide have been diagnosed with asthma and/or are taking treatment for asthma (2). In the UK, 5.4 million people are currently receiving treatment for asthma, of whom 4.3 million are adults (3).

The British guideline on the management of asthma states that the diagnosis in adults is based on the recognition of a characteristic pattern of symptoms and signs and the absence of an alternative explanation. Based on clinical features that either increase or decrease the probability of asthma, patients are categorized in the "low", "intermediate" or "high" probability groups. The diagnosis is then confirmed or rejected based on spirometry and/or a trial of treatment with corticosteroids (1).

Chronic obstructive pulmonary disorder (COPD), another respiratory obstructive disease that has a lot of symptoms in common with asthma can be identified with high PPV from the CPRD datasets using diagnostic Read codes alone (PPV=80%) or combined with COPD medications (PPV=90%) (4). The characteristic of COPD that best distinguishes it from asthma is the degree of reversibility of airflow obstruction, which is a central question in the questionnaire to be sent out to the GP's (see appendix 2).

As the clinical examination necessary for the diagnosis of asthma is time and resource demanding, it would be useful for epidemiological studies to be able to obtain accurate records of asthma diagnosis within electronic databases of health-care records. The goal of this study is to understand and quantify how accurate asthma recording is in CPRD. When subsequent studies would be performed, it will be better understood how well the data reflects true diagnoses of asthma. A validation study of childhood asthma using General Practice Research Database (GPRD) data by using parental reports of a doctor's diagnosis as the gold standard has been conducted and found a high sensitivity and specificity (5). A different study in Canada has validated asthma in patients older than 16 by comparing different information fields in electronic primary healthcare records without an external comparison (6). The CPRD database has been used in asthma studies because it captures a broad range of patients and goes back a long time. The current study will focus on the accuracy of asthma diagnosis recording in adults in CPRD, by measuring the PPV of different algorithms within the CPRD database and comparing it to a gold standard diagnosis given by the review of the answers of the GP questionnaire.

E. Study Type

This is a methodological study.

F. Study Design

This is a validation study of strategies or algorithms to ascertain asthma diagnosis recordings conducted in a retrospective cohort of asthma patients from the CPRD GOLD.

The random sample of individuals to be included in the study will be constructed from all participants registered in CPRD on or after 1 January 2004 who meet the inclusion criteria (see below). For the main analysis, a patient will be able to contribute to one algorithm only if an asthma medcode was recorded within the 24 month window prior to the end of data collection. It is possible an individual will be eligible for more than one algorithm depending on the Read codes used in their medical record. The individuals will be randomly selected from the algorithm with the fewest participants first and then removed from the cohort so that they cannot be selected for another algorithm. We have chosen this strategy (as opposed to an individual being eligible for a single algorithm only) because we want to test strategies to identify asthma patients from a single cohort rather than to test validity of the diagnosis. Further studies could then use a single strategy or their combination to extract an asthma cohort. There will be no special measures to ensure less frequent Read codes are used, because we assume the validity of asthma diagnosis strategy would be not be different between common and less frequent Read codes and the quality of recording would also be comparable. In addition, less frequent Read codes are unlikely to be used in isolation; our experience with validation of COPD recordings had shown that these infrequent Read codes are usually accompanied by other types of recordings.

Sample Size

The number of records for whom an asthma monitoring plan was started (medcode 81) exceeds 500,000 and the total number of asthma-related consultations exceeds 9,000,000 in the CPRD database.

Assuming an estimated PPV of 0.85 for each algorithm and an accuracy of the PPV (95% CI ± 0.08), a sample size of 77 individuals for each algorithm is needed.
A similar study conducted for COPD had a 77.6% response rate and 73.2% of the sent questionnaires were fit to be included in the final analysis (4). Considering a random sample of fully completed responses of 77 asthma patients for 8 algorithms is needed with 15% extra to account for a potential lower response rate, 750 questionnaires in total will be sent.

G. Data Linkage Required (if applicable)

The data linkage of CPRD-GOLD to MDI (Multiple Deprivation Index) is required to gather more information on the socio-economic status of the studied records. Ideally the MDI would be on patient level, if this is not available then the MDI on practice level would be used. We wish to request access to the IMD data linked to both the postcode of the GP practice and the patient’s residential address (2010 version). We will take patient eligibility for linkage to IMD data into account when selecting our study population. We will also take the differences in methodology in IMD between the different countries of the UK into account.

H. Study Population

Inclusion Criteria

- Over 18 years old. People who become 18 after the study start can be included if they meet the criteria of an algorithm.
- Acceptable user status registered in CPRD.
- Practice is “up to standard” at study start 1/1/2004. From this date onwards, the Quality and Outcomes Framework (QOF) came in effect.

- The patient fits in one of the asthma algorithms within the last 24 months (see below)
- Patients are still alive and practice is currently still active in the CPRD.

Exclusion Criteria:

The patient does not fit the criteria of an algorithm group
Younger than 18 years

I. Selection of comparison group(s) or controls

There is no comparison group, as this is a validation study. The cohort will consist of only patients with a recording of asthma.

J. Exposures, Outcomes and Covariates

Exposure: Each patient included can contribute to only one algorithm or strategy (see appendix 3). If a patient is selected for a single algorithm (starting with the algorithm with the fewest participants), the patient will be excluded from the pool for the next algorithms. A preliminary code list for asthma diagnosis can also be found in appendix 1.
Covariates for stratification analysis:

-Age in years. All patients are 18 years or older, the categories will be based on the sample distribution.
-Gender as male or female
-Body Mass Index (BMI)
-Smoking status
-Other co-morbid conditions: COPD, atopy, GERD (Gastro-oesophageal Reflux Disease), eczema,rhinitis (including allergic rhinitis (hayfever) and chronic rhinosinusitis) and family history of asthma or atopy.
-Multiple deprivation Index

Outcome: recording of asthma diagnosis according to a specified algorithm and verified by the reference standard.

A number of different algorithms were constructed with degrees of certainty of asthma using separate indicators (see appendix 3). For example, the most stringent algorithm would include an asthma code, asthma medication and demonstrated reversibility after trial of treatment. Other algorithms would then drop one or more of these criteria. See appendix 3 for details of the algorithms.

A questionnaire will be sent to the general practitioners of a random sample of patients who fit in a certain algorithm to obtain information for the gold standard. A draft of the questionnaire can be found in appendix 2. The questionnaire is based on the "British guideline on the management of asthma" by the British Thoracic Society and Scottish Intercollegiate Guidelines Network (1).

K. Data/ Statistical analysis

The main analysis will be the calculation of the positive predictive value (the proportion of true positives) in each of the predefined algorithms. The gold standard consists of the opinion of 2 medical experts (Jennifer Quint and Daniel Morales) independently

reviewing the questionnaires and any additional supporting medical information provided. If there is a disagreement of diagnosis, the case would be discussed by the two experts. If an agreement cannot be found, a third opinion will be sought. Included in the main text.
Stratification analysis will be used to assess potential effect modification or confounding by covariates (see covariate list).

L. Plan for addressing confounding

Not applicable.

M. Plan for addressing missing data

We plan to do a complete case analysis, assuming that the probability of data being missing is independent of accuracy of the asthma diagnosis, conditional on covariates. If the amount of missing data is small, any violation of the assumption is unlikely to importantly affect the results. We anticipate a small degree of missingness for the BMI and smoking covariates.

N. Limitations of the study design, data sources and analytical methods

-Using a GP questionnaire as the source of patient information in order to obtain a gold standard to validate the asthma diagnosis can be problematic as the GP can consult the electronic health record to see if there was an asthma diagnosis. This will lead to an overestimation of the PPV. The GP's will be asked not to consult the CPRD records in the questionnaire.
-Incomplete diagnostic information will lead to missing data which we will be unaware of which could lead to some inaccuracy in PPV or classification of asthma probability.
-Only living patients will be assessed, as GP's no longer have access to the patient records after death. This excludes the records of the deceased patients and could result in survival bias.
-Miscoding accidents would lower the PPV.
-Response rate for the questionnaire might be lower than expected, and the sample size of the completed questionnaires could be too small.
-By focusing on the PPV, we will not be able to accurately assess the NPV, specificity or sensitivity. By preselecting the population of possible asthma cases, the NPV, specificity and sensitivity would be artificially manipulated. The NPV is the Negative Predictive Value: the proportion of negative results that are true negatives. -We are assuming that the validity of asthma diagnosis strategy would not be different between common and less frequent Read codes and the quality of recording would also be comparable for pragmatic reasons. In future practice when identifying patients with asthma, the less commonly used codes will continue to identify a smaller proportion of all asthma patients and so the validity we measure will apply to the majority of patients.
-We are also assuming that the probability of data being missing is independent of accuracy of the asthma diagnosis. We agree this assumption may not hold, but, we are even less likely to meet the assumptions needed for multiple imputation. However, we anticipate little missing relevant data in this study based on past research. In addition, the covariates are needed for stratification analysis only, rather than for adjustment. So we anticipate the impact of missing data to be low

-Not all GP practices contribute to CPRD, and patients might refuse to participate in the CPRD programme. This can result in selection bias.

O. Patient or user group involvement (if applicable)

Currently there is no plan to involve patients in the study. Depending on our findings it is possible we would seek patient engagement in further studies to help shape future research questionswith the help of general asthma patient groups.

P. Plans for disseminating and communicating study results, including the presence or absence of any restrictions on the extent and timing of publication

We will present our findings at national and international meetings and publish the results in a peer reviewed journal. We will not include any cells with counts less than five due to anonymity concerns.

Q. References

1. British Thoracic Society Scottish Intercollegiate Guidelines N. British Guideline on the Management of Asthma. Thorax. 2008;63 Suppl 4:iv1-121.
2. Global, regional, and national age–sex specific all-cause and cause-specific mortality for 240 causes of death, 1990–2013: a systematic analysis for the Global Burden of Disease Study 2013. The Lancet.385(9963):117-71.
3. NHS. http://www.nhs.uk/conditions/asthma: NHS; 2015 [cited 2015 06/11].
4. Quint JK, Mullerova H, DiSantostefano RL, Forbes H, Eaton S, Hurst JR, et al. Validation of chronic obstructive pulmonary disease recording in the Clinical Practice Research Datalink (CPRD-GOLD). BMJ Open. 2014;4(7):e005540.
5. Cornish RP, Henderson J, Boyd AW, Granell R, Van Staa T, Macleod J. Validating childhood asthma in an epidemiological study using linked electronic patient records. BMJ Open. 2014;4(4):e005345.
6. Xi N, Wallace R, Agarwal G, Chan D, Gershon A, Gupta S. Identifying patients with asthma in primary care electronic medical record systems: Chart analysis–based electronic algorithm validation study. Canadian Family Physician. 2015;61(10):e474-e83.

R. Amendment

March 2016

There were some slight changes to the questionnaire on advice from CPRD regarding the remuneration of the GP's. There were also some minor amendments to the questionnaire to clarify the procedure for returning the questionnaire and to insert the patient identifier tables we use. The sentence "To answer this questionnaire, please refrain from using the data recorded in CPRD as the aim of this study is to see how reliable CPRD is." was removed to avoid confusion.

March 2017

We would like to examine the additional information provided by the questionnaires sent to GP's to quantify the misdiagnosis of COPD in asthma patients in the UK. The symptoms of asthma and COPD overlap, and the differential diagnosis is not always trivial to make. Information on reversibility testing, the QOF indicators, smoking status, concurrent respiratory diseases and other sources including consultant and hospital discharge letters, lung function tests and radiography results was requested in the questionnaire (see attachment).
A review of this information by a respiratory consultant and study GP aims to identify the actual cases of COPD in confirmed asthma patients. This review is used as the gold standard to calculate the PPV, NPV, sensitivity and specificity of recorded GP diagnoses of COPD in the primary care records of asthma patients.

The specific objectives we would like to add to this study are to calculate the PPV, NPV, sensitivity and specificity of a COPD diagnosis recorded by a general practitioner in patients with a confirmed asthma diagnosis.

Appendices

Appendix 1: CPRD medcodes indicating asthma

medcode	readterm	Probable	Definite
78	asthma		1
81	asthma monitoring		1
185	acute exacerbation of asthma		1
232	asthma attack		1
233	severe asthma attack		1
719	h/o: asthma	1	
1208	childhood asthma	1	
1555	bronchial asthma		1
2290	allergic asthma		1
3018	mild asthma		1
3366	severe asthma		1
3458	occasional asthma		1
3665	late onset asthma		1
4442	asthma unspecified		1
4606	exercise induced asthma		1
4892	status asthmaticus nos		1
5138	patient in asthma study	1	
5267	intrinsic asthma		1
5515	seen in asthma clinic	1	
5627	hay fever with asthma		1
5798	chronic asthmatic bronchitis		1
5867	exercise induced asthma		1
6707	extrinsic asthma with asthma attack		1
7058	emergency admission, asthma		1
7146	extrinsic (atopic) asthma		1
7191	asthma limiting activities		1
7229	asthma prophylactic medication used	1	
7378	asthma management plan given		1
7416	asthma disturbing sleep		1
7731	pollen asthma		1
8335	asthma attack nos		1
8355	asthma monitored		1
9018	number of asthma exacerbations in past year		1
9552	change in asthma management plan		1
9663	step up change in asthma management plan		1
10043	asthma annual review		1
10274	asthma medication review		1

10487	asthma - currently active		1
11022	asthma trigger	1	
11370	asthma confirmed		1
11387	refuses asthma monitoring	1	
11673	excepted from asthma quality indicators: patient unsuitable	1	
11695	excepted from asthma quality indicators: informed dissent	1	
12987	late-onset asthma		1
13064	asthma severity		1
13065	moderate asthma		1
13066	asthma - currently dormant	1	
13173	asthma not disturbing sleep	1	
13174	asthma not limiting activities	1	
13175	asthma disturbs sleep frequently		1
13176	asthma follow-up		1
14777	extrinsic asthma without status asthmaticus		1
15248	hay fever with asthma		1
16070	asthma nos		1
16655	asthma monitoring admin.	1	
16667	asthma control step 2		1
16785	asthma control step 1		1
18141	asthma monitoring due	1	
18223	step down change in asthma management plan		1
18224	asthma control step 3		1
18323	intrinsic asthma with asthma attack		1
18692	exception reporting: asthma quality indicators	1	
18763	referral to asthma clinic	1	
19167	asthma monitoring by nurse		1
19519	asthma treatment compliance unsatisfactory		1
19520	asthma treatment compliance satisfactory		1
19539	asthma monitoring check done	1	
20422	asthma clinic administration	1	
20860	asthma control step 5		1
20886	asthma control step 4		1
21232	allergic asthma nec		1
22752	occupational asthma		1
24479	emergency asthma admission since last appointment		1
24506	further asthma - drug prevent.		1
24884	asthma causes daytime symptoms 1 to 2 times per week		1
25181	asthma restricts exercise		1
25705	asthma monitor 3rd letter	1	
25706	asthma monitor 2nd letter	1	
25707	asthma monitor 1st letter	1	
25791	asthma clinical management plan		1
25796	mixed asthma	1	

26496	health education - asthma	1	
26501	asthma never causes daytime symptoms		1
26503	asthma causes daytime symptoms most days		1
26504	asthma never restricts exercise		1
26506	asthma severely restricts exercise		1
26861	asthma sometimes restricts exercise		1
27926	extrinsic asthma with status asthmaticus		1
29325	intrinsic asthma without status asthmaticus		1
29645	asthma control step 0	1	
30308	dna - did not attend asthma clinic	1	
30382	asthma monitoring admin.nos	1	
30458	asthma monitoring by doctor		1
30815	asthma causing night waking		1
31135	asthma monitor phone invite	1	
31167	asthma night-time symptoms		1
31225	asthma causes daytime symptoms 1 to 2 times per month		1
35927	asthma leaflet given	1	
37943	asthma monitor verbal invite	1	
38143	asthma never disturbs sleep		1
38144	asthma limits walking up hills or stairs		1
38145	asthma limits walking on the flat		1
38146	asthma disturbs sleep weekly		1
39478	wood asthma		1
39570	asthma causes night symptoms 1 to 2 times per month		1
40823	brittle asthma		1
41017	aspirin induced asthma		1
41020	absent from work or school due to asthma		1
41554	asthma monitor offer default	1	
42824	asthma daytime symptoms		1
43770	asthma society member	1	
45073	intrinsic asthma nos		1
45782	extrinsic asthma nos		1
46529	attends asthma monitoring		1
47337	asthma accident and emergency attendance since last visit		1
47684	detergent asthma		1
58196	intrinsic asthma with status asthmaticus		1
73522	work aggravated asthma		1
92109	asthma outreach clinic	1	
93353	sequoiosis (red-cedar asthma)		1
93736	royal college of physicians asthma assessment		1
98185	asthma control test		1
99793	patient has a written asthma personal action plan		1
100107	health education - asthma self management		1
100397	asthma control questionnaire		1

100509	under care of asthma specialist nurse		1
100740	health education - structured asthma discussion		1
102170	asthma review using roy colleg of physicians three questions		1
102209	mini asthma quality of life questionnaire		1
102301	asthma trigger - seasonal		1
102341	asthma trigger - pollen		1
102395	asthma causes symptoms most nights		1
102400	asthma causes night time symptoms 1 to 2 times per week		1
102449	asthma trigger - respiratory infection		1
102713	asthma limits activities 1 to 2 times per month		1
102871	asthma trigger - exercise		1
102888	asthma limits activities 1 to 2 times per week		1
102952	asthma trigger - warm air		1
103318	health education - structured patient focused asthma discuss		1
103321	asthma trigger - animals		1
103612	asthma never causes night symptoms		1
103631	royal college physician asthma assessment 3 question score		1
103813	asthma trigger - cold air		1
103944	asthma trigger - airborne dust		1
103945	asthma trigger - damp		1
103952	asthma trigger - emotion		1
103955	asthma trigger - tobacco smoke		1
103998	asthma limits activities most days		1
105420	asthma self-management plan review		1
105674	asthma self-management plan agreed		1
106805	chronic asthma with fixed airflow obstruction		1
107167	number days absent from school due to asthma in past 6 month		1

Study into asthma: questionnaire for £55, further information for £55

The London School of Hygiene and Tropical Medicine is conducting a study to investigate the best way to identify asthma within the Clinical Practice Research Datalink (CPRD). We have developed several methods for identifying asthma in the database, and we would like to obtain some information on the current asthma status of the patient from GPs so that we can decide which method is the most suitable. We would be very grateful if you could supply us with the following information.

A. Do you agree this patient has a current diagnosis of asthma?

☐ Yes: Proceed to question B
☐ No: Proceed to question C
☐ Uncertain: Proceed to question B

If you answered yes or uncertain to question A:

B1. Has the diagnosis been made or confirmed by a respiratory physician?

☐ Yes
☐ No

B2. Does this patient have evidence of reversible airway obstruction?

☐ Yes
☐ No

If yes: **Was this based on;**

☐ Spirometry reversibility with a bronchodilator
☐ Trial of treatment with oral or inhaled corticosteroids and diurnal

variation on a peak flow diary

B3. In what year was the asthma first diagnosed?

B4. Were any other factors taken into consideration in making the diagnosis?

	**Yes**	**No**
a. History of atopic disorder	☐	☐
b. Family history of asthma and/or atopic disorder	☐	☐
c. Widespread wheeze heard on auscultation of the chest	☐	☐
d. Otherwise unexplained low FEV (Forced Expiratory Volume) or PEF (Peak Expiratory Flow) on spirometry	☐	☐
e. Otherwise unexplained variability in PEFR (Peak Expiratory Flow Rate) on spirometry	☐	☐
f. Otherwise unexplained peripheral blood eosinophilia	☐	☐

g. FeNO (Fractional exhaled Nitric Oxide) measurement ☐ ☐
h. Other (please name)

B5. Based on the QOF (Quality and Outcomes Framework) indicators:

	**Yes**	**No**
a. Does the patient have any difficulty sleeping because of asthma symptoms, including cough	☐	☐
b. Does the patient have the usual asthma symptoms during the day (cough, wheeze, chest tightness of breathlessness)?	☐	☐
c. Does the asthma interfere with the patient's usual activities (housework, work, school, etc.)?	☐	☐

B6. What is the patient's smoking status?

☐ Current smoker
☐ Ex-smoker
☐ Never-smoker

B7. Does the patient have any other respiratory diseases? (Multiple responses possible)

☐ Chronic Obstructive Pulmonary Disease (COPD)
☐ Bronchiectasis
☐ Interstitial Lung Disease
☐ Other, please list:
☐ No

If you answered no to question A:

C. Do you think this patient has a history of asthma?

☐ Yes
☐ No
☐ Uncertain

Please provide anonymised copies of any additional relevant information allowing corroborating asthma diagnosis e.g. medical notes, discharge letters, test values. Payment for further information is £55 per patient.

Please return responses to CPRD in the freepost envelope provided or to our freepost address:
Freepost RSKH-TTAU-UKKX, CPRD, MHRA,
151 Buckingham Palace Rd, London, SW1W 9SZ

Appendix 3: Algorithms: all within the last 24 months

1. Definite asthma code + evidence of reversibility testing (spirometry or trial of treatment) *or variable PEFR* + more than one prescription of inhaled asthma therapy *(Inhaled SABA/LABA/CS)*
2. Definite asthma code + evidence of reversibility testing (spirometry or trial of treatment) *or variable PEFR*
3. Definite asthma code + more than one prescription of inhaled asthma therapy *(Inhaled SABA/LABA/CS)*
4. Definite asthma code only
5. Possible asthma code + evidence of reversibility testing (spirometry or trial of treatment) *or variable PEFR* + more than one prescription of inhaled asthma therapy *(Inhaled SABA/LABA/CS)*
6. Symptoms (wheeze, breathlessness, chest tightness, cough) + evidence of reversibility testing (spirometry or trial of treatment) *or variable PEFR* + more than one prescription of inhaled asthma therapy *(Inhaled SABA/LABA/CS)*
7. Symptoms (wheeze, breathlessness, chest tightness, cough) + evidence of reversibility testing (spirometry or trial of treatment) or variable PEFR
8. Symptoms (wheeze, breathlessness, chest tightness, cough) + more than one prescription of inhaled asthma therapy *(Inhaled SABA/LABA/CS)*
