# Supplementary material for: Validation of asthma recording in the Clinical Practice Research Datalink (CPRD)
Source: BMJ Open. 2017 Aug 11;7(8):e017474. doi: 10.1136/bmjopen-2017-017474 (PMC5724126; doi:10.1136/bmjopen-2017-017474)
Supplement: Author's manuscript [file bmjopen-2017-017474.draft_revisions.pdf]

BMJ Open

BMJ Open is committed to open peer review. As part of this commitment we make the peer review history of every article we publish publicly available.

When an article is published we post the peer reviewers' comments and the authors' responses online. We also post the versions of the paper that were used during peer review. These are the versions that the peer review comments apply to.

The versions of the paper that follow are the versions that were submitted during the peer review process. They are not the versions of record or the final published versions. They should not be cited or distributed as the published version of this manuscript.

BMJ Open is an open access journal and the full, final, typeset and author-corrected version of record of the manuscript is available on our site with no access controls, subscription charges or pay-per-view fees (http://bmjopen.bmj.com).

If you have any questions on BMJ Open's open peer review process please email info.bmjopen@bmj.com

BMJ Open

Validation of asthma recording in the Clinical Practice Research Datalink (CPRD)

Journal:	*BMJ Open*
Manuscript ID	bmjopen-2017-017474
Article Type:	Research
Date Submitted by the Author:	25-Apr-2017
Complete List of Authors:	Nissen, Francis; London School of Hygiene and Tropical Medicine, EPH - ENCD<br>Morales, Daniel; University of Dundee, 2Division of Population Health Sciences<br>Müllerová, Hana; GlaxoSmithKline, RWE & Epidemiology, GSK R&D<br>Smeeth, Liam; London School of Hygiene and Tropical Medicine, Epidemiology and Population Health<br>Douglas, Ian; London School of Hygiene and Tropical Medicine, Epidemiology and Population Health<br>Quint, Jennifer; Imperial College London, Respiratory Epidemiology, Occupational Medicine and Public Health
**Primary Subject Heading**:	Epidemiology
Secondary Subject Heading:	Respiratory medicine, Health informatics, General practice / Family practice
Keywords:	Asthma < THORACIC MEDICINE, EPIDEMIOLOGY, Health informatics < BIOTECHNOLOGY & BIOINFORMATICS, Information management < BIOTECHNOLOGY & BIOINFORMATICS

SCHOLARONE™
Manuscripts

Validation of asthma recording in the Clinical Practice Research Datalink (CPRD)

Authors: Francis Nissen,[1] Daniel R.Morales,[2] Hana Mullerova,[3] Liam Smeeth,[1] Ian J Douglas,[1] Jennifer K Quint[4]

[1]Department of Non-Communicable Disease Epidemiology, London School of Hygiene and Tropical Medicine, London, UK; [2]Division of Population Health Sciences, University of Dundee, Dundee, UK; [3]RWE & Epidemiology, GSK R&D, Uxbridge, UK, [4]National Heart and Lung Institute, Imperial College, London, UK;

Correspondence:

Francis Nissen, MD, MSc, Department of Non-Communicable Disease Epidemiology, London School of Hygiene and Tropical Medicine, London, UK.

Address: Keppel Street, London, WC1E 7HT, UK

E-mail: francis.nissen@lshtm.ac.uk

+44 786 4314 923

For peer review only - http://bmjopen.bmj.com/site/about/guidelines.xhtml

ABSTRACT

Objectives: The optimal method of identifying people with asthma from electronic health records in primary care is not known. The aim of this study is to determine the Positive Predictive Value (PPV) of different algorithms using clinical codes and prescription data to identify people with asthma in the United Kingdom Clinical Practice Research Datalink (CPRD).

Methods: 684 participants registered with a GP practice contributing to CPRD between 1st of December 2013 and 30th of November 2015 were selected according to 1 of 8 pre-defined potential asthma identification algorithms. A questionnaire was sent to the general practitioners to confirm asthma status and provide additional information to support an asthma diagnosis. Two study physicians independently reviewed and adjudicated the questionnaires and additional information to form a gold standard for asthma diagnosis. The Positive Predictive Value was calculated for each algorithm.

Results: 684 questionnaires were sent, of which 494 (72%) were returned and 475 (69%) were complete and analysed. All 5 algorithms including a specific Read code indicating asthma or non-specific Read code accompanied by additional conditions performed well. The PPV for asthma diagnosis using only a specific asthma code was 86.4% (95% CI 77.4% to 95.4%). Extra information on asthma medication prescription (PPV 83.3%), evidence of reversibility testing (PPV 86.0%) or a combination of all three selection criteria (PPV 86.4%)

For peer review only - http://bmjopen.bmj.com/site/about/guidelines.xhtml

did not result in a higher PPV. The algorithm using non-specific asthma codes, information on reversibility testing, and respiratory medication use scored highest (PPV 90.7%, 95% CI (82.8% to 98.7%), but had a much lower identifiable population. Algorithms based on asthma symptom codes had low PPV's (43.1% to 57.8%).

Conclusions: People with asthma can be accurately identified from UK primary care records using specific Read codes. The inclusion of spirometry or asthma medications in the algorithm did not clearly improve accuracy.

Keywords

Asthma, Validation, Electronic Health Records, Positive Predictive Value, epidemiology

Word count: 3287

Article summary

Strengths:

This study describes algorithms to identify people with asthma from CPRD, a large electronic health records database, and measures the positive predictive value of those algorithms.

For peer review only - http://bmjopen.bmj.com/site/about/guidelines.xhtml

A validated definition of asthma in CPRD allows for better informed health-care service planning by increasing the reliability of evidence generated from observational studies.

Supporting information, including outpatient referral letters, other emergency department discharge letters, airflow measurements and radiography records were used to identify asthma patients and calculate the test measures.

We measured the accuracy of a general practicioner diagnosis of asthma using questionnaires and additional information.

Limitations:

The gold standard to calculate a PPV (GP questionnaire and review by study physicians) is not absolute, even though information from secondary care was used.

A GP can look in the electronic health record to see if a specific diagnosis of asthma has been recorded, but there is no suitable practical alternative.

GP's of patients with complicated medical histories could be less likely to return the questionnaire, but remuneration makes this less likely.

We could not calculate the NPV, specificity or sensitivity as we had preselected our population of possible asthma cases.

For peer review only - http://bmjopen.bmj.com/site/about/guidelines.xhtml

BACKGROUND

Asthma is one of the most common chronic diseases, with an estimated prevalence of 241 million people worldwide with asthma (1). Cough, wheeze, breathlessness and chest tightness are its core symptoms (2) but it has a wide variety of different presentations (3).

Electronic health records (EHR) have been adopted worldwide, facilitating the construction of large population-based patient databases that have become available over the last decades for epidemiological research (4). Validation of diagnoses or outcomes based upon codes recorded in EHRs is required because their accuracy is uncertain, and this may affect the reliability and validity of subsequent observational studies. The quality of studies generated from EHRs may be debatable unless their data are validated for specific research purposes (5-8).

The diagnosis of asthma relies on clinical judgement based on a combination of patient history, physical examination and confirmation of the variability or reversibility of airflow obstruction using airflow measurements. This can make it difficult to assess the accuracy of asthma diagnoses in EHR-based epidemiological studies as some symptoms and airflow measurements may not be recorded. In addition, individuals affected by asthma can vary greatly in their presentation and symptoms are sometimes similar to other respiratory diseases such as COPD (Chronic Obstructive Pulmonary Disease) (9, 10).

For peer review only - http://bmjopen.bmj.com/site/about/guidelines.xhtml

The aim of this study was to test the accuracy of different approaches to identifying asthma in the United Kingdom Clinical Practice Research Datalink (CPRD) using the positive predictive value (PPV), by comparing the database records with a gold standard constructed from a review by 2 study physicians based on information provided by asthma patients' GPs.

For peer review only

For peer review only - http://bmjopen.bmj.com/site/about/guidelines.xhtml

METHODS

Dataset

The Clinical Practice Research Datalink (CPRD) is a large UK primary care database containing anonymised data on the people registered with primary care practices from across the UK. CPRD is representative of the UK population with regard to age and sex (11, 12). Within CPRD, diagnostic accuracy has been demonstrated to be high for many conditions and diseases, including COPD (13-16). CPRD contains detailed clinical information on diagnoses, prescriptions, laboratory tests, symptoms and hospital referrals, in addition to basic sociodemographic information recorded by the general practitioners. These general practitioners (GPs) act as primary care providers and gatekeepers for other National Health Service services, and information from other healthcare providers is also transmitted back to the GP. Clinical events and diagnoses are coded as Read codes, a dictionary of clinical terms widely used in the UK National Health Services by both primary and secondary healthcare providers. Validation studies aid to ensure credibility and quality of epidemiological studies done in CPRD (7).

For peer review only - http://bmjopen.bmj.com/site/about/guidelines.xhtml

Inclusion criteria

The study population consisted of people who had a record for a Read code indicating possible asthma in the two years before the index date (1st of December 2015) and who were registered in a GP practice meeting CPRD quality criteria. The Read code list is included in appendix 1. The data collection was planned before the index test and reference standard were performed. This timespan was chosen for several reasons: to overcome potential changes in quality of asthma diagnosis and recording over time; to reduce the chance that the database records were out of date; and to ensure the medical records were still available to GPs. People were identified at random based on one of eight pre-defined algorithms exclusively, which means that we populated the algorithm resulting in the smallest population first and subsequently removed these people from the cohort, to prevent them from also being selected for another algorithm. We randomly selected 800 possible asthma cases for validation. Of these, 116 asthma cases were excluded because their GP no longer participated with CPRD at the time questionnaires were sent to the clinicians for validation, as shown in figure 1. Due to changes in CPRD data governance after the start of the study it was not possible to select replacement patients.

GP questionnaire

CPRD mailed a two page questionnaire to the GPs of the people selected for inclusion as described above, requesting confirmation of current asthma diagnosis and additional information to support this diagnosis. This questionnaire can be found in the appendix. The questionnaire was designed to ascertain the diagnosis of asthma and verify the date of diagnosis. The questions included evidence of reversible airway obstruction, current symptoms, smoking history, respiratory comorbidities and Quality Outcome Framework (QOF) indicators. QOF is a national financial incentive scheme for GPs in the UK encouraging regular disease indicator measurement and recording. Asthma is one of the included diseases, and its indicators including airflow measurements and interference with work and night's rest (17).

Specific information available from the medical record including spirometry printouts and hospital respiratory outpatient letters were also requested. Data were encrypted twice to ensure anonymity, between practices and CPRD and also from CPRD to researchers. A questionnaire was considered invalid if it was returned blank or every question was answered "unknown".

Codelists and algorithms

Lists of medical codes (Read codes) deemed as specific and non-specific for asthma based on study physicians' opinion were created prior to the start of the study. Read codes are a hierarchical clinical coding system that are used in general practice in the UK and are entered by the GP into a computer programme called Vision. Each Read code is linked to a specific string of text, which refers to a single diagnosis or symptom. These data are then uploaded by CPRD after they have been processed and quality checked. The list of codes used for specific or definite asthma codes and nonspecific or probable asthma codes can be found in the appendix.

Combinations of Read codelists, evidence of reversibility testing and respiratory medication use were used to make up the eight algorithms. The first four algorithms required a specific asthma diagnosis code, with the first three requiring additional documentation consisting of either respiratory medication use and/or evidence of reversibility testing. The fifth algorithm required a non-specific asthma code and additional documentation of both respiratory medications and reversibility testing; the last three algorithms required respiratory symptom codes indicating asthma symptoms with additional information. The presence of spirometry for inclusion in an algorithm was based on the existence of a specific spirometry Read code in the records rather than an examination of said spirometry, although where spirometry traces were provided as part of the additional information, they were examined. Evidence of reversibility testing only refers to whether airflow measurements or trial of treatment were

done, and does not reflect the results of these tests. Respiratory medication use was defined as at least two prescriptions of asthma medication for inhaled asthma therapy (Short Acting Beta-Agonists, Long Acting Beta-Agonists and Inhaled Corticosteroids) within 365 days of each other, within the two years before the index date. From the expected most specific to most sensitive, the eight algorithms were constructed as follows:

- 1. Specific asthma Read code + evidence of reversibility testing (spirometry, variable Peak Expiratory Flow Rate or trial of treatment) + respiratory medications
- 2. Specific asthma code + evidence of reversibility testing
- 3. Specific asthma code + respiratory medications
- 4. Specific asthma code only
- 5. Non-specific asthma code + evidence of reversibility testing + respiratory medications
- 6. Asthma Symptoms (wheeze, breathlessness, chest tightness, cough) + evidence of reversibility testing + respiratory medications
- 7. Asthma Symptoms + evidence of reversibility testing
- 8. Asthma Symptoms + respiratory medications

For peer review only - http://bmjopen.bmj.com/site/about/guidelines.xhtml

Primary outcome

The primary outcome was confirmation of a diagnosis of asthma in each of the eight predefined algorithms. The gold standard for the diagnosis of asthma was the adjudicated asthma status agreed by the two study physicians, a respiratory physician and a GP who reviewed all questionnaires and evidence from the patient's GP independently. The reviewers were blinded to the code lists/algorithm. Where opinion differed, the cases were discussed and agreement was reached by consensus. The reviewing physicians did not know with which algorithm a person was selected.

Statistical analysis

The Positive Predictive Value (PPV) was calculated using the proportion of cases identified by each algorithm that were confirmed as actual cases by the study physicians through a review of the questionnaire and supporting evidence. All analyses were conducted using Stata 14.0.

A patient could contribute only to a single algorithm for the main analysis. In the post hoc analysis, individuals could be placed into multiple algorithms where possible to reduce the confidence intervals. The PPV in this analysis was calculated for all individuals who had a specific asthma code compared to those with a specific asthma code and additional information. We also performed a sensitivity analysis to check whether the age and sex for

For peer review only - http://bmjopen.bmj.com/site/about/guidelines.xhtml

patients whose questionnaire was returned was similar to the age and sex of those patients whose questionnaire was not sent out or were there was no response.

Sample size calculation

As there were 116 patients that could not be evaluated, precision was expected to be slightly lower than in the original sample size calculations. However, a percentage difference in PPV of 0.13 is demonstrable with a sample size of 60 per algorithm (assuming PPV=0.85, alpha=0.05 and power=0.8).

RESULTS

A total of 800 potential asthma cases were selected for validation, of which 116 cases had migrated out of the database at the time the questionnaires were sent. Of the remaining 684 cases, there were 494 returned questionnaires. Nineteen of the returned questionnaires were considered invalid. Thus, 475 valid questionnaires were received, which yielded a response rate of 69.4% (475/684) using the practices that could have answered as denominator, as shown in figure 1. The time interval between the mailing of questionnaires and the review by the study physicians varied, but none of these time intervals was greater than 8 months.

The baseline characteristics of the 475 patients with valid returned questionnaires are shown in table 1. The study populations were mostly middle aged, never smokers and female. There

For peer review only - http://bmjopen.bmj.com/site/about/guidelines.xhtml

were 97 individuals whose smoking status was not filled in on the questionnaire. Differences in the majority of characteristics were seen among most algorithms.

The positive predictive values of the eight algorithms are displayed in table 2.

The PPV's of algorithms containing specific or non-specific asthma codes in algorithms 1-5 (ranging from 83.3% to 90.7%) are markedly higher than the PPV's of the algorithms based on asthma symptoms (ranging from 43.1% to 57.8%). The combination of a specific code and asthma medication prescription and/or evidence of reversibility testing (PPV varies from 83.3% to 86.8%) did not considerably increase the PPV compared to a specific asthma code alone (PPV 86.4%). The highest PPV was found in the fifth algorithm combining a non-specific asthma code with evidence of reversibility testing and asthma medication use. However, the total number of patients identifiable with this algorithm (n=33,280) was less than one fifth of those identifiable by the fourth algorithm consisting of a specific asthma code alone (n=188,133) in the chosen time period. We have not examined the validity of a non-specific asthma code alone.

A post-hoc analysis was performed where individuals were placed in every algorithm they qualified for. In this analysis, we found that the use of additional information on evidence of reversibility testing or medication in an algorithm with a specific asthma code again did not meaningfully increase the PPV. The PPV for all individuals who had a specific asthma code

For peer review only - http://bmjopen.bmj.com/site/about/guidelines.xhtml

and information on reversibility testing or medication was 86.7% (95% CI 83.3% to 90.1%), and the PPV for individuals with only a specific asthma code was 86.4% (95% CI 83.0% to 89.7%).

When validating the record of possible asthma with a gold standard based on the study physicians' view of extra evidence provided by the GP, the PPV slightly improved across all algorithms. Figure 2 demonstrates the PPV of the different algorithms as diagnosed by the patient's own GP and the study physicians (overall κ=0.81).

There was no considerable difference in age or sex between patients whose questionnaire was returned and patients whose questionnaire was not sent out (age: p=0.74, sex: p=0.73) or were there was no response (age p=0.50, sex p=0.13) using χ^2 tests.

For peer review only

For peer review only - http://bmjopen.bmj.com/site/about/guidelines.xhtml

DISCUSSION

We tested the accuracy of eight algorithms to identify asthma within CPRD using a gold standard constructed using a consensus of the two study physicians. The algorithm with the highest PPV consisted of a combination for nonspecific asthma codes, evidence of reversibility testing and multiple asthma prescriptions within one year (PPV 90.7, 95% CI 82.8 to 0.98.7) followed by a combination for specific asthma codes, evidence of reversibility testing and multiple asthma prescriptions within one year. The confidence interval of this PPV overlaps with the confidence intervals of each of the PPV's of the first four algorithms based on specific asthma codes, so the difference might be due to chance alone. The algorithm with the lowest PPV consisted of asthma symptoms and evidence of reversibility testing (PPV 0.43, 95% CI 0.30-0.55). The results of this validation study suggest that the clinical code based algorithms that use asthma codes to identify asthma cases have high PPVs (between 0.84 and 0.91). In this dataset, a specific asthma code algorithm alone appears sufficient to identify current asthma patients from CPRD. As the additional requirements of medication use and evidence of reversibility testing do not appear to significantly increase the PPV, the total number of individuals who can potentially be included in a study increases from 33,280 to 188,133 in the chosen time period (1st of December 2013 to 30th of November 2015). The total identifiable population of people living with asthma is thus much larger when only using a specific asthma code for identification.

For peer review only - http://bmjopen.bmj.com/site/about/guidelines.xhtml

Comparison with previous studies

Validity of asthma codes in electronic health records can be assessed by comparison to three different sets of gold standard: comparison to an external database, questionnaire and manual review by a clinician. This validation study uses questionnaires and manual review. Our gold standard consisted of the agreement of the study respiratory physician and study GP, both of whom were experienced with CPRD.

Previous studies which validated asthma in other EHR databases used manual review by clinicians to validate asthma in EHR and all reported at least one algorithm with a PPV above 85% (18-23). In contrast with this study, the best results in previous studies arose when combining diagnostic data and prescription data.

Strengths of this study

This study has several strengths. First, we were able to investigate the accuracy of eight pre-defined different algorithms and how they perform in identification of people with asthma in CPRD, as well as the accuracy of the actual GP diagnosis of asthma using additional information provided. Second, we included supporting information such as outpatient referral letters, other emergency department discharge letters, airflow measurements and radiography records. Finally, we validated asthma diagnoses found in CPRD, which is a primary care

For peer review only - http://bmjopen.bmj.com/site/about/guidelines.xhtml

database that is extensively used for studying different health outcomes in epidemiological research. This primary care database provides health and medication history of millions of patients. A validated definition in CPRD of asthma allows for informed health-care service planning by increasing the reliability of evidence generated from observational studies.

Limitations of this study

This study has limitations to consider. The gold standard consisting of a GP questionnaire and review by study physicians is not absolute, even if we mitigated this with additional information from secondary care. A GP can look in the electronic health record to see if a specific diagnosis has been recorded for a specific patient when asked. This may lead to an overestimation of the PPV, but there is no suitable practical alternative. Ideally, airflow measurements and reversibility testing on each potential patient would form the optimal gold standard, but this would not be feasible in this setting due to cost. The overall number of questionnaires sent out (684) was less than requested (800) as some patients and practices were no longer part of CPRD and could not be contacted. However, the precision of PPV estimates was not substantially reduced.

Although practices contributing to CPRD are a sample of all practices in the UK, they are considered representative of the UK population with few patients opting out of contributing data, and is therefore unlikely to bias the results (11).

For peer review only - http://bmjopen.bmj.com/site/about/guidelines.xhtml

GP's of patients with complicated medical histories could be less likely to return the questionnaire. The GP's were remunerated for their participation however, which is likely to have reduced the chance of this happening. Within the returned questionnaires, the amount of missing data was low, which suggests reasonable data quality. In addition, only living patients were assessed, as GP's no longer have access to the patient records after death. This excludes the records of the deceased patients and could result in survival bias. Patients had to be alive to be included, but it is unlikely that coding would differ between living and deceased individuals. If deceased people had died of asthma, the PPV in this study would be underestimated. Our findings are likely to be generalizable to other UK primary care databases using Read coding, but these would ideally still require validation. Databases using other coding systems may need to validate different algorithms to identify asthma, which might limit the generalisability of our findings. Another limitation is that we were not able to assess the Negative Predictive Value (NPV) of asthma diagnoses in CPRD because we evaluated only patients belonging to one of the eight algorithms. We could not calculate the specificity or sensitivity as we had preselected our population of possible asthma cases. We also assumed the validity of asthma diagnoses would not be different between common and less frequent Read codes and the quality of recording would also be comparable for pragmatic reasons. However, the less commonly used codes will by definition identify a

For peer review only - http://bmjopen.bmj.com/site/about/guidelines.xhtml

smaller proportion of all asthma patients, so the validity we report will apply to the majority of patients.

CONCLUSION

We have successfully estimated the PPV of several different algorithms to identify people with asthma in CPRD. The PPVs for specific asthma Read codes alone and non-specific ones in a combination with additional evidence were all greater than 0.84. A specific asthma code algorithm alone appears to be the most practical approach to identify patients with asthma in CPRD (PPV=0.86; 95% CI 0.77-0.95). Diagnoses were confirmed in a high proportion of patients with specific asthma codes, suggesting that epidemiological asthma research conducted using CPRD data can be conducted with reasonably high validity.

Dissemination and ethics

The protocol for this research was approved by the Independent Scientific Advisory Committee (ISAC) for MHRA Database Research (protocol number15_257) and the approved protocol was made available to the journal and reviewers during peer review. Generic ethical approval for observational research using the CPRD with approval from ISAC has been granted by a Health Research Authority (HRA) Research Ethics Committee (East Midlands – Derby, REC reference number 05/MRE04/87).

For peer review only - http://bmjopen.bmj.com/site/about/guidelines.xhtml

The results will be submitted for publication and will be disseminated through research conferences and peer reviewed journals.

Funding statement

This work was supported by GlaxoSmithKline (GSK), through a PhD scholarship for FN with grant number EPNCZF5310. The publishing of this study was supported by the Wellcome Trust: grant number 098504/Z/12/Z.

Competing interests

FN is funded by a GSK scholarship during his PhD program. IJD is funded by an unrestricted grant from, has consulted for, and holds stock in GlaxoSmithKline. HM is an employee of GSK R&D and own shares of GSK Plc. JKQ reports grants from MRC, BLF, Wellcome Trust and has received research funds from GSK, AZ, Quintiles IMS, in addition to personal fees from AZ, Chiesi, BI .

Contributors

For peer review only - http://bmjopen.bmj.com/site/about/guidelines.xhtml

JKQ, IJD, LS and HM were responsible for developing the research question and have advised on the data collection and search strategies. FN summarised and analysed the questionnaires and drafted the manuscript. JKQ and DM reviewed the questionnaires and constructed the gold standard for asthma validation. JKQ is responsible for study management and coordination. All authors have read, commented on and approved the final manuscript.

Data sharing statement

Study data will be available on request to FN once the research team has completed preplanned analyses.

Figure legend

Figure 1: Study population

Figure 2: PPV as diagnosed by the patient's own GP, and agreement between the study physicians

Table legend

Table 1: Characteristics of the 475 patients included in the final study analysis

Table 2: The Positive Predictive Value (PPV) and proportion of patients diagnosed with Chronic Obstructive Pulmonary Disease (COPD) within each algorithm

Appendices

For peer review only - http://bmjopen.bmj.com/site/about/guidelines.xhtml

1. Appendix 1: CPRD Read codes indicating asthma

2. Appendix 2: General Practitioner questionnaire

3. Appendix 3: ISAC study protocol

For peer review only

For peer review only - http://bmjopen.bmj.com/site/about/guidelines.xhtml

References

1. Global, regional, and national age-sex specific all-cause and cause-specific mortality for 240 causes of death, 1990-2013: a systematic analysis for the Global Burden of Disease Study 2013. Lancet. 2015;385(9963):117-71.

2. James DR, Lyttle MD. British guideline on the management of asthma: SIGN Clinical Guideline 141, 2014. Arch Dis Child Educ Pract Ed. 2016;101(6):319-22.

3. Haldar P, Pavord ID, Shaw DE, Berry MA, Thomas M, Brightling CE, et al. Cluster analysis and clinical asthma phenotypes. Am J Respir Crit Care Med. 2008;178(3):218-24.

4. Langan SM, Benchimol EI, Guttmann A, Moher D, Petersen I, Smeeth L, et al. Setting the RECORD straight: developing a guideline for the REporting of studies Conducted using Observational Routinely collected Data. Clin Epidemiol. 2013;5:29-31.

5. Denney MJ, Long DM, Armistead MG, Anderson JL, Conway BN. Validating the extract, transform, load process used to populate a large clinical research database. Int J Med Inform. 2016;94:271-4.

6. Lo Re V, 3rd, Haynes K, Forde KA, Localio AR, Schinnar R, Lewis JD. Validity of The Health Improvement Network (THIN) for epidemiologic studies of hepatitis C virus infection. Pharmacoepidemiol Drug Saf. 2009;18(9):807-14.

7. Ehrenstein V, Petersen I, Smeeth L, Jick SS, Benchimol EI, Ludvigsson JF, et al. Helping everyone do better: a call for validation studies of routinely recorded health data. Clin Epidemiol. 2016;8:49-51.

8. ENCePP. ENCePP Guide on Methodological Standards in Pharmacoepidemiology: ENCePP; 2017 [cited 2017 31/03/2017]. Available from: http://www.encepp.eu/standards_and_guidances/methodologicalGuide3_2.shtml.

9. Bousquet J, Mantzouranis E, Cruz AA, Ait-Khaled N, Baena-Cagnani CE, Bleecker ER, et al. Uniform definition of asthma severity, control, and exacerbations: document presented for the World Health Organization Consultation on Severe Asthma. J Allergy Clin Immunol. 2010;126(5):926-38.

10. Sin DD, Miravitlles M, Mannino DM, Soriano JB, Price D, Celli BR, et al. What is asthma-COPD overlap syndrome? Towards a consensus definition from a round table discussion. Eur Respir J. 2016;48(3):664-73.

11. Herrett E, Gallagher AM, Bhaskaran K, Forbes H, Mathur R, van Staa T, et al. Data Resource Profile: Clinical Practice Research Datalink (CPRD). Int J Epidemiol. 2015;44(3):827-36.

12. Williams T, van Staa T, Puri S, Eaton S. Recent advances in the utility and use of the

For peer review only

For peer review only - http://bmjopen.bmj.com/site/about/guidelines.xhtml

General Practice Research Database as an example of a UK Primary Care Data resource. Ther Adv Drug Saf. 2012;3(2):89-99.

13. Herrett E, Thomas SL, Schoonen WM, Smeeth L, Hall AJ. Validation and validity of diagnoses in the General Practice Research Database: a systematic review. Br J Clin Pharmacol. 2010;69(1):4-14.

14. Thomas KH, Davies N, Metcalfe C, Windmeijer F, Martin RM, Gunnell D. Validation of suicide and self-harm records in the Clinical Practice Research Datalink. Br J Clin Pharmacol. 2013;76(1):145-57.

15. Rothnie KJ, Müllerová H, Hurst JR, Smeeth L, Davis K, Thomas SL, et al. Validation of the Recording of Acute Exacerbations of COPD in UK Primary Care Electronic Healthcare Records. PLoS One. 2016;11(3).

16. Quint JK, Müllerova H, DiSantostefano RL, Forbes H, Eaton S, Hurst JR, et al. Validation of chronic obstructive pulmonary disease recording in the Clinical Practice Research Datalink (CPRD-GOLD). BMJ Open. 2014;4(7).

17. Chew-Graham CA, Hunter C, Langer S, Stenhoff A, Drinkwater J, Guthrie EA, et al. How QOF is shaping primary care review consultations: a longitudinal qualitative study. BMC Fam Pract. 2013;14:103.

18. Xi N, Wallace R, Agarwal G, Chan D, Gershon A, Gupta S. Identifying patients with asthma in primary care electronic medical record systems Chart analysis-based electronic algorithm validation study. Canadian Family Physician. 2015;61(10):e474-83.

19. Kozyrskyj AL, HayGlass KT, Sandford AJ, Pare PD, Chan-Yeung M, Becker AB. A novel study design to investigate the early-life origins of asthma in children (SAGE study). Allergy. 2009;64(8):1185-93.

20. Pacheco JA, Avila PC, Thompson JA, Law M, Quraishi JA, Greiman AK, et al. A highly specific algorithm for identifying asthma cases and controls for genome-wide association studies. AMIA Annual Symposium Proceedings/AMIA Symposium. 2009;2009:497-501.

21. Vollmer WM, O'Connor EA, Heumann M, Frazier EA, Breen V, Villnave J, et al. Searching multiple clinical information systems for longer time periods found more prevalent cases of asthma. Journal of Clinical Epidemiology. 2004;57(4):392-7.

22. Donahue JG, Weiss ST, Goetsch MA, Livingston JM, Greineder DK, Platt R. Assessment of asthma using automated and full-text medical records. Journal of Asthma. 1997;34(4):273-81.

23. Premaratne UN, Marks GB, Austin EJ, Burney PGJ. A reliable method to retrieve Accident and Emergency data stored on a free-text basis. Respiratory Medicine. 1997;91(2):61-6
.

For peer review only - http://bmjopen.bmj.com/site/about/guidelines.xhtml

Algorithm	1. specific asthma code + reversibility testing + medication	2. specific asthma code + reversibility testing	3. specific asthma code + medication	4. specific asthma code	5. non-specific asthma code + reversibility testing + medication	6. symptoms + reversibility testing + medication	7. symptoms + reversibility testing	8. symptoms + medication	Total
**Individuals, N (%)**	68 (100)	57 (100)	60 (100)	59 (100)	54 (100)	55 (100)	58 (100)	64 (100)	475
**Asthma diagnosis by patient's GP**	56 (82.4)	49 (86)	48 (80)	51 (86.4)	48 (88.9)	29 (52.7)	23 (39.7)	38 (59.4)	342
**Confirmation by respiratory physician before study start**	55 (80.9)	29 (50.9)	38 (63.3)	45 (76.3)	34 (63)	23 (41.8)	25 (43.1)	36 (56.3)	285
**Evidence of reversible airway obstruction**	47 (69.1)	37 (64.9)	32 (53.3)	32 (54.2)	31 (57.4)	26 (47.3)	19 (32.8)	26 (40.6)	250
**Mean age**	52.3	51.4	47	41.9	45	60.9	61.3	52.1	
**Mean age: (95% CI)**	(47.4 to 57.2)	(46.2 to 56.7)	(41.4 to 52.6)	(36.1 to 47.6)	(38.7 to 51.3)	(55.3 to 66.4)	(57.1 to 65.5)	(45.4 to 58.7)	
**< 18 years old (%)**	7.35	7.02	15.25	18.64	16.67	7.27	1.72	20.31	11.81
**Sex: male**	31 (45.6)	17 (29.8)	16 (26.7)	23 (39)	26 (48.1)	28 (50.9)	24 (41.4)	31 (48.4)	196
**Current smoker***	11 (16.2)	10 (17.5)	10 (16.7)	5 (8.5)	4 (7.4)	5 (9.1)	8 (13.8)	4 (6.3)	57
**Ex-smoker***	16 (23.5)	14 (24.6)	17 (28.3)	16 (27.1)	15 (27.8)	11 (20)	10 (17.2)	12 (18.8)	111
**Never smoker***	35 (51.5)	26 (45.6)	25 (41.7)	36 (61.0)	32 (59.3)	18 (32.7)	11 (19.0)	27 (42.2)	210
**Individuals with supporting info**	23 (33.8)	21 (36.8)	22 (36.7)	14 (23.7)	14 (25.9)	17 (30.9)	14 (24.1)	22 (34.4)	147

** as stated by patient's GP on the study questionnaire*

Table 1: Characteristics of the 475 patients included in the final study analysis

For peer review only

Algorithm	Eligible population	Questionnaires sent out	Valid returned questionnaires (N,%)	Confirmed asthma cases	PPV (95% CI)
1. specific asthma code + reversibility testing + medication	36,516	92	68 (60)	61	86.8 (78.5 to 95.0)
2. specific asthma code + reversibility testing	38,796	90	57 (63.3)	51	86.0 (76.7 to 95.3)
3. specific asthma code + medication	169,574	89	60 (67.4)	51	83.3 (73.6 to 93.0)
4. specific asthma code	188,133	84	59 (70.2)	51	86.4 (77.4 to 95.4)
5. non-specific asthma code + reversibility testing + medication	33,280	78	54 (69.2)	49	90.7 (82.8 to 98.7)
6. symptoms + reversibility testing + medication	53,117	87	55 (63.2)	32	56.4 (42.8 to 69.9)
7. symptoms + reversibility testing	66,477	88	58 (65.9)	26	43.1 (30.0 to 56.2)
8. symptoms + medication	190,753	78	64 (82.1)	38	57.8 (45.4 to 70.2)

Medication use was defined as two prescriptions within 365 days. Evidence of reversibility testing does not hold information on the outcome of these tests.

Table 1: The Positive Predictive Value (PPV) and proportion of patients diagnosed with Chronic Obstructive Pulmonary Disease (COPD) within each algorithm

For peer review only

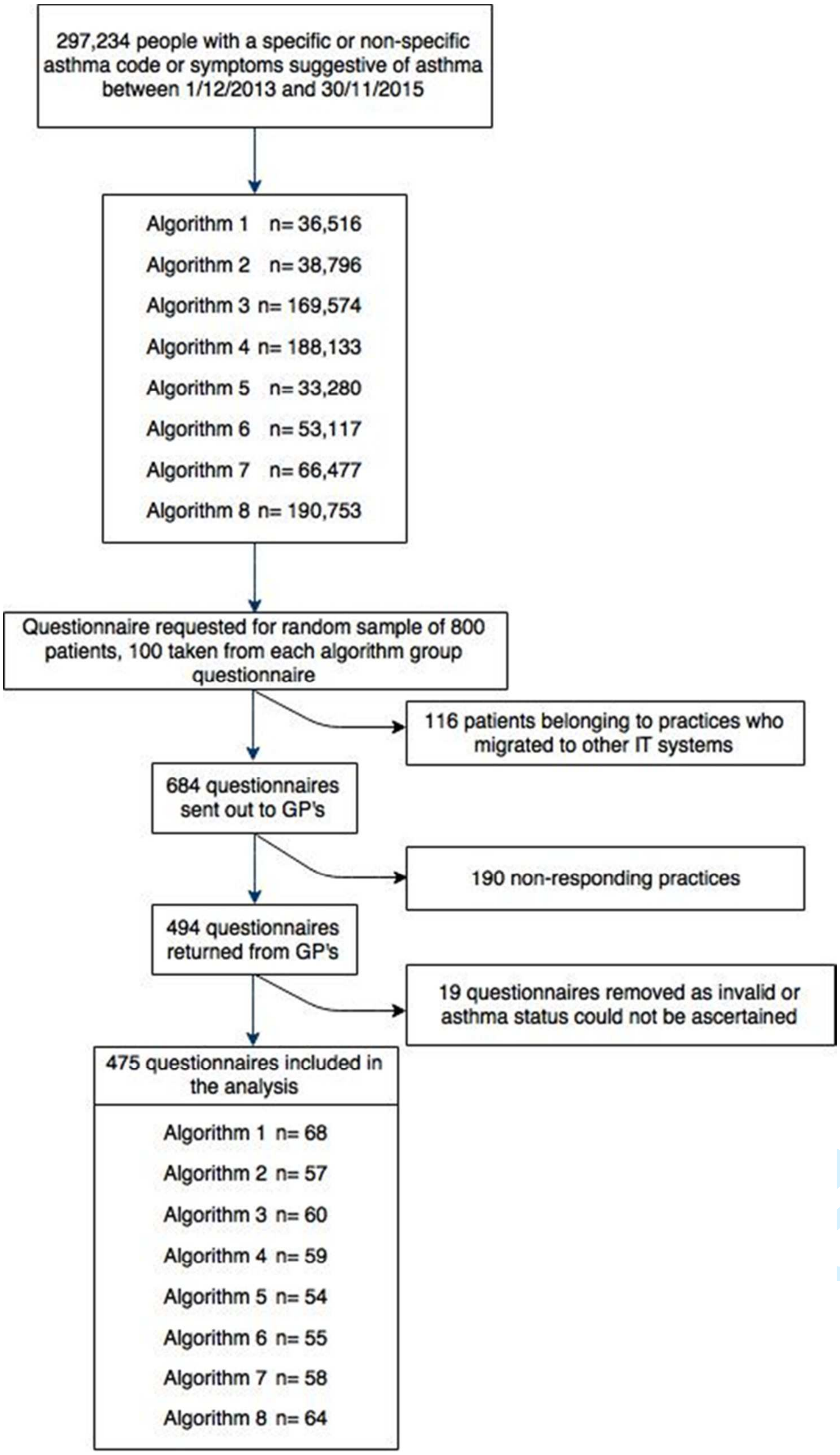

Figure 1: Study population

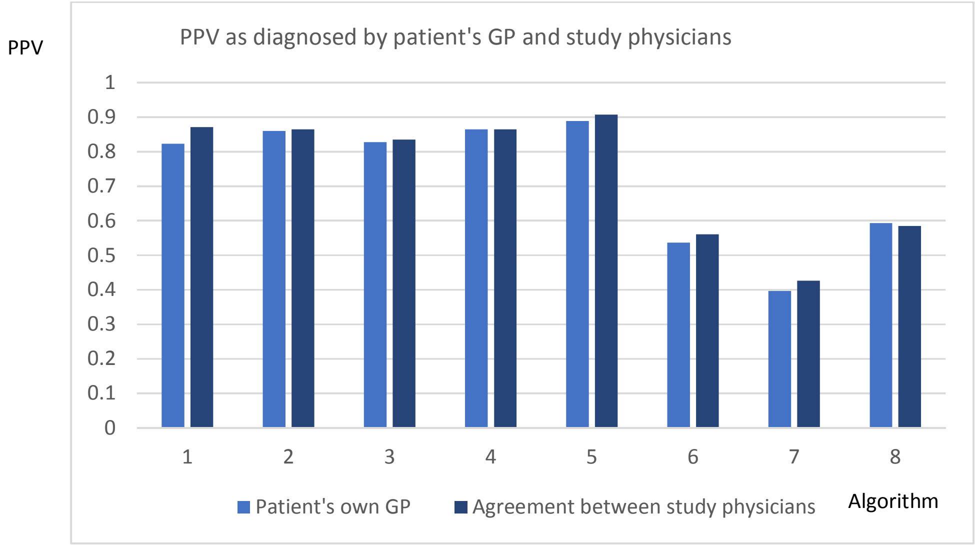

Figure 1: PPV as diagnosed by the patient's own GP, and agreement between the study physicians

For peer review only

Appendix 1: CPRD medcodes indicating asthma

A) Specific asthma codes

medcode	readterm
78	asthma
81	asthma monitoring
185	acute exacerbation of asthma
232	asthma attack
233	severe asthma attack
1555	bronchial asthma
2290	allergic asthma
3018	mild asthma
3366	severe asthma
3458	occasional asthma
3665	late onset asthma
4442	asthma unspecified
4606	exercise induced asthma
4892	status asthmaticus nos
5267	intrinsic asthma
5627	hay fever with asthma
5798	chronic asthmatic bronchitis
5867	exercise induced asthma
6707	extrinsic asthma with asthma attack
7058	emergency admission, asthma
7146	extrinsic (atopic) asthma
7191	asthma limiting activities
7378	asthma management plan given
7416	asthma disturbing sleep
7731	pollen asthma
8335	asthma attack nos
8355	asthma monitored
9018	number of asthma exacerbations in past year
9552	change in asthma management plan
9663	step up change in asthma management plan
10043	asthma annual review
10274	asthma medication review
10487	asthma - currently active
11370	asthma confirmed
12987	late-onset asthma
13064	asthma severity
13065	moderate asthma
13175	asthma disturbs sleep frequently
13176	asthma follow-up

For peer review only

14777	extrinsic asthma without status asthmaticus
15248	hay fever with asthma
16070	asthma nos
16667	asthma control step 2
16785	asthma control step 1
18223	step down change in asthma management plan
18224	asthma control step 3
18323	intrinsic asthma with asthma attack
19167	asthma monitoring by nurse
19519	asthma treatment compliance unsatisfactory
19520	asthma treatment compliance satisfactory
20860	asthma control step 5
20886	asthma control step 4
21232	allergic asthma nec
22752	occupational asthma
24479	emergency asthma admission since last appointment
24506	further asthma - drug prevent.
24884	asthma causes daytime symptoms 1 to 2 times per week
25181	asthma restricts exercise
25791	asthma clinical management plan
26501	asthma never causes daytime symptoms
26503	asthma causes daytime symptoms most days
26504	asthma never restricts exercise
26506	asthma severely restricts exercise
26861	asthma sometimes restricts exercise
27926	extrinsic asthma with status asthmaticus
29325	intrinsic asthma without status asthmaticus
30458	asthma monitoring by doctor
30815	asthma causing night waking
31167	asthma night-time symptoms
31225	asthma causes daytime symptoms 1 to 2 times per month
38143	asthma never disturbs sleep
38144	asthma limits walking up hills or stairs
38145	asthma limits walking on the flat
38146	asthma disturbs sleep weekly
39478	wood asthma
39570	asthma causes night symptoms 1 to 2 times per month
40823	brittle asthma
41017	aspirin induced asthma
41020	absent from work or school due to asthma
42824	asthma daytime symptoms
45073	intrinsic asthma nos

For peer review only

45782	extrinsic asthma nos
46529	attends asthma monitoring
47337	asthma accident and emergency attendance since last visit
47684	detergent asthma
58196	intrinsic asthma with status asthmaticus
73522	work aggravated asthma
93353	sequoiosis (red-cedar asthma)
93736	royal college of physicians asthma assessment
98185	asthma control test
99793	patient has a written asthma personal action plan
100107	health education - asthma self management
100397	asthma control questionnaire
100509	under care of asthma specialist nurse
100740	health education - structured asthma discussion
102170	asthma review using roy colleg of physicians three questions
102209	mini asthma quality of life questionnaire
102301	asthma trigger - seasonal
102341	asthma trigger - pollen
102395	asthma causes symptoms most nights
102400	asthma causes night time symptoms 1 to 2 times per week
102449	asthma trigger - respiratory infection
102713	asthma limits activities 1 to 2 times per month
102871	asthma trigger - exercise
102888	asthma limits activities 1 to 2 times per week
102952	asthma trigger - warm air
103318	health education - structured patient focused asthma discuss
103321	asthma trigger - animals
103612	asthma never causes night symptoms
103631	royal college physician asthma assessment 3 question score
103813	asthma trigger - cold air
103944	asthma trigger - airborne dust
103945	asthma trigger - damp
103952	asthma trigger - emotion
103955	asthma trigger - tobacco smoke
103998	asthma limits activities most days
105420	asthma self-management plan review
105674	asthma self-management plan agreed
106805	chronic asthma with fixed airflow obstruction
107167	number days absent from school due to asthma in past 6 month

B) Non-specific asthma codes

medcode	readterm

For peer review only

719	h/o: asthma
1208	childhood asthma
5138	patient in asthma study
5515	seen in asthma clinic
7229	asthma prophylactic medication used
11022	asthma trigger
11387	refuses asthma monitoring
11673	excepted from asthma quality indicators: patient unsuitable
11695	excepted from asthma quality indicators: informed dissent
13066	asthma - currently dormant
13173	asthma not disturbing sleep
13174	asthma not limiting activities
16655	asthma monitoring admin.
18141	asthma monitoring due
18692	exception reporting: asthma quality indicators
18763	referral to asthma clinic
19539	asthma monitoring check done
20422	asthma clinic administration
25705	asthma monitor 3rd letter
25706	asthma monitor 2nd letter
25707	asthma monitor 1st letter
25796	mixed asthma
26496	health education - asthma
29645	asthma control step 0
30308	dna - did not attend asthma clinic
30382	asthma monitoring admin.nos
31135	asthma monitor phone invite
35927	asthma leaflet given
37943	asthma monitor verbal invite
41554	asthma monitor offer default
43770	asthma society member
92109	asthma outreach clinic

For peer review only

Study into asthma: questionnaire for £55, further information for £55

The London School of Hygiene and Tropical Medicine is conducting a study to investigate the best way to identify asthma within the Clinical Practice Research Datalink (CPRD). We have developed several methods for identifying asthma in the database, and we would like to obtain some information on the current asthma status of the patient from GPs so that we can decide which method is the most suitable.

We would be very grateful if you could supply us with the following information.

A. Do you agree this patient has a current diagnosis of asthma?

- ☐ Yes: Proceed to question B
- ☐ No: Proceed to question C
- ☐ Uncertain: Proceed to question B

If you answered yes or uncertain to question A:

B1. Has the diagnosis been made or confirmed by a respiratory physician?

- ☐ Yes
- ☐ No

B2. Does this patient have evidence of reversible airway obstruction?

- ☐ Yes
- ☐ No

If yes: **Was this based on;**

- ☐ Spirometry reversibility with a bronchodilator
- ☐ Trial of treatment with oral or inhaled corticosteroids and diurnal variation on a peak flow diary

B3. In what year was the asthma first diagnosed?

B4. Were any other factors taken into consideration in making the diagnosis?

Yes **No**

For peer review only

a. History of atopic disorder	☐	☐
b. Family history of asthma and/or atopic disorder	☐	☐
c. Widespread wheeze heard on auscultation of the chest	☐	☐
d. Otherwise unexplained low FEV (Forced Expiratory Volume) or PEF (Peak Expiratory Flow) on spirometry	☐	☐
e. Otherwise unexplained variability in PEFR (Peak Expiratory Flow Rate) on spirometry	☐	☐
f. Otherwise unexplained peripheral blood eosinophilia	☐	☐
g. FeNO (Fractional exhaled Nitric Oxide) measurement	☐	☐
h. Other (please name)		

B5. Based on the QOF (Quality and Outcomes Framework) indicators:

	Yes	No
a. Does the patient have any difficulty sleeping because of asthma symptoms, including cough	☐	☐
b. Does the patient have the usual asthma symptoms during the day (cough, wheeze, chest tightness of breathlessness)?	☐	☐
c. Does the asthma interfere with the patient's usual activities (housework, work, school, etc.)?	☐	☐

B6. What is the patient's smoking status?

☐ Current smoker

☐ Ex-smoker

☐ Never-smoker

B7. Does the patient have any other respiratory diseases? (Multiple responses possible)

☐ Chronic Obstructive Pulmonary Disease (COPD)

☐ Bronchiectasis

☐ Interstitial Lung Disease

☐ Other, please list:

☐ No

For peer review only

If you answered no to question A:

C. Do you think this patient has a history of asthma?

☐ Yes

☐ No

☐ Uncertain

For peer review only

For peer review only

ISAC APPLICATION FORM
PROTOCOLS FOR RESEARCH USING THE CLINICAL PRACTICE RESEARCH DATALINK (CPRD)

ISAC use only: Protocol Number Date submitted	....	**IMPORTANT** **If you have any queries, please contact ISAC Secretariat:** ISAC@cprd.com

Section A: The study

1. Study Title
Validation of the recording of asthma diagnosis in adult patients in the Clinical Practice Research Datalink

2. Has any part of this research proposal or a related proposal been previously submitted to ISAC?
Yes ☐ No ☒
If Yes, please provide previous protocol numbers:

3. Has this protocol been peer reviewed by another Committee? (e.g. grant award or ethics committee)
Yes ☒ No ☐
If Yes, please state the name of the reviewing Committee(s) and provide an outline of the review process and outcome: Internal review by GSK, PRF committee

4. Type of Study (please tick all the relevant boxes which apply)

Adverse Drug Reaction/Drug Safety ☐
Drug Effectiveness ☐
Health/Public Health Services Research ☒
Drug Utilisation ☐
Pharmacoeconomics ☐
Disease Epidemiology ☒
Methodological ☒
Post-authorisation Safety ☐
Other* ☐
*Please specify the type of study in the lay summary

5. This study is intended for (please tick all the relevant boxes which apply)**:**

Publication in peer reviewed journals ☒
Presentation at company/institutional meetings ☒
Other
Presentation at scientific conference ☒
Regulatory purposes ☐

Section B: The Investigators

6. Chief Investigator (full name, job title, organisation name & e-mail address for correspondence- see guidance notes for eligibility)
Jennifer Quint, Clinical Senior lecturer in Respiratory epidemiology, Imperial College London, j.quint@imperial.ac.uk
CV has been previously submitted to ISAC ☒ **CV number:** 042_15CEPSL
A new CV is being submitted with this protocol ☐
An updated CV is being submitted with this protocol ☐

7. Affiliation (full address)
Department of NCDE, LSHTM, Keppel Street, London WC1E 7HT

8. Corresponding Applicant
Francis Nissen, PhD researcher, LSHTM, francis.nissen@lshtm.ac.uk
Same as chief investigator ☐
CV has been previously submitted to ISAC ☒ **CV number:** 449_15S
A new CV is being submitted with this protocol ☐
An updated CV is being submitted with this protocol ☐

9. List of all investigators/collaborators (*please list the full names, affiliations and e-mail addresses* of all collaborators, other than the Chief Investigator*)

Other investigator: Ian Douglas, Senior lecturer, LSHTM, ian.douglas@lshtm.ac.uk
CV has been previously submitted to ISAC ☒ **CV number:** 157_15CESL
A new CV is being submitted with this protocol ☐
An updated CV is being submitted with this protocol ☐

Other investigator: Liam Smeeth, LSHTM, Liam.Smeeth@lshtm.ac.uk
CV has been previously submitted to ISAC ☒ **CV number:** 045_15CEPSL
A new CV is being submitted with this protocol ☐
An updated CV is being submitted with this protocol ☐

Other investigator: Hana Müllerova, GSK, hana.x.muellerova@gsk.com
CV has been previously submitted to ISAC ☒ **CV number:** 365_15E
A new CV is being submitted with this protocol ☐
An updated CV is being submitted with this protocol ☐

For peer review only - http://bmjopen.bmj.com/site/about/guidelines.xhtml
For peer review only

Other investigator: Daniel Morales, University of Dundee, d.r.z.morales@dundee.ac.uk
CV has been previously submitted to ISAC ☒ **CV number:** 450_15P
A new CV is being submitted with this protocol ☐
An updated CV is being submitted with this protocol ☐

Other investigator: Neil Pearce, LSHTM, Neil.Pearce@lshtm.ac.uk
CV has been previously submitted to ISAC ☒ **CV number:** 367_15CS
A new CV is being submitted with this protocol ☐
An updated CV is being submitted with this protocol ☐

[Please add more investigators as necessary] **Please note that your ISAC application form and protocol **must** be copied to all e-mail addresses listed above at the time of submission of your application to the ISAC mailbox. Failure to do so will result in delays in the processing of your application.*

10. Conflict of interest statement* (please provide a draft of the conflict (or competing) of interest (COI) statement that you intend to include in any publication which might result from this work)

All authors have completed the ICMJE uniform disclosure at www.icmje.org/coi_disclosure.pdf and declare:
FN has received a PhD scholarship from GSK
Dr Quint reports grants from MRC, GSK, BLF, Wellcome. Personal fees from AZ, GSK.
ID has consulted for and holds stock in GSK
**Please refer to the International Committee of Medical Journal Editors (ICMJE) for guidance on what constitutes a COI*

11. Experience/expertise available (please complete the following questions to indicate the experience/expertise available within the team of investigators/collaborators actively involved in the proposed research, including the analysis of data and interpretation of results

	Previous GPRD/CPRD Studies	Publications using GPRD/CPRD data
None	☐	☐
1-3	☐	☐
> 3	☒	☒

	Yes	No
**Is statistical expertise available within the research team?**<br>*If yes, please indicate the name(s) of the relevant investigator(s)*<br>Ian Douglas	☒	☐
**Is experience of handling large data sets (>1 million records) available within the research team?**<br><br>*If yes, please indicate the name(s) of the relevant investigator(s)*<br>Ian Douglas<br>Jennifer Quint<br>Liam Smeeth<br>Daniel Morales	☒	☐
**Is experience of practising in UK primary care available within the research team?**<br><br>*If yes, please indicate the name(s) of the relevant investigator(s)*<br>Liam Smeeth<br>Daniel Morales	☒	☐

12. References relating to your study

Please list up to 3 references (most relevant) relating to your proposed study:

Quint JK, Müllerova H, DiSantostefano RL, Forbes H, Eaton S, Hurst JR, Davis K, Smeeth L.: Validation of chronic obstructive pulmonary disease recording in the Clinical Practice Research Datalink (CPRD-GOLD). BMJ Open. 2014 Jul 23;4(7)

Cornish RP, Henderson J, Boyd AW, Granell R, Van Staa T, Macleod: Validating childhood asthma in an epidemiological study using linked electronic patient records. J. BMJ Open. 2014 Apr 23;4(4)

British Thoracic Society Scottish Intercollegiate Guidelines Network. British guideline on the management of

For peer review only - http://bmjopen.bmj.com/site/about/guidelines.xhtml

asthma. Thorax 2008;63(Suppl 4):iv1–121.

Section C: Access to the data

13. Financial Sponsor of study

Pharmaceutical Industry	☒ *Please specify:* GSK	Academia	☐ *Please specify:*
Government / NHS	☐ *Please specify:*	Charity	☐ *Please specify:*
Other	☐ *Please specify:*	None	☐

14. Type of Institution carrying out the analyses

Pharmaceutical Industry	☐ *Please specify:*	Academia	☒ *Please specify:* LSHTM
Government Department	☐ *Please specify:*	Research Service Provider	☐ *Please specify:*
NHS	☐ *Please specify:*	Other	☐ *Please specify:*

15. Data source

The sponsor has direct access to CPRD GOLD and will extract the relevant data* ☒

A data set will be supplied by CPRD** ☐

CPRD has been commissioned to extract the relevant data and to perform the analyses ☐
Other ☐ *Please specify:*

*If data sources other than CPRD GOLD are required, these will be supplied by CPRD
** Please note that datasets provided by CPRD are limited in size. Applicants should contact CPRD (KC@CPRD.com) if a dataset of >300,000 patients is required.

16. Primary care data (please specify which primary care data set(s) are required)
Vision only (Default for CPRD studies) ☒
EMIS® only* ☐
Both Vision and EMIS®* ☐

Note: Vision and EMIS are different clinical systems, Vision data has traditionally been used for CPRD, EMIS is currently undergoing beta-testing.
**Investigators requiring the use of EMIS data must discuss the study with a member of CPRD staff before submitting an ISAC application*
Please list below the name of the person/s at the CPRD with whom you have discussed your request for EMIS data:

Section D: Data linkage

17. Does this protocol also seek access to data held under the CPRD Data Linkage Scheme?

Yes* ☒ No ☐

If No, please move to section E.

**Investigators requiring linked data must discuss the study with a member of CPRD staff. It is important to be aware that linked data are not available for all patients in CPRD GOLD, the coverage periods for each data source may differ and charges may be applied. Please contact the CPRD Research Team on +44 (20) 3080 6383 or email kc@cprd.com to discuss your requirements before submitting your application.*
Please list below the name of the person/s at the CPRD with whom you have discussed your request:

Please note that as part of the ISAC review of linkages, the protocol may be shared - in confidence - with a representative of the requested linked data set(s) and summary details may be shared - in confidence - with the Confidentiality Advisory Group of the Health Research Authority.

For peer review only - http://bmjopen.bmj.com/site/about/guidelines.xhtml

18. Please select the source(s) of linked data being requested:

☐ ONS Mortality Data
☐ Inpatient Hospital Episode Statistics
☐ Outpatient Hospital Episode Statistics
☐ NCDR Cancer Registry Data*
☐ MINAP
☐ Mother Baby Link

☒ Index of Multiple Deprivation
☐ Townsend Score
☐ Other** *Please specify:*

We have discussed the data linkages with Rachael Williams, Research Statistician at CPRD.

Please note that applicants seeking access to cancer registry data must provide consent for publication of their study title and study institution on the UK Cancer Registry website. They must also complete a **Cancer Dataset Agreement Form (available from CPRD) and provide a **System level Security Policy** for each organisation involved in the study.*

*** If "Other" is specified, please name an individual in CPRD that this linage has been discussed with.*

19. Total number of linked datasets requested including CPRD GOLD: 1

20. Is linkage to a local dataset with <1 million patients being requested?

Yes* ☐ No ☒

** If yes, please provide further details:*

21. If you have requested linked data sets, please indicate whether the Chief Investigator or any of the collaborators listed in response to question 5 above, have access to any of the linked datasets in a patient identifiable form, or associated with a patient index.

Yes* ☐ No ☒

** If yes, please provide further details:*

22. Does this study involve linking to patient *identifiable* data from other sources?

Yes ☐ No ☒

Section E: Validation/verification

23. Does this protocol describe a purely observational study using CPRD data (this may include the review of anonymised free text)?

Yes* ☒ No** ☐

** Yes: If you will be using data obtained from the CPRD Group, this study does not require separate ethics approval from an NHS Research Ethics Committee.*
*** No: You may need to seek separate ethics approval from an NHS Research Ethics Committee for this study. The ISAC will provide advice on whether this may be needed.*

24. Does this study require anonymised free text?

Yes* ☐ No ☒

**Please note that work involving free text can only be performed on the July 2013 CPRD GOLD database build or earlier versions. CPRD can provide further advice on the use of anonymised free text.*

25. Does this protocol involve requesting any additional information from GPs?

Yes* ☒ No ☐

* *Please indicate what will be required:*

Completion of questionnaires by the GP[Ψ]	Yes ☒	No ☐
Provision of anonymised records (e.g. hospital discharge summaries)	Yes ☒	No ☐
Other (please describe)		

[Ψ] Any questionnaire for completion by GPs or other health care professional must be approved by ISAC before circulation for completion.

For peer review only - http://bmjopen.bmj.com/site/about/guidelines.xhtml
For peer review only

**26. Does this study require contact with patients in order for them to complete a questionnaire?**<br><br>Yes* ☐ No ☒<br><br>**Please note that any questionnaire for completion by patients must be approved by ISAC before circulation for completion.*
**27. Does this study require contact with patients in order to collect a sample?**<br><br>Yes* ☐ No ☒<br><br>** Please state what will be collected:*
**Section F: Signatures**
**28. Signature from the Chief Investigator**<br><br>I confirm that the above information is to the best of my knowledge accurate, and I have read and understood the guidance to applicants.<br><br>Name: Jennifer Quint Date: 08/12/2015 E. signature (type name): Jennifer Quint

For peer review only

For peer review only - http://bmjopen.bmj.com/site/about/guidelines.xhtml

Protocol Section

<u>The following headings **must** be used to form the basis of the protocol. Pages should be numbered. All abbreviations must be defined on first use.</u>

A. Lay Summary (Max. 200 words)

This study will investigate the recording of the diagnosis of asthma in the primary care medical records database called Clinical Practice Research Datalink (CPRD GOLD). This will be done by the collection of information provided by general practitioners through a questionnaire. This information will then be examined by two independent expert physicians, giving a reliable diagnosis to be compared with the recording of asthma within the CPRD database. The diagnosis of asthma is mostly based on a characteristic pattern of symptoms and the absence of another diagnosis. Because of this, asthma is not as well defined as some other diseases, and the clinical diagnosis might be less accurate. The study to be undertaken could help establish the best strategy to identify individuals with asthma within the CPRD. This would inform the definitions and patient selection for further observational and potentially pragmatic intervention studies in CPRD and other primary care data sources.

B. Technical Summary (Max. 200 words)

The overall aim of this study is to determine the positive predictive value (PPV) of different algorithms using asthma diagnostic Read codes within the CPRD GOLD, i.e., a proportion of true positives among those assumed to have been diagnosed with asthma. In order to achieve this we will construct a retrospective cohort of asthma patients and compare database information (CPRD GOLD and the Multiple Deprivation Index) with information gathered by a questionnaire filled in by general practitioners and review of any supporting information sent. A review of these questionnaires by two independent expert physicians will be considered as the gold standard to assess the PPV of an asthma recording using specific algorithms in CPRD GOLD.

C. Objectives, Specific Aims and Rationale

(i) *Aim:*
To assess strategies to identify asthma patients of adults in United Kingdom electronic primary care records.

(ii) *Objectives:*
To determine the PPV of the recording of asthma diagnosis of adults within the CPRD GOLD database.

(iii) *Rationale:*
We will measure the level of accuracy, using the PPV, of an asthma diagnosis recording in the CPRD database employing a gold standard comprised of the review of general practitioners questionnaires by two independent experts. By doing so, we will be able to assess how reliable an asthma diagnosis is in electronic primary care records.

For peer review only - http://bmjopen.bmj.com/site/about/guidelines.xhtml

D. Background

Asthma is difficult to assess in health-care database epidemiological studies as the diagnostic criteria are based on non-specific respiratory symptoms and variable expiratory airflow limitation which are often not recorded in electronic medical records (1). According to the current estimates of the Global Burden of Disease Study 2013, 334 million people worldwide have asthma. 8.6% of young adults (aged 18-45) experience asthma symptoms and 4.5% of young adults worldwide have been diagnosed with asthma and/or are taking treatment for asthma (2). In the UK, 5.4 million people are currently receiving treatment for asthma, of whom 4.3 million are adults (3).

The British guideline on the management of asthma states that the diagnosis in adults is based on the recognition of a characteristic pattern of symptoms and signs and the absence of an alternative explanation. Based on clinical features that either increase or decrease the probability of asthma, patients are categorized in the “low”, “intermediate” or “high” probability groups. The diagnosis is then confirmed or rejected based on spirometry and/or a trial of treatment with corticosteroids (1).

Chronic obstructive pulmonary disorder (COPD), another respiratory obstructive disease that has a lot of symptoms in common with asthma can be identified with high PPV from the CPRD datasets using diagnostic Read codes alone (PPV=80%) or combined with COPD medications (PPV=90%) (4). The characteristic of COPD that best distinguishes it from asthma is the degree of reversibility of airflow obstruction, which is a central question in the questionnaire to be sent out to the GP’s (see appendix 2).

As the clinical examination necessary for the diagnosis of asthma is time and resource demanding, it would be useful for epidemiological studies to be able to obtain accurate records of asthma diagnosis within electronic databases of health-care records. The goal of this study is to understand and quantify how accurate asthma recording is in CPRD. When subsequent studies would be performed, it will be better understood how well the data reflects true diagnoses of asthma. A validation study of childhood asthma using General Practice Research Database (GPRD) data by using parental reports of a doctor’s diagnosis as the gold standard has been conducted and found a high sensitivity and specificity (5). A different study in Canada has validated asthma in patients older than 16 by comparing different information fields in electronic primary healthcare records without an external comparison (6). The CPRD database has been used in asthma studies because it captures a broad range of patients and goes back a long time. The current study will focus on the accuracy of asthma diagnosis recording in adults in CPRD, by measuring the PPV of different algorithms within the CPRD database and comparing it to a gold standard diagnosis given by the review of the answers of the GP questionnaire.

E. Study Type

This is a methodological study.

F. Study Design

This is a validation study of strategies or algorithms to ascertain asthma diagnosis recordings conducted in a retrospective cohort of asthma patients from the CPRD GOLD.

For peer review only - http://bmjopen.bmj.com/site/about/guidelines.xhtml

The random sample of individuals to be included in the study will be constructed from all participants registered in CPRD on or after 1 January 2004 who meet the inclusion criteria (see below). For the main analysis, a patient will be able to contribute to one algorithm only if an asthma medcode was recorded within the 24 month window prior to the end of data collection. It is possible an individual will be eligible for more than one algorithm depending on the Read codes used in their medical record. The individuals will be randomly selected from the algorithm with the fewest participants first and then removed from the cohort so that they cannot be selected for another algorithm. We have chosen this strategy (as opposed to an individual being eligible for a single algorithm only) because we want to test strategies to identify asthma patients from a single cohort rather than to test validity of the diagnosis. Further studies could then use a single strategy or their combination to extract an asthma cohort. There will be no special measures to ensure less frequent Read codes are used, because we assume the validity of asthma diagnosis strategy would be not be different between common and less frequent Read codes and the quality of recording would also be comparable. In addition, less frequent Read codes are unlikely to be used in isolation; our experience with validation of COPD recordings had shown that these infrequent Read codes are usually accompanied by other types of recordings.

Sample Size

The number of records for whom an asthma monitoring plan was started (medcode 81) exceeds 500,000 and the total number of asthma-related consultations exceeds 9,000,000 in the CPRD database.

Assuming an estimated PPV of 0.85 for each algorithm and an accuracy of the PPV (95% CI ± 0.08), a sample size of 77 individuals for each algorithm is needed.
A similar study conducted for COPD had a 77.6% response rate and 73.2% of the sent questionnaires were fit to be included in the final analysis (4). Considering a random sample of fully completed responses of 77 asthma patients for 8 algorithms is needed with 15% extra to account for a potential lower response rate, 750 questionnaires in total will be sent.

G. Data Linkage Required (if applicable)

The data linkage of CPRD-GOLD to MDI (Multiple Deprivation Index) is required to gather more information on the socio-economic status of the studied records. Ideally the MDI would be on patient level, if this is not available then the MDI on practice level would be used. We wish to request access to the IMD data linked to both the postcode of the GP practice and the patient's residential address (2010 version). We will take patient eligibility for linkage to IMD data into account when selecting our study population. We will also take the differences in methodology in IMD between the different countries of the UK into account.

H. Study Population

Inclusion Criteria

- Over 18 years old. People who become 18 after the study start can be included if they meet the criteria of an algorithm.
- Acceptable user status registered in CPRD.
- Practice is "up to standard" at study start 1/1/2004. From this date onwards, the Quality and Outcomes Framework (QOF) came in effect.

For peer review only - http://bmjopen.bmj.com/site/about/guidelines.xhtml

- The patient fits in one of the asthma algorithms within the last 24 months (see below)
- Patients are still alive and practice is currently still active in the CPRD.

Exclusion Criteria:

The patient does not fit the criteria of an algorithm group
Younger than 18 years

I. Selection of comparison group(s) or controls

There is no comparison group, as this is a validation study. The cohort will consist of only patients with a recording of asthma.

J. Exposures, Outcomes and Covariates

Exposure: Each patient included can contribute to only one algorithm or strategy (see appendix 3). If a patient is selected for a single algorithm (starting with the algorithm with the fewest participants), the patient will be excluded from the pool for the next algorithms. A preliminary code list for asthma diagnosis can also be found in appendix 1.
Covariates for stratification analysis:

-Age in years. All patients are 18 years or older, the categories will be based on the sample distribution.
-Gender as male or female
-Body Mass Index (BMI)
-Smoking status
-Other co-morbid conditions: COPD, atopy, GERD (Gastro-oesophageal Reflux Disease), eczema,rhinitis (including allergic rhinitis (hayfever) and chronic rhinosinusitis) and family history of asthma or atopy.
-Multiple deprivation Index

Outcome: recording of asthma diagnosis according to a specified algorithm and verified by the reference standard.

A number of different algorithms were constructed with degrees of certainty of asthma using separate indicators (see appendix 3). For example, the most stringent algorithm would include an asthma code, asthma medication and demonstrated reversibility after trial of treatment. Other algorithms would then drop one or more of these criteria. See appendix 3 for details of the algorithms.

A questionnaire will be sent to the general practitioners of a random sample of patients who fit in a certain algorithm to obtain information for the gold standard. A draft of the questionnaire can be found in appendix 2. The questionnaire is based on the "British guideline on the management of asthma" by the British Thoracic Society and Scottish Intercollegiate Guidelines Network (1).

K. Data/ Statistical analysis

The main analysis will be the calculation of the positive predictive value (the proportion of true positives) in each of the predefined algorithms. The gold standard consists of the opinion of 2 medical experts (Jennifer Quint and Daniel Morales)

For peer review only - http://bmjopen.bmj.com/site/about/guidelines.xhtml

independently reviewing the questionnaires and any additional supporting medical information provided. If there is a disagreement of diagnosis, the case would be discussed by the two experts. If an agreement cannot be found, a third opinion will be sought. Included in the main text.
Stratification analysis will be used to assess potential effect modification or confounding by covariates (see covariate list).

L. Plan for addressing confounding

Not applicable.

M. Plan for addressing missing data

We plan to do a complete case analysis, assuming that the probability of data being missing is independent of accuracy of the asthma diagnosis, conditional on covariates. If the amount of missing data is small, any violation of the assumption is unlikely to importantly affect the results. We anticipate a small degree of missingness for the BMI and smoking covariates.

N. Limitations of the study design, data sources and analytical methods

-Using a GP questionnaire as the source of patient information in order to obtain a gold standard to validate the asthma diagnosis can be problematic as the GP can consult the electronic health record to see if there was an asthma diagnosis. This will lead to an overestimation of the PPV. The GP's will be asked not to consult the CPRD records in the questionnaire.
-Incomplete diagnostic information will lead to missing data which we will be unaware of which could lead to some inaccuracy in PPV or classification of asthma probability.
-Only living patients will be assessed, as GP's no longer have access to the patient records after death. This excludes the records of the deceased patients and could result in survival bias.
-Miscoding accidents would lower the PPV.
-Response rate for the questionnaire might be lower than expected, and the sample size of the completed questionnaires could be too small.
-By focusing on the PPV, we will not be able to accurately assess the NPV, specificity or sensitivity. By preselecting the population of possible asthma cases, the NPV, specificity and sensitivity would be artificially manipulated. The NPV is the Negative Predictive Value: the proportion of negative results that are true negatives. -We are assuming that the validity of asthma diagnosis strategy would not be different between common and less frequent Read codes and the quality of recording would also be comparable for pragmatic reasons. In future practice when identifying patients with asthma, the less commonly used codes will continue to identify a smaller proportion of all asthma patients and so the validity we measure will apply to the majority of patients.
-We are also assuming that the probability of data being missing is independent of accuracy of the asthma diagnosis. We agree this assumption may not hold, but, we are even less likely to meet the assumptions needed for multiple imputation. However, we anticipate little missing relevant data in this study based on past research. In addition, the covariates are needed for stratification analysis only, rather than for adjustment. So we anticipate the impact of missing data to be low

For peer review only - http://bmjopen.bmj.com/site/about/guidelines.xhtml

-Not all GP practices contribute to CPRD, and patients might refuse to participate in the CPRD programme. This can result in selection bias.

O. Patient or user group involvement (if applicable)

Currently there is no plan to involve patients in the study. Depending on our findings it is possible we would seek patient engagement in further studies to help shape future research questionswith the help of general asthma patient groups.

P. Plans for disseminating and communicating study results, including the presence or absence of any restrictions on the extent and timing of publication

We will present our findings at national and international meetings and publish the results in a peer reviewed journal. We will not include any cells with counts less than five due to anonymity concerns.

Q. References

1. British Thoracic Society Scottish Intercollegiate Guidelines N. British Guideline on the Management of Asthma. Thorax. 2008;63 Suppl 4:iv1-121.
2. Global, regional, and national age–sex specific all-cause and cause-specific mortality for 240 causes of death, 1990–2013: a systematic analysis for the Global Burden of Disease Study 2013. The Lancet.385(9963):117-71.
3. NHS. http://www.nhs.uk/conditions/asthma: NHS; 2015 [cited 2015 06/11].
4. Quint JK, Mullerova H, DiSantostefano RL, Forbes H, Eaton S, Hurst JR, et al. Validation of chronic obstructive pulmonary disease recording in the Clinical Practice Research Datalink (CPRD-GOLD). BMJ Open. 2014;4(7):e005540.
5. Cornish RP, Henderson J, Boyd AW, Granell R, Van Staa T, Macleod J. Validating childhood asthma in an epidemiological study using linked electronic patient records. BMJ Open. 2014;4(4):e005345.
6. Xi N, Wallace R, Agarwal G, Chan D, Gershon A, Gupta S. Identifying patients with asthma in primary care electronic medical record systems: Chart analysis–based electronic algorithm validation study. Canadian Family Physician. 2015;61(10):e474-e83.

R. Amendment

March 2016

There were some slight changes to the questionnaire on advice from CPRD regarding the remuneration of the GP's. There were also some minor amendments to the questionnaire to clarify the procedure for returning the questionnaire and to insert the patient identifier tables we use. The sentence "To answer this questionnaire, please refrain from using the data recorded in CPRD as the aim of this study is to see how reliable CPRD is." was removed to avoid confusion.

March 2017

For peer review only - http://bmjopen.bmj.com/site/about/guidelines.xhtml

We would like to examine the additional information provided by the questionnaires sent to GP's to quantify the misdiagnosis of COPD in asthma patients in the UK. The symptoms of asthma and COPD overlap, and the differential diagnosis is not always trivial to make. Information on reversibility testing, the QOF indicators, smoking status, concurrent respiratory diseases and other sources including consultant and hospital discharge letters, lung function tests and radiography results was requested in the questionnaire (see attachment).

A review of this information by a respiratory consultant and study GP aims to identify the actual cases of COPD in confirmed asthma patients. This review is used as the gold standard to calculate the PPV, NPV, sensitivity and specificity of recorded GP diagnoses of COPD in the primary care records of asthma patients.

The specific objectives we would like to add to this study are to calculate the PPV, NPV, sensitivity and specificity of a COPD diagnosis recorded by a general practitioner in patients with a confirmed asthma diagnosis.

For peer review only - http://bmjopen.bmj.com/site/about/guidelines.xhtml

Appendices

Appendix 1: CPRD medcodes indicating asthma

medcode	readterm	Probable	Definite
78	asthma		1
81	asthma monitoring		1
185	acute exacerbation of asthma		1
232	asthma attack		1
233	severe asthma attack		1
719	h/o: asthma	1	
1208	childhood asthma	1	
1555	bronchial asthma		1
2290	allergic asthma		1
3018	mild asthma		1
3366	severe asthma		1
3458	occasional asthma		1
3665	late onset asthma		1
4442	asthma unspecified		1
4606	exercise induced asthma		1
4892	status asthmaticus nos		1
5138	patient in asthma study	1	
5267	intrinsic asthma		1
5515	seen in asthma clinic	1	
5627	hay fever with asthma		1
5798	chronic asthmatic bronchitis		1
5867	exercise induced asthma		1
6707	extrinsic asthma with asthma attack		1
7058	emergency admission, asthma		1
7146	extrinsic (atopic) asthma		1
7191	asthma limiting activities		1
7229	asthma prophylactic medication used	1	
7378	asthma management plan given		1
7416	asthma disturbing sleep		1
7731	pollen asthma		1
8335	asthma attack nos		1
8355	asthma monitored		1
9018	number of asthma exacerbations in past year		1
9552	change in asthma management plan		1
9663	step up change in asthma management plan		1
10043	asthma annual review		1
10274	asthma medication review		1

For peer review only

For peer review only - http://bmjopen.bmj.com/site/about/guidelines.xhtml

10487	asthma - currently active		1
11022	asthma trigger	1	
11370	asthma confirmed		1
11387	refuses asthma monitoring	1	
11673	excepted from asthma quality indicators: patient unsuitable	1	
11695	excepted from asthma quality indicators: informed dissent	1	
12987	late-onset asthma		1
13064	asthma severity		1
13065	moderate asthma		1
13066	asthma - currently dormant	1	
13173	asthma not disturbing sleep	1	
13174	asthma not limiting activities	1	
13175	asthma disturbs sleep frequently		1
13176	asthma follow-up		1
14777	extrinsic asthma without status asthmaticus		1
15248	hay fever with asthma		1
16070	asthma nos		1
16655	asthma monitoring admin.	1	
16667	asthma control step 2		1
16785	asthma control step 1		1
18141	asthma monitoring due	1	
18223	step down change in asthma management plan		1
18224	asthma control step 3		1
18323	intrinsic asthma with asthma attack		1
18692	exception reporting: asthma quality indicators	1	
18763	referral to asthma clinic	1	
19167	asthma monitoring by nurse		1
19519	asthma treatment compliance unsatisfactory		1
19520	asthma treatment compliance satisfactory		1
19539	asthma monitoring check done	1	
20422	asthma clinic administration	1	
20860	asthma control step 5		1
20886	asthma control step 4		1
21232	allergic asthma nec		1
22752	occupational asthma		1
24479	emergency asthma admission since last appointment		1
24506	further asthma - drug prevent.		1
24884	asthma causes daytime symptoms 1 to 2 times per week		1
25181	asthma restricts exercise		1
25705	asthma monitor 3rd letter	1	
25706	asthma monitor 2nd letter	1	
25707	asthma monitor 1st letter	1	
25791	asthma clinical management plan		1
25796	mixed asthma	1	

For peer review only

For peer review only - http://bmjopen.bmj.com/site/about/guidelines.xhtml

26496	health education - asthma	1	
26501	asthma never causes daytime symptoms		1
26503	asthma causes daytime symptoms most days		1
26504	asthma never restricts exercise		1
26506	asthma severely restricts exercise		1
26861	asthma sometimes restricts exercise		1
27926	extrinsic asthma with status asthmaticus		1
29325	intrinsic asthma without status asthmaticus		1
29645	asthma control step 0	1	
30308	dna - did not attend asthma clinic	1	
30382	asthma monitoring admin.nos	1	
30458	asthma monitoring by doctor		1
30815	asthma causing night waking		1
31135	asthma monitor phone invite	1	
31167	asthma night-time symptoms		1
31225	asthma causes daytime symptoms 1 to 2 times per month		1
35927	asthma leaflet given	1	
37943	asthma monitor verbal invite	1	
38143	asthma never disturbs sleep		1
38144	asthma limits walking up hills or stairs		1
38145	asthma limits walking on the flat		1
38146	asthma disturbs sleep weekly		1
39478	wood asthma		1
39570	asthma causes night symptoms 1 to 2 times per month		1
40823	brittle asthma		1
41017	aspirin induced asthma		1
41020	absent from work or school due to asthma		1
41554	asthma monitor offer default	1	
42824	asthma daytime symptoms		1
43770	asthma society member	1	
45073	intrinsic asthma nos		1
45782	extrinsic asthma nos		1
46529	attends asthma monitoring		1
47337	asthma accident and emergency attendance since last visit		1
47684	detergent asthma		1
58196	intrinsic asthma with status asthmaticus		1
73522	work aggravated asthma		1
92109	asthma outreach clinic	1	
93353	sequoiosis (red-cedar asthma)		1
93736	royal college of physicians asthma assessment		1
98185	asthma control test		1
99793	patient has a written asthma personal action plan		1
100107	health education - asthma self management		1
100397	asthma control questionnaire		1

For peer review only - http://bmjopen.bmj.com/site/about/guidelines.xhtml

100509	under care of asthma specialist nurse		1
100740	health education - structured asthma discussion		1
102170	asthma review using roy colleg of physicians three questions		1
102209	mini asthma quality of life questionnaire		1
102301	asthma trigger - seasonal		1
102341	asthma trigger - pollen		1
102395	asthma causes symptoms most nights		1
102400	asthma causes night time symptoms 1 to 2 times per week		1
102449	asthma trigger - respiratory infection		1
102713	asthma limits activities 1 to 2 times per month		1
102871	asthma trigger - exercise		1
102888	asthma limits activities 1 to 2 times per week		1
102952	asthma trigger - warm air		1
103318	health education - structured patient focused asthma discuss		1
103321	asthma trigger - animals		1
103612	asthma never causes night symptoms		1
103631	royal college physician asthma assessment 3 question score		1
103813	asthma trigger - cold air		1
103944	asthma trigger - airborne dust		1
103945	asthma trigger - damp		1
103952	asthma trigger - emotion		1
103955	asthma trigger - tobacco smoke		1
103998	asthma limits activities most days		1
105420	asthma self-management plan review		1
105674	asthma self-management plan agreed		1
106805	chronic asthma with fixed airflow obstruction		1
107167	number days absent from school due to asthma in past 6 month		1

For peer review only - http://bmjopen.bmj.com/site/about/guidelines.xhtml

Study into asthma: questionnaire for £55, further information for £55

The London School of Hygiene and Tropical Medicine is conducting a study to investigate the best way to identify asthma within the Clinical Practice Research Datalink (CPRD). We have developed several methods for identifying asthma in the database, and we would like to obtain some information on the current asthma status of the patient from GPs so that we can decide which method is the most suitable. We would be very grateful if you could supply us with the following information.

A. Do you agree this patient has a current diagnosis of asthma?

☐ Yes: Proceed to question B
☐ No: Proceed to question C
☐ Uncertain: Proceed to question B

If you answered yes or uncertain to question A:

B1. Has the diagnosis been made or confirmed by a respiratory physician?

☐ Yes
☐ No

B2. Does this patient have evidence of reversible airway obstruction?

☐ Yes
☐ No

If yes: **Was this based on;**

☐ Spirometry reversibility with a bronchodilator
☐ Trial of treatment with oral or inhaled corticosteroids and diurnal variation on a peak flow diary

B3. In what year was the asthma first diagnosed?

B4. Were any other factors taken into consideration in making the diagnosis?

	Yes	No
a. History of atopic disorder	☐	☐
b. Family history of asthma and/or atopic disorder	☐	☐
c. Widespread wheeze heard on auscultation of the chest	☐	☐
d. Otherwise unexplained low FEV (Forced Expiratory Volume) or PEF (Peak Expiratory Flow) on spirometry	☐	☐
e. Otherwise unexplained variability in PEFR (Peak Expiratory Flow Rate) on spirometry	☐	☐
f. Otherwise unexplained peripheral blood eosinophilia	☐	☐

For peer review only - http://bmjopen.bmj.com/site/about/guidelines.xhtml

g. FeNO (Fractional exhaled Nitric Oxide) measurement ☐ ☐
h. Other (please name)

B5. Based on the QOF (Quality and Outcomes Framework) indicators:

	Yes	No
a. Does the patient have any difficulty sleeping because of asthma symptoms, including cough	☐	☐
b. Does the patient have the usual asthma symptoms during the day (cough, wheeze, chest tightness of breathlessness)?	☐	☐
c. Does the asthma interfere with the patient's usual activities (housework, work, school, etc.)?	☐	☐

B6. What is the patient's smoking status?

☐ Current smoker
☐ Ex-smoker
☐ Never-smoker

B7. Does the patient have any other respiratory diseases? (Multiple responses possible)
☐ Chronic Obstructive Pulmonary Disease (COPD)
☐ Bronchiectasis
☐ Interstitial Lung Disease
☐ Other, please list:
☐ No

If you answered no to question A:
C. Do you think this patient has a history of asthma?
☐ Yes
☐ No
☐ Uncertain

Please provide anonymised copies of any additional relevant information allowing corroborating asthma diagnosis e.g. medical notes, discharge letters, test values. Payment for further information is £55 per patient.

Please return responses to CPRD in the freepost envelope provided or to our freepost address:
Freepost RSKH-TTAU-UKKX, CPRD, MHRA,
151 Buckingham Palace Rd, London, SW1W 9SZ

For peer review only

Appendix 3: Algorithms: all within the last 24 months

1. Definite asthma code + evidence of reversibility testing (spirometry or trial of treatment) *or variable PEFR* + more than one prescription of inhaled asthma therapy *(Inhaled SABA/LABA/CS)*
2. Definite asthma code + evidence of reversibility testing (spirometry or trial of treatment) *or variable PEFR*
3. Definite asthma code + more than one prescription of inhaled asthma therapy *(Inhaled SABA/LABA/CS)*
4. Definite asthma code only
5. Possible asthma code + evidence of reversibility testing (spirometry or trial of treatment) *or variable PEFR* + more than one prescription of inhaled asthma therapy *(Inhaled SABA/LABA/CS)*
6. Symptoms (wheeze, breathlessness, chest tightness, cough) + evidence of reversibility testing (spirometry or trial of treatment) *or variable PEFR* + more than one prescription of inhaled asthma therapy *(Inhaled SABA/LABA/CS)*
7. Symptoms (wheeze, breathlessness, chest tightness, cough) + evidence of reversibility testing (spirometry or trial of treatment) or variable PEFR
8. Symptoms (wheeze, breathlessness, chest tightness, cough) + more than one prescription of inhaled asthma therapy *(Inhaled SABA/LABA/CS)*

For peer review only

For peer review only - http://bmjopen.bmj.com/site/about/guidelines.xhtml

Section & Topic	No	Item	Reported on page #
**TITLE OR ABSTRACT**			
	1	Identification as a study of diagnostic accuracy using at least one measure of accuracy (such as sensitivity, specificity, predictive values, or AUC)	1
**ABSTRACT**			
	2	Structured summary of study design, methods, results, and conclusions (for specific guidance, see STARD for Abstracts)	2,3
**INTRODUCTION**			
	3	Scientific and clinical background, including the intended use and clinical role of the index test	5,6
	4	Study objectives and hypotheses	
**METHODS**			
Study design	5	Whether data collection was planned before the index test and reference standard were performed (prospective study) or after (retrospective study)	8
Participants	6	Eligibility criteria	8
	7	On what basis potentially eligible participants were identified (such as symptoms, results from previous tests, inclusion in registry)	8
	8	Where and when potentially eligible participants were identified (setting, location and dates)	8
	9	Whether participants formed a consecutive, random or convenience series	8
Test methods	10a	Index test, in sufficient detail to allow replication	12
	10b	Reference standard, in sufficient detail to allow replication	12
	11	Rationale for choosing the reference standard (if alternatives exist)	
	12a	Definition of and rationale for test positivity cut-offs or result categories of the index test, distinguishing pre-specified from exploratory	12
	12b	Definition of and rationale for test positivity cut-offs or result categories of the reference standard, distinguishing pre-specified from exploratory	12
	13a	Whether clinical information and reference standard results were available to the performers/readers of the index test	12
	13b	Whether clinical information and index test results were available to the assessors of the reference standard	12
Analysis	14	Methods for estimating or comparing measures of diagnostic accuracy	12-13
	15	How indeterminate index test or reference standard results were handled	12
	16	How missing data on the index test and reference standard were handled	9
	17	Any analyses of variability in diagnostic accuracy, distinguishing pre-specified from exploratory	12-13
	18	Intended sample size and how it was determined	13
**RESULTS**			
Participants	19	Flow of participants, using a diagram	Figure 1
	20	Baseline demographic and clinical characteristics of participants	Table 1
	21a	Distribution of severity of disease in those with the target condition	N/A
	21b	Distribution of alternative diagnoses in those without the target condition	N/A
	22	Time interval and any clinical interventions between index test and reference standard	13
Test results	23	Cross tabulation of the index test results (or their distribution) by the results of the reference standard	Table 2
	24	Estimates of diagnostic accuracy and their precision (such as 95% confidence intervals)	Table 2
	25	Any adverse events from performing the index test or the reference standard	N/A
**DISCUSSION**			
	26	Study limitations, including sources of potential bias, statistical uncertainty, and generalisability	18-20
	27	Implications for practice, including the intended use and clinical role of the index test	20
**OTHER INFORMATION**			
	28	Registration number and name of registry	20
	29	Where the full study protocol can be accessed	20
	30	Sources of funding and other support; role of funders	20-21

For peer review only

STARD 2015

AIM

STARD stands for "Standards for Reporting Diagnostic accuracy studies". This list of items was developed to contribute to the completeness and transparency of reporting of diagnostic accuracy studies. Authors can use the list to write informative study reports. Editors and peer-reviewers can use it to evaluate whether the information has been included in manuscripts submitted for publication.

EXPLANATION

A **diagnostic accuracy study** evaluates the ability of one or more medical tests to correctly classify study participants as having a **target condition.** This can be a disease, a disease stage, response or benefit from therapy, or an event or condition in the future. A medical test can be an imaging procedure, a laboratory test, elements from history and physical examination, a combination of these, or any other method for collecting information about the current health status of a patient.

The test whose accuracy is evaluated is called **index test.** A study can evaluate the accuracy of one or more index tests. Evaluating the ability of a medical test to correctly classify patients is typically done by comparing the distribution of the index test results with those of the **reference standard**. The reference standard is the best available method for establishing the presence or absence of the target condition. An accuracy study can rely on one or more reference standards.

If test results are categorized as either positive or negative, the cross tabulation of the index test results against those of the reference standard can be used to estimate the **sensitivity** of the index test (the proportion of participants *with* the target condition who have a positive index test), and its **specificity** (the proportion *without* the target condition who have a negative index test). From this cross tabulation (sometimes referred to as the contingency or "2x2" table), several other accuracy statistics can be estimated, such as the positive and negative **predictive values** of the test. Confidence intervals around estimates of accuracy can then be calculated to quantify the statistical **precision** of the measurements.

If the index test results can take more than two values, categorization of test results as positive or negative requires a **test positivity cut-off**. When multiple such cut-offs can be defined, authors can report a receiver operating characteristic (ROC) curve which graphically represents the combination of sensitivity and specificity for each possible test positivity cut-off. The **area under the ROC curve** informs in a single numerical value about the overall diagnostic accuracy of the index test.

The **intended use** of a medical test can be diagnosis, screening, staging, monitoring, surveillance, prediction or prognosis. The **clinical role** of a test explains its position relative to existing tests in the clinical pathway. A replacement test, for example, replaces an existing test. A triage test is used before an existing test; an add-on test is used after an existing test.

Besides diagnostic accuracy, several other outcomes and statistics may be relevant in the evaluation of medical tests. Medical tests can also be used to classify patients for purposes other than diagnosis, such as staging or prognosis. The STARD list was not explicitly developed for these other outcomes, statistics, and study types, although most STARD items would still apply.

DEVELOPMENT

This STARD list was released in 2015. The 30 items were identified by an international expert group of methodologists, researchers, and editors. The guiding principle in the development of STARD was to select items that, when reported, would help readers to judge the potential for bias in the study, to appraise the applicability of the study findings and the validity of conclusions and recommendations. The list represents an update of the first version, which was published in 2003.

More information can be found on http://www.equator-network.org/reporting-guidelines/stard.

For peer review only

CC BY SA

Validation of asthma recording in the Clinical Practice Research Datalink (CPRD)

Journal:	*BMJ Open*
Manuscript ID	bmjopen-2017-017474.R1
Article Type:	Research
Date Submitted by the Author:	21-Jun-2017

Complete List of Authors:	Nissen, Francis; London School of Hygiene and Tropical Medicine, EPH - ENCD<br>Morales, Daniel; University of Dundee, 2Division of Population Health Sciences<br>Müllerová, Hana; GlaxoSmithKline, RWE & Epidemiology, GSK R&D<br>Smeeth, Liam; London School of Hygiene and Tropical Medicine, Epidemiology and Population Health<br>Douglas, Ian; London School of Hygiene and Tropical Medicine, Epidemiology and Population Health<br>Quint, Jennifer; Imperial College London, Respiratory Epidemiology, Occupational Medicine and Public Health

**Primary Subject Heading**:	Epidemiology
Secondary Subject Heading:	Respiratory medicine, Health informatics, General practice / Family practice
Keywords:	Asthma < THORACIC MEDICINE, EPIDEMIOLOGY, Health informatics < BIOTECHNOLOGY & BIOINFORMATICS, Information management < BIOTECHNOLOGY & BIOINFORMATICS

SCHOLARONE™
Manuscripts

Validation of asthma recording in the Clinical Practice Research Datalink (CPRD)

Authors: Francis Nissen,[1] Daniel R.Morales,[2] Hana Mullerova,[3] Liam Smeeth,[1] Ian J Douglas,[1] Jennifer K Quint[4]

[1]Department of Non-Communicable Disease Epidemiology, London School of Hygiene and Tropical Medicine, London, UK; [2]Division of Population Health Sciences, University of Dundee, Dundee, UK; [3]RWE & Epidemiology, GSK R&D, Uxbridge, UK, [4]National Heart and Lung Institute, Imperial College, London, UK;

Correspondence:

Francis Nissen, MD, MSc, Department of Non-Communicable Disease Epidemiology, London School of Hygiene and Tropical Medicine, London, UK.

Address: Keppel Street, London, WC1E 7HT, UK

E-mail: francis.nissen@lshtm.ac.uk

+44 786 4314 923

For peer review only - http://bmjopen.bmj.com/site/about/guidelines.xhtml

For peer review only

ABSTRACT

Objectives: The optimal method of identifying people with asthma from electronic health records in primary care is not known. The aim of this study is to determine the Positive Predictive Value (PPV) of different algorithms using clinical codes and prescription data to identify people with asthma in the United Kingdom Clinical Practice Research Datalink (CPRD).

Methods: 684 participants registered with a GP practice contributing to CPRD between 1st of December 2013 and 30th of November 2015 were selected according to 1 of 8 pre-defined potential asthma identification algorithms. A questionnaire was sent to the general practitioners to confirm asthma status and provide additional information to support an asthma diagnosis. Two study physicians independently reviewed and adjudicated the questionnaires and additional information to form a gold standard for asthma diagnosis. The Positive Predictive Value was calculated for each algorithm.

Results: 684 questionnaires were sent, of which 494 (72%) were returned and 475 (69%) were complete and analysed. All 5 algorithms including a specific Read code indicating asthma or non-specific Read code accompanied by additional conditions performed well. The PPV for asthma diagnosis using only a specific asthma code was 86.4% (95% CI 77.4% to 95.4%). Extra information on asthma medication prescription (PPV 83.3%), evidence of reversibility testing (PPV 86.0%) or a combination of all three selection criteria (PPV 86.4%)

For peer review only

For peer review only - http://bmjopen.bmj.com/site/about/guidelines.xhtml

did not result in a higher PPV. The algorithm using non-specific asthma codes, information on reversibility testing, and respiratory medication use scored highest (PPV 90.7%, 95% CI (82.8% to 98.7%), but had a much lower identifiable population. Algorithms based on asthma symptom codes had low PPVs (43.1% to 57.8%).

Conclusions: People with asthma can be accurately identified from UK primary care records using specific Read codes. The inclusion of spirometry or asthma medications in the algorithm did not clearly improve accuracy.

Keywords

Asthma, Validation, Electronic Health Records, Positive Predictive Value, epidemiology

Word count: 3287

Article summary

Strengths:

This study describes algorithms to identify people with asthma from CPRD, a large electronic health records database, and measures the positive predictive value of those algorithms.

For peer review only - http://bmjopen.bmj.com/site/about/guidelines.xhtml

Supporting information, including outpatient referral letters, other emergency department discharge letters, airflow measurements and radiography records were used to identify asthma patients and calculate the test measures.

Limitations:

The gold standard to calculate a PPV (GP questionnaire and review by study physicians) is not absolute, even though information from secondary care was used.

GPs of patients with complicated medical histories could be less likely to return the questionnaire, but remuneration makes this less likely.

For peer review only

BACKGROUND

Asthma is one of the most common chronic diseases, with an estimated prevalence of 241 million people worldwide with asthma (1). The United Kingdom has one of the highest asthma prevalence and mortality rates in Europe (2, 3). The disease is a significant burden to the National Health Service, with 5.4 million people receiving treatment and approximately 65,000 hospital admissions yearly (4). Cough, wheeze, breathlessness and chest tightness are its core symptoms (5) but it has a wide variety of different presentations (6).

For peer review only - http://bmjopen.bmj.com/site/about/guidelines.xhtml

Electronic health records (EHR) have been adopted worldwide, facilitating the construction of large population-based patient databases that have become available over the last decades for epidemiological research (7). Validation of diagnoses or outcomes based upon codes recorded in EHRs is required because their accuracy is uncertain, and this may affect the reliability and validity of subsequent observational studies. The quality of studies generated from EHRs may be debatable unless their data are validated for specific research purposes (8-11).

The diagnosis of asthma relies on clinical judgement based on a combination of patient history, physical examination and confirmation of the variability or reversibility of airflow obstruction using airflow measurements. This can make it difficult to assess the accuracy of asthma diagnoses in EHR-based epidemiological studies as some symptoms and airflow measurements may not be recorded. In addition, individuals affected by asthma can vary greatly in their presentation and symptoms are sometimes similar to other respiratory diseases such as COPD (Chronic Obstructive Pulmonary Disease) (12, 13).

The aim of this study was to test the accuracy of different approaches to identifying asthma in the United Kingdom Clinical Practice Research Datalink (CPRD) using the positive predictive value (PPV), by comparing the database records with a gold standard constructed from a review by 2 study physicians based on information provided by asthma patients' GPs.

For peer review only - http://bmjopen.bmj.com/site/about/guidelines.xhtml

METHODS

Dataset

The Clinical Practice Research Datalink (CPRD) is a large UK primary care database containing anonymised data on the people registered with primary care practices from across the UK. CPRD is representative of the UK population with regard to age and sex (14, 15). Within CPRD, diagnostic accuracy has been demonstrated to be high for many conditions and diseases, including COPD (16-19). CPRD contains detailed clinical information on diagnoses, prescriptions, laboratory tests, symptoms and hospital referrals, in addition to basic sociodemographic information recorded by the general practitioners. These general practitioners (GPs) act as primary care providers and gatekeepers for other National Health Service services, and information from other healthcare providers is also transmitted back to the GP. Clinical events and diagnoses are coded as Read codes, a dictionary of clinical terms widely used in the UK National Health Services by both primary and secondary healthcare providers. Validation studies aid to ensure credibility and quality of epidemiological studies done in CPRD (10).

For peer review only - http://bmjopen.bmj.com/site/about/guidelines.xhtml

Inclusion criteria

The study population consisted of people who had a record for a Read code indicating possible asthma in the two years before the index date (1st of December 2015) and who were registered in a GP practice meeting CPRD quality criteria. The Read code list is included in appendix 1. The data collection was planned before the index test and reference standard were performed. This timespan was chosen for several reasons: to overcome potential changes in quality of asthma diagnosis and recording over time; to reduce the chance that the database records were out of date; and to ensure the medical records were still available to GPs. People were identified at random based on one of eight pre-defined algorithms exclusively, which means that we populated the algorithm resulting in the smallest population first and subsequently removed these people from the cohort, to prevent them from also being selected for another algorithm. We randomly selected 800 possible asthma cases for validation. Of these, 116 asthma cases were excluded because their GP no longer participated with CPRD at the time questionnaires were sent to the clinicians for validation, as shown in figure 1. Due to changes in CPRD data governance after the start of the study it was not possible to select replacement patients.

For peer review only - http://bmjopen.bmj.com/site/about/guidelines.xhtml

GP questionnaire

CPRD mailed a two page questionnaire to the GPs of the people selected for inclusion as described above, requesting confirmation of current asthma diagnosis and additional information to support this diagnosis. This questionnaire can be found in appendix 2. The questionnaire was designed to ascertain the diagnosis of asthma and verify the date of diagnosis. The questions included evidence of reversible airway obstruction, current symptoms, smoking history, respiratory comorbidities and Quality Outcome Framework (QOF) indicators. QOF is a national financial incentive scheme for GPs in the UK encouraging regular disease indicator measurement and recording. Asthma is one of the included diseases, and its indicators including airflow measurements and interference with work and night's rest (20).

Specific information available from the medical record including spirometry printouts and hospital respiratory outpatient letters were also requested. Data were encrypted twice to ensure anonymity, between practices and CPRD and also from CPRD to researchers. A questionnaire was considered invalid if it was returned blank or every question was answered "unknown".

Code lists and algorithms

Lists of medical codes (Read codes) deemed as specific and non-specific for asthma based on study physicians' opinion were created prior to the start of the study. Read codes are a hierarchical clinical coding system that are used in general practice in the UK and are entered by the GP into a computer programme called Vision. Each Read code is linked to a specific string of text, which refers to a single diagnosis or symptom. These data are then uploaded by CPRD after they have been processed and quality checked. The list of codes used for specific or definite asthma codes and nonspecific or probable asthma codes can be found in appendix 1.

Combinations of Read code lists, evidence of reversibility testing and respiratory medication use were used to make up the eight algorithms. The first four algorithms required a specific asthma diagnosis code, with the first three requiring additional documentation consisting of either respiratory medication use and/or evidence of reversibility testing. The fifth algorithm required a non-specific asthma code and additional documentation of both respiratory medications and reversibility testing; the last three algorithms required respiratory symptom codes indicating asthma symptoms with additional information. The presence of spirometry for inclusion in an algorithm was based on the existence of a specific spirometry Read code in the records rather than an examination of said spirometry, although where spirometry

For peer review only - http://bmjopen.bmj.com/site/about/guidelines.xhtml

traces were provided as part of the additional information, they were examined. Evidence of reversibility testing only refers to whether airflow measurements or trial of treatment were done, and does not reflect the results of these tests. Respiratory medication use was defined as at least two prescriptions of asthma medication for inhaled asthma therapy (Short Acting Beta-Agonists, Long Acting Beta-Agonists and Inhaled Corticosteroids) within 365 days of each other, within the two years before the index date. From the expected most specific to most sensitive, the eight algorithms were constructed as follows:

- 1. Specific asthma Read code + evidence of reversibility testing (spirometry, variable Peak Expiratory Flow Rate or trial of treatment) + respiratory medications
- 2. Specific asthma code + evidence of reversibility testing
- 3. Specific asthma code + respiratory medications
- 4. Specific asthma code only
- 5. Non-specific asthma code + evidence of reversibility testing + respiratory medications
- 6. Asthma Symptoms (wheeze, breathlessness, chest tightness, cough) + evidence of reversibility testing + respiratory medications
- 7. Asthma Symptoms + evidence of reversibility testing
- 8. Asthma Symptoms + respiratory medications

For peer review only - http://bmjopen.bmj.com/site/about/guidelines.xhtml

Primary outcome

The primary outcome was confirmation of a diagnosis of asthma in each of the eight predefined algorithms. The gold standard for the diagnosis of asthma was the adjudicated asthma status agreed by the two study physicians, a respiratory physician and a GP who reviewed all questionnaires and evidence from the patient's GP independently. The reviewers were blinded to the code lists/algorithm. Where opinion differed, the cases were discussed and agreement was reached by consensus. The reviewing physicians did not know with which algorithm a person was selected.

Statistical analysis

The Positive Predictive Value (PPV) was calculated using the proportion of cases identified by each algorithm that were confirmed as actual cases by the study physicians through a review of the questionnaire and supporting evidence. All analyses were conducted using Stata 14.0.

A patient could contribute only to a single algorithm for the main analysis. In the post hoc analysis, individuals could be placed into multiple algorithms where possible to reduce the confidence intervals. The PPV in this analysis was calculated for all individuals who had a specific asthma code compared to those with a specific asthma code and additional information. We also performed a sensitivity analysis to check whether the age and sex for

For peer review only - http://bmjopen.bmj.com/site/about/guidelines.xhtml

patients whose questionnaire was returned was similar to the age and sex of those patients whose questionnaire was not sent out or were there was no response. The study protocol is included in appendix 3.

Sample size calculation

As there were 116 patients that could not be evaluated, precision was expected to be slightly lower than in the original sample size calculations. However, a percentage difference in PPV of 0.13 is demonstrable with a sample size of 60 per algorithm (assuming PPV=0.85, alpha=0.05 and power=0.8).

RESULTS

A total of 800 potential asthma cases were selected for validation, of which 116 cases had migrated out of the database at the time the questionnaires were sent. Of the remaining 684 cases, there were 494 returned questionnaires. Nineteen of the returned questionnaires were considered invalid. Thus, 475 valid questionnaires were received, which yielded a response rate of 69.4% (475/684) using the practices that could have answered as denominator, as shown in figure 1. The time interval between the mailing of questionnaires and the review by the study physicians varied, but none of these time intervals was greater than 8 months.

review only

For peer review only - http://bmjopen.bmj.com/site/about/guidelines.xhtml

Algorithm	1. specific asthma code + reversibility testing + medication	2. specific asthma code + reversibility testing	3. specific asthma code + medication	4. specific asthma code	5. non-specific asthma code + reversibility testing + medication	6. symptoms + reversibility testing + medication	7. symptoms + reversibility testing	8. symptoms + medication	Total
**Individuals, N (%)**	68 (100)	57 (100)	60 (100)	59 (100)	54 (100)	55 (100)	58 (100)	64 (100)	475
**Asthma diagnosis by patient's GP**	56 (82.4)	49 (86)	48 (80)	51 (86.4)	48 (88.9)	29 (52.7)	23 (39.7)	38 (59.4)	342
**Confirmation by respiratory physician before study start**	55 (80.9)	29 (50.9)	38 (63.3)	45 (76.3)	34 (63)	23 (41.8)	25 (43.1)	36 (56.3)	285
**Evidence of reversible airway obstruction**	47 (69.1)	37 (64.9)	32 (53.3)	32 (54.2)	31 (57.4)	26 (47.3)	19 (32.8)	26 (40.6)	250
**Mean age**	52.3	51.4	47	41.9	45	60.9	61.3	52.1	
**Mean age: (95% CI)**	(47.4 to 57.2)	(46.2 to 56.7)	(41.4 to 52.6)	(36.1 to 47.6)	(38.7 to 51.3)	(55.3 to 66.4)	(57.1 to 65.5)	(45.4 to 58.7)	
**< 18 years old (%)**	7.35	7.02	15.25	18.64	16.67	7.27	1.72	20.31	11.81
**Sex: male**	31 (45.6)	17 (29.8)	16 (26.7)	23 (39)	26 (48.1)	28 (50.9)	24 (41.4)	31 (48.4)	196
**Current smoker***	11 (16.2)	10 (17.5)	10 (16.7)	5 (8.5)	4 (7.4)	5 (9.1)	8 (13.8)	4 (6.3)	57
**Ex-smoker***	16 (23.5)	14 (24.6)	17 (28.3)	16 (27.1)	15 (27.8)	11 (20)	10 (17.2)	12 (18.8)	111
**Never smoker***	35 (51.5)	26 (45.6)	25 (41.7)	36 (61.0)	32 (59.3)	18 (32.7)	11 (19.0)	27 (42.2)	210
**Individuals with supporting info**	23 (33.8)	21 (36.8)	22 (36.7)	14 (23.7)	14 (25.9)	17 (30.9)	14 (24.1)	22 (34.4)	147

** as stated by patient's GP on the study questionnaire*

Table 1: Characteristics of the 475 patients included in the final study analysis

Algorithm	Eligible population	Questionnaires sent out	Valid returned questionnaires (N,%)	Confirmed asthma cases	PPV (95% CI)
1. specific asthma code + reversibility testing + medication	36,516	92	68 (60)	61	86.8 (78.5 to 95.0)
2. specific asthma code + reversibility testing	38,796	90	57 (63.3)	51	86.0 (76.7 to 95.3)
3. specific asthma code + medication	169,574	89	60 (67.4)	51	83.3 (73.6 to 93.0)
4. specific asthma code	188,133	84	59 (70.2)	51	86.4 (77.4 to 95.4)
5. non-specific asthma code + reversibility testing + medication	33,280	78	54 (69.2)	49	90.7 (82.8 to 98.7)
6. symptoms + reversibility testing + medication	53,117	87	55 (63.2)	32	56.4 (42.8 to 69.9)
7. symptoms + reversibility testing	66,477	88	58 (65.9)	26	43.1 (30.0 to 56.2)
8. symptoms + medication	190,753	78	64 (82.1)	38	57.8 (45.4 to 70.2)

Medication use was defined as two prescriptions within 365 days. Evidence of reversibility testing does not hold information on the outcome of these tests.

Table 2: The Positive Predictive Value (PPV) and proportion of patients diagnosed with Chronic Obstructive Pulmonary Disease (COPD) within each algorithm

For peer review only - http://bmjopen.bmj.com/site/about/guidelines.xhtml

The baseline characteristics of the 475 patients with valid returned questionnaires are shown in table 1. The study populations were mostly middle aged, never smokers and female. There were 97 individuals whose smoking status was not filled in on the questionnaire. Differences in the majority of characteristics were seen among most algorithms.

The positive predictive values of the eight algorithms are displayed in table 2.

The PPVs of algorithms containing specific or non-specific asthma codes in algorithms 1-5 (ranging from 83.3% to 90.7%) are markedly higher than the PPVs of the algorithms based on asthma symptoms (ranging from 43.1% to 57.8%). The combination of a specific code and asthma medication prescription and/or evidence of reversibility testing (PPV varies from 83.3% to 86.8%) did not considerably increase the PPV compared to a specific asthma code alone (PPV 86.4%). The highest PPV was found in the fifth algorithm combining a non-specific asthma code with evidence of reversibility testing and asthma medication use. However, the total number of patients identifiable with this algorithm (n=33,280) was less than one fifth of those identifiable by the fourth algorithm consisting of a specific asthma code alone (n=188,133) in the chosen time period. We have not examined the validity of a non-specific asthma code alone.

A post hoc analysis was performed where individuals were placed in every algorithm they qualified for. In this analysis, we found that the use of additional information on evidence of

For peer review only

For peer review only - http://bmjopen.bmj.com/site/about/guidelines.xhtml

reversibility testing or medication in an algorithm with a specific asthma code again did not meaningfully increase the PPV. The PPV for all individuals who had a specific asthma code and information on reversibility testing or medication was 86.7% (95% CI 83.3% to 90.1%), and the PPV for individuals with only a specific asthma code was 86.4% (95% CI 83.0% to 89.7%).

When validating the record of possible asthma with a gold standard based on the study physicians' view of extra evidence provided by the GP, the PPV slightly improved across all algorithms. Figure 2 demonstrates the PPV of the different algorithms as diagnosed by the patient's own GP and the study physicians (overall κ=0.81).

There was no considerable difference in age or sex between patients whose questionnaire was returned and patients whose questionnaire was not sent out (age: p=0.74, sex: p=0.73) or were there was no response (age p=0.50, sex p=0.13) using χ^2 tests.

For peer review only - http://bmjopen.bmj.com/site/about/guidelines.xhtml

DISCUSSION

We tested the accuracy of eight algorithms to identify asthma within CPRD using a gold standard constructed using a consensus of the two study physicians. The algorithm with the highest PPV consisted of a combination for nonspecific asthma codes, evidence of reversibility testing and multiple asthma prescriptions within one year (PPV 90.7, 95% CI 82.8 to 0.98.7) followed by a combination for specific asthma codes, evidence of reversibility testing and multiple asthma prescriptions within one year. The confidence interval of this PPV overlaps with the confidence intervals of each of the PPVs of the first four algorithms based on specific asthma codes, so the difference might be due to chance alone. The algorithm with the lowest PPV consisted of asthma symptoms and evidence of reversibility testing (PPV 0.43, 95% CI 0.30-0.55). The results of this validation study suggest that the clinical code based algorithms that use asthma codes to identify asthma cases have high PPVs (between 0.84 and 0.91). In this dataset, a specific asthma code algorithm alone appears sufficient to identify current asthma patients from CPRD. As the additional requirements of medication use and evidence of reversibility testing do not appear to significantly increase the PPV, the total number of individuals who can potentially be included in a study increases from 33,280 to 188,133 in the chosen time period (1st of December 2013 to 30th of November 2015). The total identifiable population of people living with asthma is thus much larger when only using a specific asthma code for identification.

For peer review only - http://bmjopen.bmj.com/site/about/guidelines.xhtml

Comparison with previous studies

Validity of asthma codes in electronic health records can be assessed by comparison to three different sets of gold standard: comparison to an external database, questionnaire and manual review by a clinician. This validation study uses questionnaires and manual review. Our gold standard consisted of the agreement of the study respiratory physician and study GP, both of whom were experienced with CPRD.

Previous studies which validated asthma in other EHR databases used manual review by clinicians to validate asthma in EHR and all reported at least one algorithm with a PPV above 85% (21-26). In contrast with this study, the best results in previous studies arose when combining diagnostic data and prescription data.

The CPRD has provided anonymised primary care records for public health research since 1987; research was always a focus of interest when it was established. GPs contributing to the CPRD have been trained on how to record data for research use. As a consequence, data quality may be higher than in many other databases, in which research is only a secondary product.

Strengths of this study

For peer review only - http://bmjopen.bmj.com/site/about/guidelines.xhtml

This study has several strengths. First, we were able to investigate the accuracy of eight pre-defined different algorithms and how they perform in identification of people with asthma in CPRD, as well as the accuracy of the actual GP diagnosis of asthma using additional information provided. Second, we included supporting information such as outpatient referral letters, other emergency department discharge letters, airflow measurements and radiography records. Finally, we validated asthma diagnoses found in CPRD, which is a primary care database that is extensively used for studying different health outcomes in epidemiological research. This primary care database provides health and medication history of millions of patients. A validated definition in CPRD of asthma allows for informed health-care service planning by increasing the reliability of evidence generated from observational studies.

Limitations of this study

This study has limitations to consider. The gold standard consisting of a GP questionnaire and review by study physicians is not absolute, even if we mitigated this with additional information from secondary care. A GP can look in the electronic health record to see if a specific diagnosis has been recorded for a specific patient when asked. This may lead to an overestimation of the PPV, but there is no suitable practical alternative. Ideally, airflow measurements and reversibility testing on each potential patient would form the optimal gold standard, but this would not be feasible in this setting due to cost. The overall number of questionnaires sent out (n=684) was less than requested (n=800) as some patients and

For peer review only - http://bmjopen.bmj.com/site/about/guidelines.xhtml

practices were no longer part of CPRD and could not be contacted. However, the precision of PPV estimates was not substantially reduced.

Although practices contributing to CPRD are a sample of all practices in the UK, they are considered representative of the UK population with few patients opting out of contributing data, and is therefore unlikely to bias the results (14).

GPs of patients with complicated medical histories could be less likely to return the questionnaire. The GPs were remunerated for their participation however, which is likely to have reduced the chance of this happening. Within the returned questionnaires, the amount of missing data was low, which suggests reasonable data quality. In addition, only living patients were assessed, as GPs no longer have access to the patient records after death. This excludes the records of the deceased patients and could result in survival bias. Patients had to be alive to be included, but it is unlikely that coding would differ between living and deceased individuals. If deceased people had died of asthma, the PPV in this study would be underestimated. Our findings are likely to be generalizable to other UK primary care databases using Read coding, but these would ideally still require validation. Databases using other coding systems may need to validate different algorithms to identify asthma, which might limit the generalisability of our findings. Another limitation is that we were not able to assess the Negative Predictive Value (NPV) of asthma diagnoses in CPRD because we evaluated only patients belonging to one of the eight algorithms. We could not calculate the

For peer review only - http://bmjopen.bmj.com/site/about/guidelines.xhtml

specificity or sensitivity as we had preselected our population of possible asthma cases. We also assumed the validity of asthma diagnoses would not be different between common and less frequent Read codes and the quality of recording would also be comparable for pragmatic reasons. However, the less commonly used codes will by definition identify a smaller proportion of all asthma patients, so the validity we report will apply to the majority of patients.

CONCLUSION

We have successfully estimated the PPV of several different algorithms to identify people with asthma in CPRD. The PPVs for specific asthma Read codes alone and non-specific ones in a combination with additional evidence were all greater than 0.84. A specific asthma code algorithm alone appears to be the most practical approach to identify patients with asthma in CPRD (PPV=0.86; 95% CI 0.77-0.95). Diagnoses were confirmed in a high proportion of patients with specific asthma codes, suggesting that epidemiological asthma research conducted using CPRD data can be conducted with reasonably high validity.

Dissemination and ethics

The protocol for this research was approved by the Independent Scientific Advisory Committee (ISAC) for MHRA Database Research (protocol number15_257) and the

For peer review only - http://bmjopen.bmj.com/site/about/guidelines.xhtml

approved protocol was made available to the journal and reviewers during peer review. Generic ethical approval for observational research using the CPRD with approval from ISAC has been granted by a Health Research Authority (HRA) Research Ethics Committee (East Midlands – Derby, REC reference number 05/MRE04/87).

The results will be submitted for publication and will be disseminated through research conferences and peer reviewed journals.

Funding statement

This work was supported by GlaxoSmithKline (GSK), through a PhD scholarship for FN with grant number EPNCZF5310. The publishing of this study was supported by the Wellcome Trust: grant number 098504/Z/12/Z.

Competing interests

FN is funded by a GSK scholarship during his PhD program. IJD is funded by an unrestricted grant from, has consulted for, and holds stock in GlaxoSmithKline. HM is an employee of GSK R&D and own shares of GSK Plc. JKQ reports grants from MRC, BLF, Wellcome

For peer review only - http://bmjopen.bmj.com/site/about/guidelines.xhtml

Trust and has received research funds from GSK, AZ, Quintiles IMS, in addition to personal fees from AZ, Chiesi, BI .

Contributors

JKQ, IJD, LS and HM were responsible for developing the research question and have advised on the data collection and search strategies. FN summarised and analysed the questionnaires and drafted the manuscript. JKQ and DM reviewed the questionnaires and constructed the gold standard for asthma validation. JKQ is responsible for study management and coordination. All authors have read, commented on and approved the final manuscript.

Data sharing statement

Study data will be available on request to FN once the research team has completed pre-planned analyses.

Figure legend

Figure 1: Study population

Figure 2: PPV as diagnosed by the patient's own GP, and agreement between the study physicians

Table legend

Table 3: Characteristics of the 475 patients included in the final study analysis

Table 4: The Positive Predictive Value (PPV) and proportion of patients diagnosed with Chronic Obstructive Pulmonary Disease (COPD) within each algorithm

For peer review only - http://bmjopen.bmj.com/site/about/guidelines.xhtml

Appendices

1. Appendix 1: CPRD Read codes indicating asthma

2. Appendix 2: General Practitioner questionnaire

3. Appendix 3: ISAC study protocol

For peer review only

For peer review only - http://bmjopen.bmj.com/site/about/guidelines.xhtml

References

1. Global, regional, and national age-sex specific all-cause and cause-specific mortality for 240 causes of death, 1990-2013: a systematic analysis for the Global Burden of Disease Study 2013. Lancet. 2015;385(9963):117-71.

2. To T, Stanojevic S, Moores G, Gershon AS, Bateman ED, Cruz AA, et al. Global asthma prevalence in adults: findings from the cross-sectional world health survey. BMC Public Health. 2012;12:204.

3. The Global Asthma Report 2014. 2014.

4. Why asthma still kills. Royal College of Physicians, 2014.

5. James DR, Lyttle MD. British guideline on the management of asthma: SIGN Clinical Guideline 141, 2014. Arch Dis Child Educ Pract Ed. 2016;101(6):319-22.

6. Haldar P, Pavord ID, Shaw DE, Berry MA, Thomas M, Brightling CE, et al. Cluster analysis and clinical asthma phenotypes. Am J Respir Crit Care Med. 2008;178(3):218-24.

7. Langan SM, Benchimol EI, Guttmann A, Moher D, Petersen I, Smeeth L, et al. Setting the RECORD straight: developing a guideline for the REporting of studies Conducted using Observational Routinely collected Data. Clin Epidemiol. 2013;5:29-31.

8. Denney MJ, Long DM, Armistead MG, Anderson JL, Conway BN. Validating the extract, transform, load process used to populate a large clinical research database. Int J Med Inform. 2016;94:271-4.

9. Lo Re V, 3rd, Haynes K, Forde KA, Localio AR, Schinnar R, Lewis JD. Validity of The Health Improvement Network (THIN) for epidemiologic studies of hepatitis C virus infection. Pharmacoepidemiol Drug Saf. 2009;18(9):807-14.

10. Ehrenstein V, Petersen I, Smeeth L, Jick SS, Benchimol EI, Ludvigsson JF, et al. Helping everyone do better: a call for validation studies of routinely recorded health data. Clin Epidemiol. 2016;8:49-51.

11. ENCePP. ENCePP Guide on Methodological Standards in Pharmacoepidemiology: ENCePP; 2017 [cited 2017 31/03/2017]. Available from: http://www.encepp.eu/standards_and_guidances/methodologicalGuide3_2.shtml.

12. Bousquet J, Mantzouranis E, Cruz AA, Ait-Khaled N, Baena-Cagnani CE, Bleecker ER, et al. Uniform definition of asthma severity, control, and exacerbations: document presented for the World Health Organization Consultation on Severe Asthma. J Allergy Clin Immunol. 2010;126(5):926-38.

13. Sin DD, Miravitlles M, Mannino DM, Soriano JB, Price D, Celli BR, et al. What is asthma-COPD overlap syndrome? Towards a consensus definition from a round table discussion.

For peer review only - http://bmjopen.bmj.com/site/about/guidelines.xhtml

Eur Respir J. 2016;48(3):664-73.

14. Herrett E, Gallagher AM, Bhaskaran K, Forbes H, Mathur R, van Staa T, et al. Data Resource Profile: Clinical Practice Research Datalink (CPRD). Int J Epidemiol. 2015;44(3):827-36.

15. Williams T, van Staa T, Puri S, Eaton S. Recent advances in the utility and use of the General Practice Research Database as an example of a UK Primary Care Data resource. Ther Adv Drug Saf. 2012;3(2):89-99.

16. Herrett E, Thomas SL, Schoonen WM, Smeeth L, Hall AJ. Validation and validity of diagnoses in the General Practice Research Database: a systematic review. Br J Clin Pharmacol. 2010;69(1):4-14.

17. Thomas KH, Davies N, Metcalfe C, Windmeijer F, Martin RM, Gunnell D. Validation of suicide and self-harm records in the Clinical Practice Research Datalink. Br J Clin Pharmacol. 2013;76(1):145-57.

18. Rothnie KJ, Müllerová H, Hurst JR, Smeeth L, Davis K, Thomas SL, et al. Validation of the Recording of Acute Exacerbations of COPD in UK Primary Care Electronic Healthcare Records. PLoS One. 2016;11(3).

19. Quint JK, Müllerova H, DiSantostefano RL, Forbes H, Eaton S, Hurst JR, et al. Validation of chronic obstructive pulmonary disease recording in the Clinical Practice Research Datalink (CPRD-GOLD). BMJ Open. 2014;4(7).

20. Chew-Graham CA, Hunter C, Langer S, Stenhoff A, Drinkwater J, Guthrie EA, et al. How QOF is shaping primary care review consultations: a longitudinal qualitative study. BMC Fam Pract. 2013;14:103.

21. Xi N, Wallace R, Agarwal G, Chan D, Gershon A, Gupta S. Identifying patients with asthma in primary care electronic medical record systems Chart analysis-based electronic algorithm validation study. Canadian Family Physician. 2015;61(10):e474-83.

22. Kozyrskyj AL, HayGlass KT, Sandford AJ, Pare PD, Chan-Yeung M, Becker AB. A novel study design to investigate the early-life origins of asthma in children (SAGE study). Allergy. 2009;64(8):1185-93.

23. Pacheco JA, Avila PC, Thompson JA, Law M, Quraishi JA, Greiman AK, et al. A highly specific algorithm for identifying asthma cases and controls for genome-wide association studies. AMIA Annual Symposium Proceedings/AMIA Symposium. 2009;2009:497-501.

24. Vollmer WM, O'Connor EA, Heumann M, Frazier EA, Breen V, Villnave J, et al. Searching multiple clinical information systems for longer time periods found more prevalent cases of asthma. Journal of Clinical Epidemiology. 2004;57(4):392-7.

25. Donahue JG, Weiss ST, Goetsch MA, Livingston JM, Greineder DK, Platt R. Assessment of asthma using automated and full-text medical records. Journal of Asthma. 1997;34(4):273-81.

26. Premaratne UN, Marks GB, Austin EJ, Burney PGJ. A reliable method to retrieve Accident and Emergency data stored on a free-text basis. Respiratory Medicine. 1997;91(2):61-6.

For peer review only - http://bmjopen.bmj.com/site/about/guidelines.xhtml

For peer review only

For peer review only - http://bmjopen.bmj.com/site/about/guidelines.xhtml

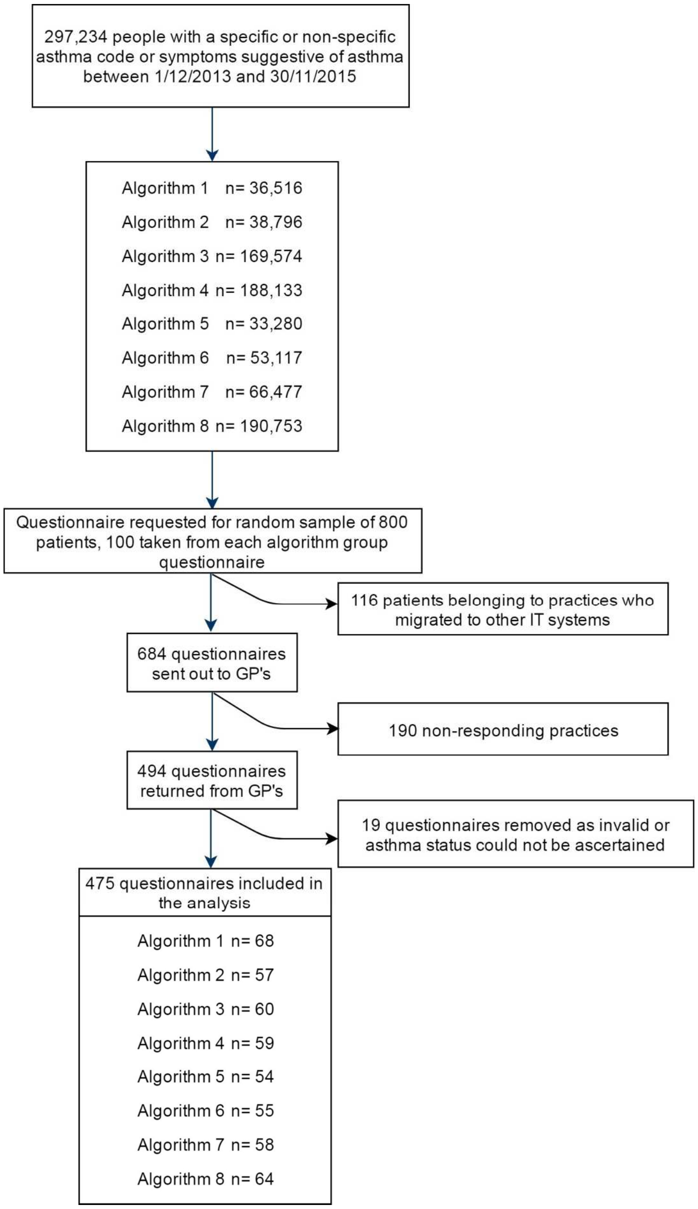

Figure 1: Study population

161x280mm (300 x 300 DPI)

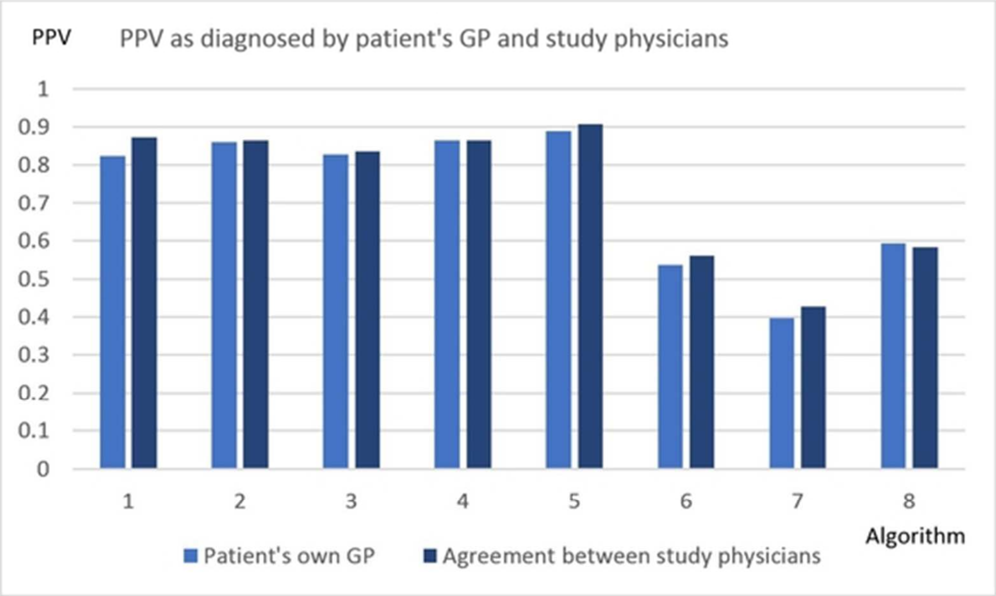

Figure 2: PPV as diagnosed by the patient's own GP, and agreement between the study physicians

49x29mm (300 x 300 DPI)

For peer review only

Appendix 1: CPRD medcodes indicating asthma

A) Specific asthma codes

medcode	readterm
78	asthma
81	asthma monitoring
185	acute exacerbation of asthma
232	asthma attack
233	severe asthma attack
1555	bronchial asthma
2290	allergic asthma
3018	mild asthma
3366	severe asthma
3458	occasional asthma
3665	late onset asthma
4442	asthma unspecified
4606	exercise induced asthma
4892	status asthmaticus nos
5267	intrinsic asthma
5627	hay fever with asthma
5798	chronic asthmatic bronchitis
5867	exercise induced asthma
6707	extrinsic asthma with asthma attack
7058	emergency admission, asthma
7146	extrinsic (atopic) asthma
7191	asthma limiting activities
7378	asthma management plan given
7416	asthma disturbing sleep
7731	pollen asthma
8335	asthma attack nos
8355	asthma monitored
9018	number of asthma exacerbations in past year
9552	change in asthma management plan
9663	step up change in asthma management plan
10043	asthma annual review
10274	asthma medication review
10487	asthma - currently active
11370	asthma confirmed
12987	late-onset asthma
13064	asthma severity
13065	moderate asthma
13175	asthma disturbs sleep frequently
13176	asthma follow-up

For peer review only

14777	extrinsic asthma without status asthmaticus
15248	hay fever with asthma
16070	asthma nos
16667	asthma control step 2
16785	asthma control step 1
18223	step down change in asthma management plan
18224	asthma control step 3
18323	intrinsic asthma with asthma attack
19167	asthma monitoring by nurse
19519	asthma treatment compliance unsatisfactory
19520	asthma treatment compliance satisfactory
20860	asthma control step 5
20886	asthma control step 4
21232	allergic asthma nec
22752	occupational asthma
24479	emergency asthma admission since last appointment
24506	further asthma - drug prevent.
24884	asthma causes daytime symptoms 1 to 2 times per week
25181	asthma restricts exercise
25791	asthma clinical management plan
26501	asthma never causes daytime symptoms
26503	asthma causes daytime symptoms most days
26504	asthma never restricts exercise
26506	asthma severely restricts exercise
26861	asthma sometimes restricts exercise
27926	extrinsic asthma with status asthmaticus
29325	intrinsic asthma without status asthmaticus
30458	asthma monitoring by doctor
30815	asthma causing night waking
31167	asthma night-time symptoms
31225	asthma causes daytime symptoms 1 to 2 times per month
38143	asthma never disturbs sleep
38144	asthma limits walking up hills or stairs
38145	asthma limits walking on the flat
38146	asthma disturbs sleep weekly
39478	wood asthma
39570	asthma causes night symptoms 1 to 2 times per month
40823	brittle asthma
41017	aspirin induced asthma
41020	absent from work or school due to asthma
42824	asthma daytime symptoms
45073	intrinsic asthma nos

For peer review only

45782	extrinsic asthma nos
46529	attends asthma monitoring
47337	asthma accident and emergency attendance since last visit
47684	detergent asthma
58196	intrinsic asthma with status asthmaticus
73522	work aggravated asthma
93353	sequoiosis (red-cedar asthma)
93736	royal college of physicians asthma assessment
98185	asthma control test
99793	patient has a written asthma personal action plan
100107	health education - asthma self management
100397	asthma control questionnaire
100509	under care of asthma specialist nurse
100740	health education - structured asthma discussion
102170	asthma review using roy colleg of physicians three questions
102209	mini asthma quality of life questionnaire
102301	asthma trigger - seasonal
102341	asthma trigger - pollen
102395	asthma causes symptoms most nights
102400	asthma causes night time symptoms 1 to 2 times per week
102449	asthma trigger - respiratory infection
102713	asthma limits activities 1 to 2 times per month
102871	asthma trigger - exercise
102888	asthma limits activities 1 to 2 times per week
102952	asthma trigger - warm air
103318	health education - structured patient focused asthma discuss
103321	asthma trigger - animals
103612	asthma never causes night symptoms
103631	royal college physician asthma assessment 3 question score
103813	asthma trigger - cold air
103944	asthma trigger - airborne dust
103945	asthma trigger - damp
103952	asthma trigger - emotion
103955	asthma trigger - tobacco smoke
103998	asthma limits activities most days
105420	asthma self-management plan review
105674	asthma self-management plan agreed
106805	chronic asthma with fixed airflow obstruction
107167	number days absent from school due to asthma in past 6 month

For peer review only

B) Non-specific asthma codes

medcode	readterm
719	h/o: asthma
1208	childhood asthma
5138	patient in asthma study
5515	seen in asthma clinic
7229	asthma prophylactic medication used
11022	asthma trigger
11387	refuses asthma monitoring
11673	excepted from asthma quality indicators: patient unsuitable
11695	excepted from asthma quality indicators: informed dissent
13066	asthma - currently dormant
13173	asthma not disturbing sleep
13174	asthma not limiting activities
16655	asthma monitoring admin.
18141	asthma monitoring due
18692	exception reporting: asthma quality indicators
18763	referral to asthma clinic
19539	asthma monitoring check done
20422	asthma clinic administration
25705	asthma monitor 3rd letter
25706	asthma monitor 2nd letter
25707	asthma monitor 1st letter
25796	mixed asthma
26496	health education - asthma
29645	asthma control step 0
30308	dna - did not attend asthma clinic
30382	asthma monitoring admin.nos
31135	asthma monitor phone invite
35927	asthma leaflet given
37943	asthma monitor verbal invite
41554	asthma monitor offer default
43770	asthma society member
92109	asthma outreach clinic

For peer review only

Study into asthma: questionnaire for £55, further information for £55

The London School of Hygiene and Tropical Medicine is conducting a study to investigate the best way to identify asthma within the Clinical Practice Research Datalink (CPRD). We have developed several methods for identifying asthma in the database, and we would like to obtain some information on the current asthma status of the patient from GPs so that we can decide which method is the most suitable.

We would be very grateful if you could supply us with the following information.

A. **Do you agree this patient has a current diagnosis of asthma?**

☐ Yes: Proceed to question B

☐ No: Proceed to question C

☐ Uncertain: Proceed to question B

If you answered yes or uncertain to question A:

B1. **Has the diagnosis been made or confirmed by a respiratory physician?**

☐ Yes

☐ No

B2. **Does this patient have evidence of reversible airway obstruction?**

☐ Yes

☐ No

If yes: **Was this based on;**

☐ Spirometry reversibility with a bronchodilator

☐ Trial of treatment with oral or inhaled corticosteroids and diurnal variation on a peak flow diary

B3. **In what year was the asthma first diagnosed?**

B4. **Were any other factors taken into consideration in making the diagnosis?**

		Yes	No
a.	History of atopic disorder	☐	☐
b.	Family history of asthma and/or atopic disorder	☐	☐
c.	Widespread wheeze heard on auscultation of the chest	☐	☐
d.	Otherwise unexplained low FEV (Forced Expiratory Volume) or PEF (Peak Expiratory Flow) on spirometry	☐	☐
e.	*Otherwise unexplained variability in PEFR (Peak Expiratory Flow Rate) on spirometry*	☐	☐

For peer review only

f.	Otherwise unexplained peripheral blood eosinophilia	☐	☐
g.	FeNO (Fractional exhaled Nitric Oxide) measurement	☐	☐
h.	Other (please name)		

B5. Based on the QOF (Quality and Outcomes Framework) indicators:

		Yes	No
a.	Does the patient have any difficulty sleeping because of asthma symptoms, including cough	☐	☐
b.	Does the patient have the usual asthma symptoms during the day (cough, wheeze, chest tightness of breathlessness)?	☐	☐
c.	Does the asthma interfere with the patient's usual activities (housework, work, school, etc.)?	☐	☐

B6. What is the patient's smoking status?

☐ Current smoker

☐ Ex-smoker

☐ Never-smoker

B7. Does the patient have any other respiratory diseases? (Multiple responses possible)

☐ Chronic Obstructive Pulmonary Disease (COPD)

☐ Bronchiectasis

☐ Interstitial Lung Disease

☐ Other, please list:

☐ No

If you answered no to question A:

C. Do you think this patient has a history of asthma?

☐ Yes

☐ No

☐ Uncertain

Please provide anonymised copies of any additional relevant information allowing corroborating asthma diagnosis e.g. medical notes, discharge letters, test values. Payment for further information is £55 per patient.

Please return responses to CPRD in the freepost envelope provided or to our freepost address:

Freepost RSKH-TTAU-UKKX, CPRD, MHRA,

151 Buckingham Palace Rd, London, SW1W 9SZ

For peer review only

ISAC APPLICATION FORM
PROTOCOLS FOR RESEARCH USING THE CLINICAL PRACTICE RESEARCH DATALINK (CPRD)

ISAC use only: Protocol Number Date submitted	....	**IMPORTANT** **If you have any queries, please contact ISAC Secretariat:** ISAC@cprd.com

Section A: The study

1. Study Title
Validation of the recording of asthma diagnosis in adult patients in the Clinical Practice Research Datalink

2. Has any part of this research proposal or a related proposal been previously submitted to ISAC?
Yes ☐ No ☒
If Yes, please provide previous protocol numbers:

3. Has this protocol been peer reviewed by another Committee? (e.g. grant award or ethics committee)
Yes ☒ No ☐
If Yes, please state the name of the reviewing Committee(s) and provide an outline of the review process and outcome: Internal review by GSK, PRF committee

4. Type of Study (please tick all the relevant boxes which apply)

Adverse Drug Reaction/Drug Safety ☐
Drug Effectiveness ☐
Health/Public Health Services Research ☒
Drug Utilisation ☐
Pharmacoeconomics ☐
Disease Epidemiology ☒
Methodological ☒
Post-authorisation Safety ☐
Other* ☐
*Please specify the type of study in the lay summary

5. This study is intended for (please tick all the relevant boxes which apply)**:**

Publication in peer reviewed journals ☒
Presentation at company/institutional meetings ☒
Other
Presentation at scientific conference ☒
Regulatory purposes ☐

Section B: The Investigators

6. Chief Investigator (full name, job title, organisation name & e-mail address for correspondence- see guidance notes for eligibility)
Jennifer Quint, Clinical Senior lecturer in Respiratory epidemiology, Imperial College London, j.quint@imperial.ac.uk
CV has been previously submitted to ISAC ☒ **CV number:** 042_15CEPSL
A new CV is being submitted with this protocol ☐
An updated CV is being submitted with this protocol ☐

7. Affiliation (full address)
Department of NCDE, LSHTM, Keppel Street, London WC1E 7HT

8. Corresponding Applicant
Francis Nissen, PhD researcher, LSHTM, francis.nissen@lshtm.ac.uk
Same as chief investigator ☐
CV has been previously submitted to ISAC ☒ **CV number:** 449_15S
A new CV is being submitted with this protocol ☐
An updated CV is being submitted with this protocol ☐

9. List of all investigators/collaborators (*please list the full names, affiliations and e-mail addresses* of all collaborators, other than the Chief Investigator*)

Other investigator: Ian Douglas, Senior lecturer, LSHTM, ian.douglas@lshtm.ac.uk
CV has been previously submitted to ISAC ☒ **CV number:** 157_15CESL
A new CV is being submitted with this protocol ☐
An updated CV is being submitted with this protocol ☐

Other investigator: Liam Smeeth, LSHTM, Liam.Smeeth@lshtm.ac.uk
CV has been previously submitted to ISAC ☒ **CV number:** 045_15CEPSL
A new CV is being submitted with this protocol ☐
An updated CV is being submitted with this protocol ☐

Other investigator: Hana Müllerova, GSK, hana.x.muellerova@gsk.com
CV has been previously submitted to ISAC ☒ **CV number:** 365_15E
A new CV is being submitted with this protocol ☐
An updated CV is being submitted with this protocol ☐

For peer review only

For peer review only - http://bmjopen.bmj.com/site/about/guidelines.xhtml

Other investigator: Daniel Morales, University of Dundee, d.r.z.morales@dundee.ac.uk
CV has been previously submitted to ISAC ☒ **CV number:** 450_15P
A new CV is being submitted with this protocol ☐
An updated CV is being submitted with this protocol ☐

Other investigator: Neil Pearce, LSHTM, Neil.Pearce@lshtm.ac.uk
CV has been previously submitted to ISAC ☒ **CV number:** 367_15CS
A new CV is being submitted with this protocol ☐
An updated CV is being submitted with this protocol ☐

[Please add more investigators as necessary] **Please note that your ISAC application form and protocol **must** be copied to all e-mail addresses listed above at the time of submission of your application to the ISAC mailbox. Failure to do so will result in delays in the processing of your application.*

10. Conflict of interest statement* (please provide a draft of the conflict (or competing) of interest (COI) statement that you intend to include in any publication which might result from this work)

All authors have completed the ICMJE uniform disclosure at www.icmje.org/coi_disclosure.pdf and declare:
FN has received a PhD scholarship from GSK
Dr Quint reports grants from MRC, GSK, BLF, Wellcome. Personal fees from AZ, GSK.
ID has consulted for and holds stock in GSK
**Please refer to the International Committee of Medical Journal Editors (ICMJE) for guidance on what constitutes a COI*

11. Experience/expertise available (please complete the following questions to indicate the experience/expertise available within the team of investigators/collaborators actively involved in the proposed research, including the analysis of data and interpretation of results

	Previous GPRD/CPRD Studies	Publications using GPRD/CPRD data
None	☐	☐
1-3	☐	☐
> 3	☒	☒

	Yes	No
**Is statistical expertise available within the research team?** *If yes, please indicate the name(s) of the relevant investigator(s)* Ian Douglas	☒	☐
**Is experience of handling large data sets (>1 million records) available within the research team?** *If yes, please indicate the name(s) of the relevant investigator(s)* Ian Douglas, Jennifer Quint, Liam Smeeth, Daniel Morales	☒	☐
**Is experience of practising in UK primary care available within the research team?** *If yes, please indicate the name(s) of the relevant investigator(s)* Liam Smeeth, Daniel Morales	☒	☐

12. References relating to your study

Please list up to 3 references (most relevant) relating to your proposed study:

Quint JK, Müllerova H, DiSantostefano RL, Forbes H, Eaton S, Hurst JR, Davis K, Smeeth L.: Validation of chronic obstructive pulmonary disease recording in the Clinical Practice Research Datalink (CPRD-GOLD). BMJ Open. 2014 Jul 23;4(7)

Cornish RP, Henderson J, Boyd AW, Granell R, Van Staa T, Macleod: Validating childhood asthma in an epidemiological study using linked electronic patient records. J. BMJ Open. 2014 Apr 23;4(4)

For peer review only - http://bmjopen.bmj.com/site/about/guidelines.xhtml

British Thoracic Society Scottish Intercollegiate Guidelines Network. British guideline on the management of asthma. Thorax 2008;63(Suppl 4):iv1–121.

Section C: Access to the data

13. Financial Sponsor of study

Pharmaceutical Industry	☒ *Please specify:* GSK	Academia	☐ *Please specify:*
Government / NHS	☐ *Please specify:*	Charity	☐ *Please specify:*
Other	☐ *Please specify:*	None	☐

14. Type of Institution carrying out the analyses

Pharmaceutical Industry	☐ *Please specify:*	Academia	☒ *Please specify:* LSHTM
Government Department	☐ *Please specify:*	Research Service Provider	☐ *Please specify:*
NHS	☐ *Please specify:*	Other	☐ *Please specify:*

15. Data source

The sponsor has direct access to CPRD GOLD and will extract the relevant data* ☒

A data set will be supplied by CPRD** ☐

CPRD has been commissioned to extract the relevant data and to perform the analyses ☐
Other ☐ *Please specify:*

*If data sources other than CPRD GOLD are required, these will be supplied by CPRD
** Please note that datasets provided by CPRD are limited in size. Applicants should contact CPRD (KC@CPRD.com) if a dataset of >300,000 patients is required.

16. Primary care data (please specify which primary care data set(s) are required)

Vision only (Default for CPRD studies) ☒
EMIS® only* ☐
Both Vision and EMIS®* ☐

Note: Vision and EMIS are different clinical systems, Vision data has traditionally been used for CPRD, EMIS is currently undergoing beta-testing.
**Investigators requiring the use of EMIS data must discuss the study with a member of CPRD staff before submitting an ISAC application*
Please list below the name of the person/s at the CPRD with whom you have discussed your request for EMIS data:

Section D: Data linkage

17. Does this protocol also seek access to data held under the CPRD Data Linkage Scheme?

Yes* ☒ No ☐

If No, please move to section E.

**Investigators requiring linked data must discuss the study with a member of CPRD staff. It is important to be aware that linked data are not available for all patients in CPRD GOLD, the coverage periods for each data source may differ and charges may be applied. Please contact the CPRD Research Team on +44 (20) 3080 6383 or email kc@cprd.com to discuss your requirements before submitting your application.*
Please list below the name of the person/s at the CPRD with whom you have discussed your request:

Please note that as part of the ISAC review of linkages, the protocol may be shared - in confidence - with a representative of the requested linked data set(s) and summary details may be shared - in confidence - with the Confidentiality Advisory Group of the Health Research Authority.

For peer review only - http://bmjopen.bmj.com/site/about/guidelines.xhtml

18. Please select the source(s) of linked data being requested:

☐ ONS Mortality Data
☐ Inpatient Hospital Episode Statistics
☐ Outpatient Hospital Episode Statistics
☐ NCDR Cancer Registry Data*
☐ MINAP
☐ Mother Baby Link

☒ Index of Multiple Deprivation
☐ Townsend Score
☐ Other** *Please specify:*

We have discussed the data linkages with Rachael Williams, Research Statistician at CPRD.

Please note that applicants seeking access to cancer registry data must provide consent for publication of their study title and study institution on the UK Cancer Registry website. They must also complete a* ***Cancer Dataset Agreement Form* *(available from CPRD) and provide a* ***System level Security Policy*** *for each organisation involved in the study.*

*** If "Other" is specified, please name an individual in CPRD that this linage has been discussed with.*

19. Total number of linked datasets requested including CPRD GOLD: 1

20. Is linkage to a local dataset with <1 million patients being requested?

Yes* ☐ No ☒

** If yes, please provide further details:*

21. If you have requested linked data sets, please indicate whether the Chief Investigator or any of the collaborators listed in response to question 5 above, have access to any of the linked datasets in a patient identifiable form, or associated with a patient index.

Yes* ☐ No ☒

** If yes, please provide further details:*

22. Does this study involve linking to patient *identifiable* data from other sources?

Yes ☐ No ☒

Section E: Validation/verification

23. Does this protocol describe a purely observational study using CPRD data (this may include the review of anonymised free text)?

Yes* ☒ No** ☐

** Yes: If you will be using data obtained from the CPRD Group, this study does not require separate ethics approval from an NHS Research Ethics Committee.*
*** No: You may need to seek separate ethics approval from an NHS Research Ethics Committee for this study. The ISAC will provide advice on whether this may be needed.*

24. Does this study require anonymised free text?

Yes* ☐ No ☒

**Please note that work involving free text can only be performed on the July 2013 CPRD GOLD database build or earlier versions. CPRD can provide further advice on the use of anonymised free text.*

25. Does this protocol involve requesting any additional information from GPs?

Yes* ☒ No ☐

** Please indicate what will be required:*

Completion of questionnaires by the GP^ψ^	Yes ☒	No ☐
Provision of anonymised records (e.g. hospital discharge summaries)	Yes ☒	No ☐
Other (please describe)		

ψ Any questionnaire for completion by GPs or other health care professional must be approved by ISAC before circulation for completion.

For peer review only - http://bmjopen.bmj.com/site/about/guidelines.xhtml

**26. Does this study require contact with patients in order for them to complete a questionnaire?**<br><br>Yes* ☐ No ☒<br><br>**Please note that any questionnaire for completion by patients must be approved by ISAC before circulation for completion.*
**27. Does this study require contact with patients in order to collect a sample?**<br><br>Yes* ☐ No ☒<br><br>** Please state what will be collected:*
**Section F: Signatures**
**28. Signature from the Chief Investigator**<br><br>I confirm that the above information is to the best of my knowledge accurate, and I have read and understood the guidance to applicants.<br><br>Name: Jennifer Quint Date: 08/12/2015 E. signature (type name): Jennifer Quint

For peer review only

For peer review only - http://bmjopen.bmj.com/site/about/guidelines.xhtml

Protocol Section

The following headings **must** be used to form the basis of the protocol. Pages should be numbered. All abbreviations must be defined on first use.

A. Lay Summary (Max. 200 words)

This study will investigate the recording of the diagnosis of asthma in the primary care medical records database called Clinical Practice Research Datalink (CPRD GOLD). This will be done by the collection of information provided by general practitioners through a questionnaire. This information will then be examined by two independent expert physicians, giving a reliable diagnosis to be compared with the recording of asthma within the CPRD database. The diagnosis of asthma is mostly based on a characteristic pattern of symptoms and the absence of another diagnosis. Because of this, asthma is not as well defined as some other diseases, and the clinical diagnosis might be less accurate. The study to be undertaken could help establish the best strategy to identify individuals with asthma within the CPRD. This would inform the definitions and patient selection for further observational and potentially pragmatic intervention studies in CPRD and other primary care data sources.

B. Technical Summary (Max. 200 words)

The overall aim of this study is to determine the positive predictive value (PPV) of different algorithms using asthma diagnostic Read codes within the CPRD GOLD, i.e., a proportion of true positives among those assumed to have been diagnosed with asthma. In order to achieve this we will construct a retrospective cohort of asthma patients and compare database information (CPRD GOLD and the Multiple Deprivation Index) with information gathered by a questionnaire filled in by general practitioners and review of any supporting information sent. A review of these questionnaires by two independent expert physicians will be considered as the gold standard to assess the PPV of an asthma recording using specific algorithms in CPRD GOLD.

C. Objectives, Specific Aims and Rationale

(i) *Aim:*
To assess strategies to identify asthma patients of adults in United Kingdom electronic primary care records.

(ii) *Objectives:*
To determine the PPV of the recording of asthma diagnosis of adults within the CPRD GOLD database.

(iii) *Rationale:*
We will measure the level of accuracy, using the PPV, of an asthma diagnosis recording in the CPRD database employing a gold standard comprised of the review of general practitioners questionnaires by two independent experts. By doing so, we will be able to assess how reliable an asthma diagnosis is in electronic primary care records.

For peer review only - http://bmjopen.bmj.com/site/about/guidelines.xhtml

D. Background

Asthma is difficult to assess in health-care database epidemiological studies as the diagnostic criteria are based on non-specific respiratory symptoms and variable expiratory airflow limitation which are often not recorded in electronic medical records (1). According to the current estimates of the Global Burden of Disease Study 2013, 334 million people worldwide have asthma. 8.6% of young adults (aged 18-45) experience asthma symptoms and 4.5% of young adults worldwide have been diagnosed with asthma and/or are taking treatment for asthma (2). In the UK, 5.4 million people are currently receiving treatment for asthma, of whom 4.3 million are adults (3).

The British guideline on the management of asthma states that the diagnosis in adults is based on the recognition of a characteristic pattern of symptoms and signs and the absence of an alternative explanation. Based on clinical features that either increase or decrease the probability of asthma, patients are categorized in the “low”, “intermediate” or “high” probability groups. The diagnosis is then confirmed or rejected based on spirometry and/or a trial of treatment with corticosteroids (1).

Chronic obstructive pulmonary disorder (COPD), another respiratory obstructive disease that has a lot of symptoms in common with asthma can be identified with high PPV from the CPRD datasets using diagnostic Read codes alone (PPV=80%) or combined with COPD medications (PPV=90%) (4). The characteristic of COPD that best distinguishes it from asthma is the degree of reversibility of airflow obstruction, which is a central question in the questionnaire to be sent out to the GP’s (see appendix 2).

As the clinical examination necessary for the diagnosis of asthma is time and resource demanding, it would be useful for epidemiological studies to be able to obtain accurate records of asthma diagnosis within electronic databases of health-care records. The goal of this study is to understand and quantify how accurate asthma recording is in CPRD. When subsequent studies would be performed, it will be better understood how well the data reflects true diagnoses of asthma. A validation study of childhood asthma using General Practice Research Database (GPRD) data by using parental reports of a doctor’s diagnosis as the gold standard has been conducted and found a high sensitivity and specificity (5). A different study in Canada has validated asthma in patients older than 16 by comparing different information fields in electronic primary healthcare records without an external comparison (6). The CPRD database has been used in asthma studies because it captures a broad range of patients and goes back a long time. The current study will focus on the accuracy of asthma diagnosis recording in adults in CPRD, by measuring the PPV of different algorithms within the CPRD database and comparing it to a gold standard diagnosis given by the review of the answers of the GP questionnaire.

E. Study Type

This is a methodological study.

F. Study Design

This is a validation study of strategies or algorithms to ascertain asthma diagnosis recordings conducted in a retrospective cohort of asthma patients from the CPRD GOLD.

For peer review only - http://bmjopen.bmj.com/site/about/guidelines.xhtml

The random sample of individuals to be included in the study will be constructed from all participants registered in CPRD on or after 1 January 2004 who meet the inclusion criteria (see below). For the main analysis, a patient will be able to contribute to one algorithm only if an asthma medcode was recorded within the 24 month window prior to the end of data collection. It is possible an individual will be eligible for more than one algorithm depending on the Read codes used in their medical record. The individuals will be randomly selected from the algorithm with the fewest participants first and then removed from the cohort so that they cannot be selected for another algorithm. We have chosen this strategy (as opposed to an individual being eligible for a single algorithm only) because we want to test strategies to identify asthma patients from a single cohort rather than to test validity of the diagnosis. Further studies could then use a single strategy or their combination to extract an asthma cohort. There will be no special measures to ensure less frequent Read codes are used, because we assume the validity of asthma diagnosis strategy would be not be different between common and less frequent Read codes and the quality of recording would also be comparable. In addition, less frequent Read codes are unlikely to be used in isolation; our experience with validation of COPD recordings had shown that these infrequent Read codes are usually accompanied by other types of recordings.

Sample Size

The number of records for whom an asthma monitoring plan was started (medcode 81) exceeds 500,000 and the total number of asthma-related consultations exceeds 9,000,000 in the CPRD database.

Assuming an estimated PPV of 0.85 for each algorithm and an accuracy of the PPV (95% CI ± 0.08), a sample size of 77 individuals for each algorithm is needed.
A similar study conducted for COPD had a 77.6% response rate and 73.2% of the sent questionnaires were fit to be included in the final analysis (4). Considering a random sample of fully completed responses of 77 asthma patients for 8 algorithms is needed with 15% extra to account for a potential lower response rate, 750 questionnaires in total will be sent.

G. Data Linkage Required (if applicable)

The data linkage of CPRD-GOLD to MDI (Multiple Deprivation Index) is required to gather more information on the socio-economic status of the studied records. Ideally the MDI would be on patient level, if this is not available then the MDI on practice level would be used. We wish to request access to the IMD data linked to both the postcode of the GP practice and the patient's residential address (2010 version). We will take patient eligibility for linkage to IMD data into account when selecting our study population. We will also take the differences in methodology in IMD between the different countries of the UK into account.

H. Study Population

Inclusion Criteria

- Over 18 years old. People who become 18 after the study start can be included if they meet the criteria of an algorithm.
- Acceptable user status registered in CPRD.
- Practice is "up to standard" at study start 1/1/2004. From this date onwards, the Quality and Outcomes Framework (QOF) came in effect.

For peer review only - http://bmjopen.bmj.com/site/about/guidelines.xhtml

- The patient fits in one of the asthma algorithms within the last 24 months (see below)
- Patients are still alive and practice is currently still active in the CPRD.

Exclusion Criteria:

The patient does not fit the criteria of an algorithm group
Younger than 18 years

I. Selection of comparison group(s) or controls

There is no comparison group, as this is a validation study. The cohort will consist of only patients with a recording of asthma.

J. Exposures, Outcomes and Covariates

Exposure: Each patient included can contribute to only one algorithm or strategy (see appendix 3). If a patient is selected for a single algorithm (starting with the algorithm with the fewest participants), the patient will be excluded from the pool for the next algorithms. A preliminary code list for asthma diagnosis can also be found in appendix 1.

Covariates for stratification analysis:

-Age in years. All patients are 18 years or older, the categories will be based on the sample distribution.
-Gender as male or female
-Body Mass Index (BMI)
-Smoking status
-Other co-morbid conditions: COPD, atopy, GERD (Gastro-oesophageal Reflux Disease), eczema,rhinitis (including allergic rhinitis (hayfever) and chronic rhinosinusitis) and family history of asthma or atopy.
-Multiple deprivation Index

Outcome: recording of asthma diagnosis according to a specified algorithm and verified by the reference standard.

A number of different algorithms were constructed with degrees of certainty of asthma using separate indicators (see appendix 3). For example, the most stringent algorithm would include an asthma code, asthma medication and demonstrated reversibility after trial of treatment. Other algorithms would then drop one or more of these criteria. See appendix 3 for details of the algorithms.

A questionnaire will be sent to the general practitioners of a random sample of patients who fit in a certain algorithm to obtain information for the gold standard. A draft of the questionnaire can be found in appendix 2. The questionnaire is based on the "British guideline on the management of asthma" by the British Thoracic Society and Scottish Intercollegiate Guidelines Network (1).

K. Data/ Statistical analysis

The main analysis will be the calculation of the positive predictive value (the proportion of true positives) in each of the predefined algorithms. The gold standard consists of the opinion of 2 medical experts (Jennifer Quint and Daniel Morales) independently

For peer review only - http://bmjopen.bmj.com/site/about/guidelines.xhtml

reviewing the questionnaires and any additional supporting medical information provided. If there is a disagreement of diagnosis, the case would be discussed by the two experts. If an agreement cannot be found, a third opinion will be sought. Included in the main text.
Stratification analysis will be used to assess potential effect modification or confounding by covariates (see covariate list).

L. Plan for addressing confounding

Not applicable.

M. Plan for addressing missing data

We plan to do a complete case analysis, assuming that the probability of data being missing is independent of accuracy of the asthma diagnosis, conditional on covariates. If the amount of missing data is small, any violation of the assumption is unlikely to importantly affect the results. We anticipate a small degree of missingness for the BMI and smoking covariates.

N. Limitations of the study design, data sources and analytical methods

-Using a GP questionnaire as the source of patient information in order to obtain a gold standard to validate the asthma diagnosis can be problematic as the GP can consult the electronic health record to see if there was an asthma diagnosis. This will lead to an overestimation of the PPV. The GP's will be asked not to consult the CPRD records in the questionnaire.
-Incomplete diagnostic information will lead to missing data which we will be unaware of which could lead to some inaccuracy in PPV or classification of asthma probability.
-Only living patients will be assessed, as GP's no longer have access to the patient records after death. This excludes the records of the deceased patients and could result in survival bias.
-Miscoding accidents would lower the PPV.
-Response rate for the questionnaire might be lower than expected, and the sample size of the completed questionnaires could be too small.
-By focusing on the PPV, we will not be able to accurately assess the NPV, specificity or sensitivity. By preselecting the population of possible asthma cases, the NPV, specificity and sensitivity would be artificially manipulated. The NPV is the Negative Predictive Value: the proportion of negative results that are true negatives. -We are assuming that the validity of asthma diagnosis strategy would not be different between common and less frequent Read codes and the quality of recording would also be comparable for pragmatic reasons. In future practice when identifying patients with asthma, the less commonly used codes will continue to identify a smaller proportion of all asthma patients and so the validity we measure will apply to the majority of patients.
-We are also assuming that the probability of data being missing is independent of accuracy of the asthma diagnosis. We agree this assumption may not hold, but, we are even less likely to meet the assumptions needed for multiple imputation. However, we anticipate little missing relevant data in this study based on past research. In addition, the covariates are needed for stratification analysis only, rather than for adjustment. So we anticipate the impact of missing data to be low

For peer review only - http://bmjopen.bmj.com/site/about/guidelines.xhtml

-Not all GP practices contribute to CPRD, and patients might refuse to participate in the CPRD programme. This can result in selection bias.

O. Patient or user group involvement (if applicable)

Currently there is no plan to involve patients in the study. Depending on our findings it is possible we would seek patient engagement in further studies to help shape future research questionswith the help of general asthma patient groups.

P. Plans for disseminating and communicating study results, including the presence or absence of any restrictions on the extent and timing of publication

We will present our findings at national and international meetings and publish the results in a peer reviewed journal. We will not include any cells with counts less than five due to anonymity concerns.

Q. References

1. British Thoracic Society Scottish Intercollegiate Guidelines N. British Guideline on the Management of Asthma. Thorax. 2008;63 Suppl 4:iv1-121.
2. Global, regional, and national age–sex specific all-cause and cause-specific mortality for 240 causes of death, 1990–2013: a systematic analysis for the Global Burden of Disease Study 2013. The Lancet.385(9963):117-71.
3. NHS. http://www.nhs.uk/conditions/asthma: NHS; 2015 [cited 2015 06/11].
4. Quint JK, Mullerova H, DiSantostefano RL, Forbes H, Eaton S, Hurst JR, et al. Validation of chronic obstructive pulmonary disease recording in the Clinical Practice Research Datalink (CPRD-GOLD). BMJ Open. 2014;4(7):e005540.
5. Cornish RP, Henderson J, Boyd AW, Granell R, Van Staa T, Macleod J. Validating childhood asthma in an epidemiological study using linked electronic patient records. BMJ Open. 2014;4(4):e005345.
6. Xi N, Wallace R, Agarwal G, Chan D, Gershon A, Gupta S. Identifying patients with asthma in primary care electronic medical record systems: Chart analysis–based electronic algorithm validation study. Canadian Family Physician. 2015;61(10):e474-e83.

R. Amendment

March 2016

There were some slight changes to the questionnaire on advice from CPRD regarding the remuneration of the GP's. There were also some minor amendments to the questionnaire to clarify the procedure for returning the questionnaire and to insert the patient identifier tables we use. The sentence "To answer this questionnaire, please refrain from using the data recorded in CPRD as the aim of this study is to see how reliable CPRD is." was removed to avoid confusion.

March 2017

For peer review only - http://bmjopen.bmj.com/site/about/guidelines.xhtml

We would like to examine the additional information provided by the questionnaires sent to GP's to quantify the misdiagnosis of COPD in asthma patients in the UK. The symptoms of asthma and COPD overlap, and the differential diagnosis is not always trivial to make. Information on reversibility testing, the QOF indicators, smoking status, concurrent respiratory diseases and other sources including consultant and hospital discharge letters, lung function tests and radiography results was requested in the questionnaire (see attachment).
A review of this information by a respiratory consultant and study GP aims to identify the actual cases of COPD in confirmed asthma patients. This review is used as the gold standard to calculate the PPV, NPV, sensitivity and specificity of recorded GP diagnoses of COPD in the primary care records of asthma patients.

The specific objectives we would like to add to this study are to calculate the PPV, NPV, sensitivity and specificity of a COPD diagnosis recorded by a general practitioner in patients with a confirmed asthma diagnosis.

For peer review only

Appendices

Appendix 1: CPRD medcodes indicating asthma

medcode	readterm	Probable	Definite
78	asthma		1
81	asthma monitoring		1
185	acute exacerbation of asthma		1
232	asthma attack		1
233	severe asthma attack		1
719	h/o: asthma	1	
1208	childhood asthma	1	
1555	bronchial asthma		1
2290	allergic asthma		1
3018	mild asthma		1
3366	severe asthma		1
3458	occasional asthma		1
3665	late onset asthma		1
4442	asthma unspecified		1
4606	exercise induced asthma		1
4892	status asthmaticus nos		1
5138	patient in asthma study	1	
5267	intrinsic asthma		1
5515	seen in asthma clinic	1	
5627	hay fever with asthma		1
5798	chronic asthmatic bronchitis		1
5867	exercise induced asthma		1
6707	extrinsic asthma with asthma attack		1
7058	emergency admission, asthma		1
7146	extrinsic (atopic) asthma		1
7191	asthma limiting activities		1
7229	asthma prophylactic medication used	1	
7378	asthma management plan given		1
7416	asthma disturbing sleep		1
7731	pollen asthma		1
8335	asthma attack nos		1
8355	asthma monitored		1
9018	number of asthma exacerbations in past year		1
9552	change in asthma management plan		1
9663	step up change in asthma management plan		1
10043	asthma annual review		1
10274	asthma medication review		1

For peer review only

For peer review only - http://bmjopen.bmj.com/site/about/guidelines.xhtml

10487	asthma - currently active		1
11022	asthma trigger	1	
11370	asthma confirmed		1
11387	refuses asthma monitoring	1	
11673	excepted from asthma quality indicators: patient unsuitable	1	
11695	excepted from asthma quality indicators: informed dissent	1	
12987	late-onset asthma		1
13064	asthma severity		1
13065	moderate asthma		1
13066	asthma - currently dormant	1	
13173	asthma not disturbing sleep	1	
13174	asthma not limiting activities	1	
13175	asthma disturbs sleep frequently		1
13176	asthma follow-up		1
14777	extrinsic asthma without status asthmaticus		1
15248	hay fever with asthma		1
16070	asthma nos		1
16655	asthma monitoring admin.	1	
16667	asthma control step 2		1
16785	asthma control step 1		1
18141	asthma monitoring due	1	
18223	step down change in asthma management plan		1
18224	asthma control step 3		1
18323	intrinsic asthma with asthma attack		1
18692	exception reporting: asthma quality indicators	1	
18763	referral to asthma clinic	1	
19167	asthma monitoring by nurse		1
19519	asthma treatment compliance unsatisfactory		1
19520	asthma treatment compliance satisfactory		1
19539	asthma monitoring check done	1	
20422	asthma clinic administration	1	
20860	asthma control step 5		1
20886	asthma control step 4		1
21232	allergic asthma nec		1
22752	occupational asthma		1
24479	emergency asthma admission since last appointment		1
24506	further asthma - drug prevent.		1
24884	asthma causes daytime symptoms 1 to 2 times per week		1
25181	asthma restricts exercise		1
25705	asthma monitor 3rd letter	1	
25706	asthma monitor 2nd letter	1	
25707	asthma monitor 1st letter	1	
25791	asthma clinical management plan		1
25796	mixed asthma	1	

For peer review only - http://bmjopen.bmj.com/site/about/guidelines.xhtml

26496	health education - asthma	1	
26501	asthma never causes daytime symptoms		1
26503	asthma causes daytime symptoms most days		1
26504	asthma never restricts exercise		1
26506	asthma severely restricts exercise		1
26861	asthma sometimes restricts exercise		1
27926	extrinsic asthma with status asthmaticus		1
29325	intrinsic asthma without status asthmaticus		1
29645	asthma control step 0	1	
30308	dna - did not attend asthma clinic	1	
30382	asthma monitoring admin.nos	1	
30458	asthma monitoring by doctor		1
30815	asthma causing night waking		1
31135	asthma monitor phone invite	1	
31167	asthma night-time symptoms		1
31225	asthma causes daytime symptoms 1 to 2 times per month		1
35927	asthma leaflet given	1	
37943	asthma monitor verbal invite	1	
38143	asthma never disturbs sleep		1
38144	asthma limits walking up hills or stairs		1
38145	asthma limits walking on the flat		1
38146	asthma disturbs sleep weekly		1
39478	wood asthma		1
39570	asthma causes night symptoms 1 to 2 times per month		1
40823	brittle asthma		1
41017	aspirin induced asthma		1
41020	absent from work or school due to asthma		1
41554	asthma monitor offer default	1	
42824	asthma daytime symptoms		1
43770	asthma society member	1	
45073	intrinsic asthma nos		1
45782	extrinsic asthma nos		1
46529	attends asthma monitoring		1
47337	asthma accident and emergency attendance since last visit		1
47684	detergent asthma		1
58196	intrinsic asthma with status asthmaticus		1
73522	work aggravated asthma		1
92109	asthma outreach clinic	1	
93353	sequoiosis (red-cedar asthma)		1
93736	royal college of physicians asthma assessment		1
98185	asthma control test		1
99793	patient has a written asthma personal action plan		1
100107	health education - asthma self management		1
100397	asthma control questionnaire		1

For peer review only - http://bmjopen.bmj.com/site/about/guidelines.xhtml

100509	under care of asthma specialist nurse		1
100740	health education - structured asthma discussion		1
102170	asthma review using roy colleg of physicians three questions		1
102209	mini asthma quality of life questionnaire		1
102301	asthma trigger - seasonal		1
102341	asthma trigger - pollen		1
102395	asthma causes symptoms most nights		1
102400	asthma causes night time symptoms 1 to 2 times per week		1
102449	asthma trigger - respiratory infection		1
102713	asthma limits activities 1 to 2 times per month		1
102871	asthma trigger - exercise		1
102888	asthma limits activities 1 to 2 times per week		1
102952	asthma trigger - warm air		1
103318	health education - structured patient focused asthma discuss		1
103321	asthma trigger - animals		1
103612	asthma never causes night symptoms		1
103631	royal college physician asthma assessment 3 question score		1
103813	asthma trigger - cold air		1
103944	asthma trigger - airborne dust		1
103945	asthma trigger - damp		1
103952	asthma trigger - emotion		1
103955	asthma trigger - tobacco smoke		1
103998	asthma limits activities most days		1
105420	asthma self-management plan review		1
105674	asthma self-management plan agreed		1
106805	chronic asthma with fixed airflow obstruction		1
107167	number days absent from school due to asthma in past 6 month		1

For peer review only

For peer review only - http://bmjopen.bmj.com/site/about/guidelines.xhtml

Study into asthma: questionnaire for £55, further information for £55

The London School of Hygiene and Tropical Medicine is conducting a study to investigate the best way to identify asthma within the Clinical Practice Research Datalink (CPRD). We have developed several methods for identifying asthma in the database, and we would like to obtain some information on the current asthma status of the patient from GPs so that we can decide which method is the most suitable. We would be very grateful if you could supply us with the following information.

A. Do you agree this patient has a current diagnosis of asthma?

- ☐ Yes: Proceed to question B
- ☐ No: Proceed to question C
- ☐ Uncertain: Proceed to question B

If you answered yes or uncertain to question A:

B1. Has the diagnosis been made or confirmed by a respiratory physician?

- ☐ Yes
- ☐ No

B2. Does this patient have evidence of reversible airway obstruction?

- ☐ Yes
- ☐ No

If yes: **Was this based on;**

- ☐ Spirometry reversibility with a bronchodilator
- ☐ Trial of treatment with oral or inhaled corticosteroids and diurnal

variation on a peak flow diary

B3. In what year was the asthma first diagnosed?

B4. Were any other factors taken into consideration in making the diagnosis?

	Yes	No
a. History of atopic disorder	☐	☐
b. Family history of asthma and/or atopic disorder	☐	☐
c. Widespread wheeze heard on auscultation of the chest	☐	☐
d. Otherwise unexplained low FEV (Forced Expiratory Volume) or PEF (Peak Expiratory Flow) on spirometry	☐	☐
e. Otherwise unexplained variability in PEFR (Peak Expiratory Flow Rate) on spirometry	☐	☐
f. Otherwise unexplained peripheral blood eosinophilia	☐	☐

For peer review only - http://bmjopen.bmj.com/site/about/guidelines.xhtml

g. FeNO (Fractional exhaled Nitric Oxide) measurement ☐ ☐
h. Other (please name)

B5. Based on the QOF (Quality and Outcomes Framework) indicators:

	**Yes**	**No**
a. Does the patient have any difficulty sleeping because of asthma symptoms, including cough	☐	☐
b. Does the patient have the usual asthma symptoms during the day (cough, wheeze, chest tightness of breathlessness)?	☐	☐
c. Does the asthma interfere with the patient's usual activities (housework, work, school, etc.)?	☐	☐

B6. What is the patient's smoking status?

☐ Current smoker
☐ Ex-smoker
☐ Never-smoker

B7. Does the patient have any other respiratory diseases? (Multiple responses possible)
☐ Chronic Obstructive Pulmonary Disease (COPD)
☐ Bronchiectasis
☐ Interstitial Lung Disease
☐ Other, please list:
☐ No

If you answered no to question A:
C. Do you think this patient has a history of asthma?
☐ Yes
☐ No
☐ Uncertain

Please provide anonymised copies of any additional relevant information allowing corroborating asthma diagnosis e.g. medical notes, discharge letters, test values. Payment for further information is £55 per patient.

Please return responses to CPRD in the freepost envelope provided or to our freepost address:
Freepost RSKH-TTAU-UKKX, CPRD, MHRA,
151 Buckingham Palace Rd, London, SW1W 9SZ

For peer review only - http://bmjopen.bmj.com/site/about/guidelines.xhtml

Appendix 3: Algorithms: all within the last 24 months

1. Definite asthma code + evidence of reversibility testing (spirometry or trial of treatment) *or variable PEFR* + more than one prescription of inhaled asthma therapy *(Inhaled SABA/LABA/CS)*
2. Definite asthma code + evidence of reversibility testing (spirometry or trial of treatment) *or variable PEFR*
3. Definite asthma code + more than one prescription of inhaled asthma therapy *(Inhaled SABA/LABA/CS)*
4. Definite asthma code only
5. Possible asthma code + evidence of reversibility testing (spirometry or trial of treatment) *or variable PEFR* + more than one prescription of inhaled asthma therapy *(Inhaled SABA/LABA/CS)*
6. Symptoms (wheeze, breathlessness, chest tightness, cough) + evidence of reversibility testing (spirometry or trial of treatment) *or variable PEFR* + more than one prescription of inhaled asthma therapy *(Inhaled SABA/LABA/CS)*
7. Symptoms (wheeze, breathlessness, chest tightness, cough) + evidence of reversibility testing (spirometry or trial of treatment) or variable PEFR
8. Symptoms (wheeze, breathlessness, chest tightness, cough) + more than one prescription of inhaled asthma therapy *(Inhaled SABA/LABA/CS)*

For peer review only

For peer review only - http://bmjopen.bmj.com/site/about/guidelines.xhtml
